# Biogeographical patterns of amphibians and reptiles in the northernmost coastal montane complex of South America

**DOI:** 10.1371/journal.pone.0246829

**Published:** 2021-03-04

**Authors:** Gilson A. Rivas, Oscar M. Lasso-Alcalá, Douglas Rodríguez-Olarte, Mayke De Freitas, John C. Murphy, Cristian Pizzigalli, John C. Weber, Laurent de Verteuil, Michael J. Jowers

**Affiliations:** 1 Facultad Experimental de Ciencias, Museo de Biología, La Universidad del Zulia, Maracaibo, Estado Zulia, Venezuela; 2 Museo de Historia Natural La Salle (MHNLS), Fundación La Salle de Ciencias Naturales (FLASA), Caracas, Venezuela; 3 Museo de Ciencias Naturales, Decanato de Agronomía, Universidad Centro Occidental Lisandro Alvarado, UCLA, Barquisimeto, Estado Lara, Venezuela; 4 Independent Researcher, Waterbeach, Cambridge, United Kingdom; 5 Science and Education, Field Museum, Chicago, IL, United States of America; 6 CIBIO/InBIO (Centro de Investigação em Biodiversidade e Recursos Genéticos), Universidade do Porto, Vairão, Portugal; 7 Department of Geology, Grand Valley State University, Allendale, MI, United States of America; 8 Independent Researcher, London, ON, Canada; Universitat Trier, GERMANY

## Abstract

We examine, for the first time, biogeographic patterns in a series of tropical montane coastal systems in northern South America. We use amphibians and reptiles, which constitute the most critical communities based upon the prevalence of endemic taxa, to assess the region’s biodiversity. The montane coastal system spans an east-west distance of 925 km. It includes peaks ranging from 549 m to 2765 m above sea level and encompasses the montane complexes of northern Venezuela (including Isla de Margarita), an outlier at Santa Marta (Colombia), and ranges on the islands Trinidad and Tobago. The area supports 14 family level amphibian clades and 23 family level reptile clades. Fieldwork, museum specimen surveys, and a literature review suggest that biodiversity decreases at higher elevations. Here we examine the biogeographic patterns in the region to assess the role of the montane systems as possible refugia. We also look at the possible island and sky island effects using data from altitudes >200 m. At lower elevations, we tabulated 294 species, comprising 112 amphibians and 182 reptiles. About 45% of these taxa are endemic or exclusive to different sub-regions. At mid-elevation montane cloud forests, we find a much-reduced biodiversity with a total of 125 species (66 amphibians and 59 reptiles) exclusive or restricted to the region, and few species shared between systems. We find that biogeographical patterns follow a natural topographic disposition above 200 m in elevations. At the lower elevation cut off, there are 118 species (26 amphibians and 92 reptiles) shared among two or more of the studied mountain systems, suggesting a common origin and dispersal events, despite what seem to be topographic barriers. Biogeographical relationships support a topographic disposition of the region with close associations between the islands of Trinidad and Tobago, the Paria Range and the Turimiquire Massif, and close associations between the Sierra Nevada de Santa Marta and the Sierra de San Luis. Overall, the biogeographic relationships between amphibians and reptiles are similar. Species diversity in the eastern Caribbean region is less rich than in the west. This study includes the first herpetological surveys at the two easternmost mountains (Cerro La Cerbatana and Campeare) belonging to the Paria Range biogeographic unit, and aims to contribute to a better understanding of the rich biodiversity of the region.

## 1 Introduction

Topography is an essential factor in speciation and biodiversity hotspots are frequently associated with mountain ranges that produce a sky-island effect. Sky-island is a term coined to refer to places where the slopes and summit of a mountain exhibit dramatically different ecosystems from the surrounding lowlands [1–3]. A diverse group of organisms occurs in northern South America´s coastal montane complex, including: frogs [3–6] mammals [7–9], lizards [10–12], snakes [13–15] birds [16–18], invertebrates [19–21] and plants [22,23]. Turchetto-Zolet et al. [24] reviewed phylogeographic studies on South American species between 1987 and 2011, concluding that the interactions between the oscillating Pleistocene climatic and Pliocene/Miocene mountain building events had shaped much of the continent’s biodiversity and that a substantial portion of the diversity remains undocumented [25–30]. Although the Venezuelan montane coastal systems have been termed sky islands in the literature [9], the characteristics of some parts of these systems do not fully comply with the sky island definition of Warshall (1974) [2] and we therefore refer to them here as coastal montane complex.

**Fig 1:**
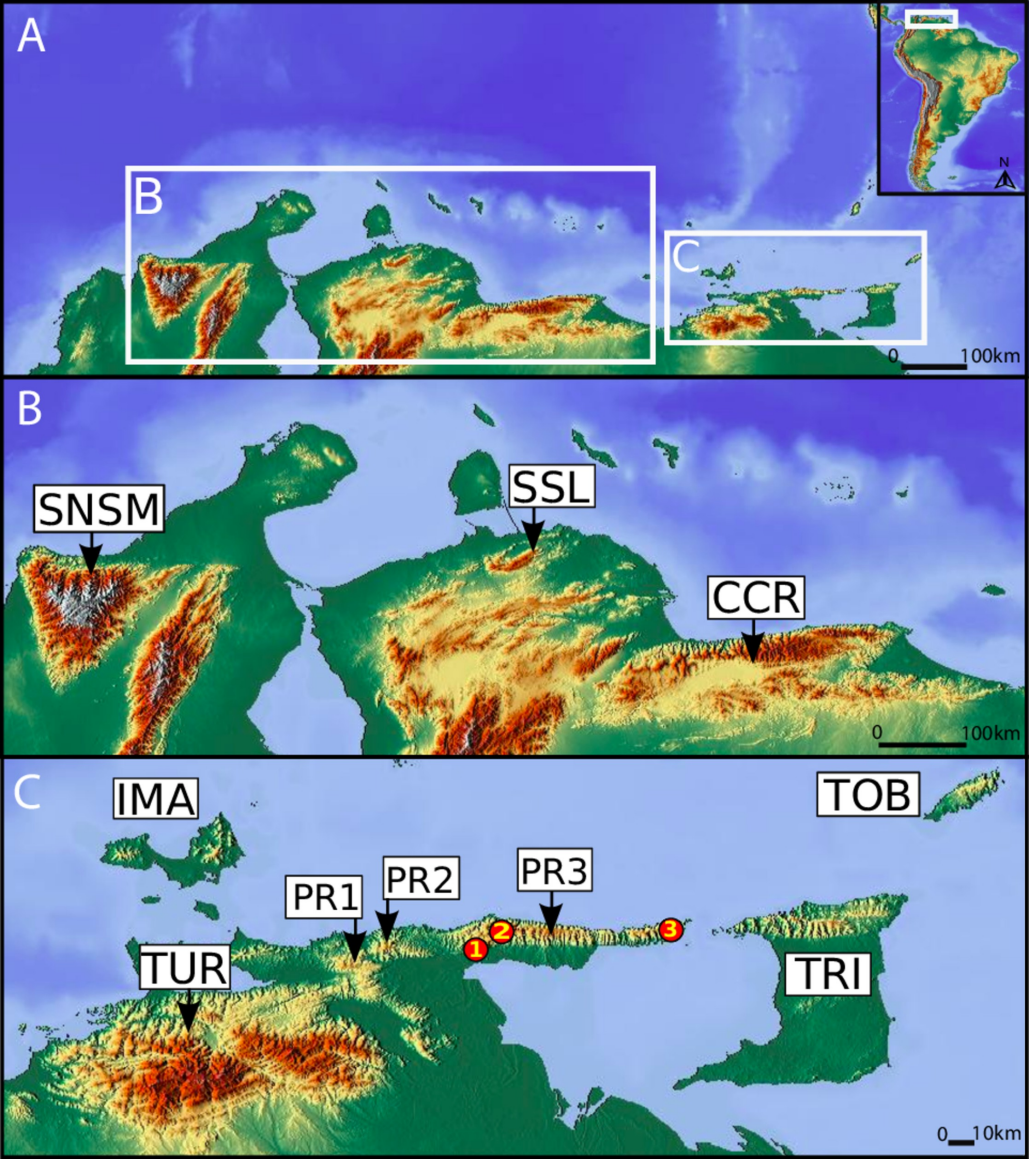
Map of northern South America showing from west to east: Sierra Nevada de Santa Marta (SNSM, Colombia), Sierra de San Luis (SSL), Cordillera de la Costa Central or Central Coastal Range (CCR), Turimiquire Massif (TUR), mountains of Isla de Margarita (IMA), and on the Eastern Coastal Range the Paria Range (PR) as well as Trinidad Northern Range (TRI) and Tobago Main Ridge (TOB). Arrows indicate the main mountainous system in these areas, which are referred in Tables 1–4, Figs 3–6. Although Cerro Campeare (PR1) and Cerro La Cerbatana (PR2) peaks lay outside the proper Paria Peninsula or Paria Range (PR3), both peaks share Paria’s herpetological fauna as confirmed in this work. Numbers in red circles are: 1) Cerro Cachipal, 2) Cerro Humo and Cerro Las Melenas, 3) Macuro. Map build with GIS Cloud Apps (www.giscloud.com). Reprinted from Relief Free Map under a CC BY license, with permission from the Open Data Commons Open Database License (ODbL) by the OpenStreetMap Foundation (OSMF), original copyright OpenStreetMap^®^.

**Fig 2:**
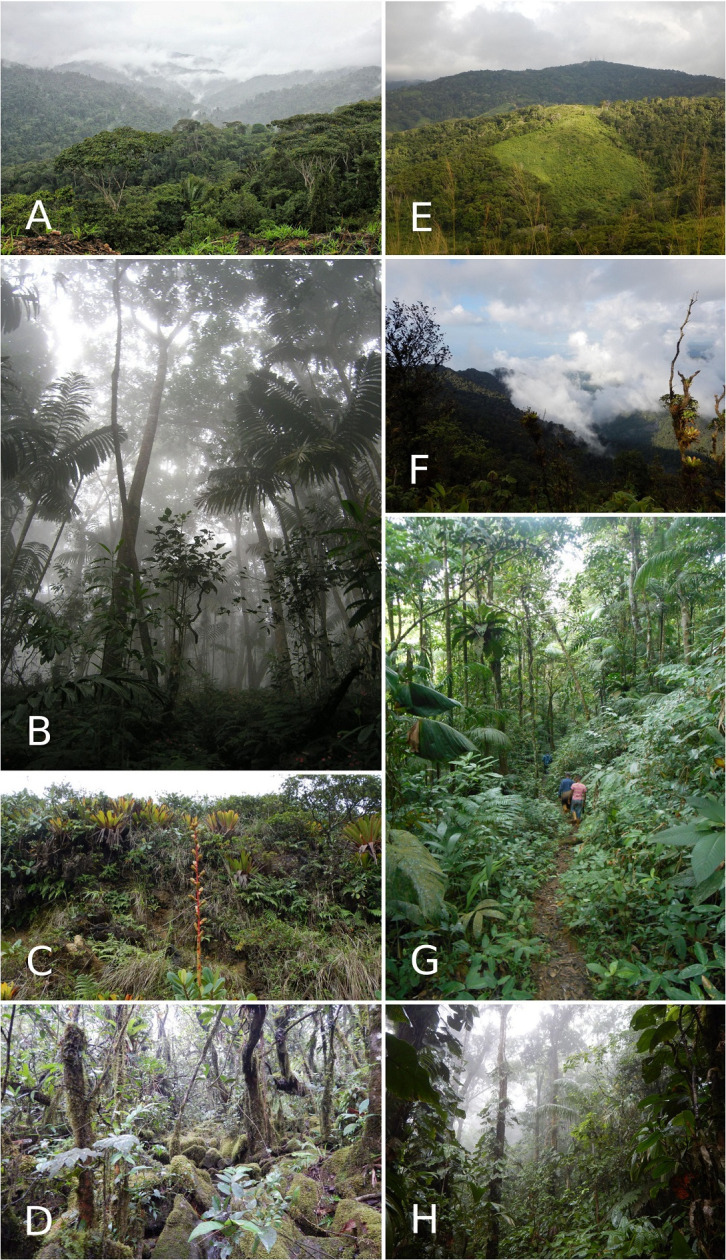
Vegetation in Venezuela’s northernmost montane coastal systems. A. General view of the evergreen montane forest in Sierra de Aroa on the western side of Central Coastal Range Yaracuy state. B. Cloud forest in the Central Coastal Range. Monumento Natural Pico Codazzi, Aragua state. C. Summit of Cerro El Copey on Isla de Margarita. *Glomeropitcairnia erectiflora* mostly grows on the ground in Copey’s Summit. D. Elfin forest around 700 m in elevation in Cerro El Copey. E. Evergreen forest in Cerro La Cerbatana. F. View of the top of Cerro Campeare G. Cerro Las Melenas and Cerro El Padre seen from Cerro Humo summit. H. Cloud forest surrounding the town of Las Melenas (750 m), near Cerro Humo. Photos by Helga Terzenbach (A), Aurelien Miralles (B), Gilson Rivas (C-F, H), Luis Sibira (G).

In northern Venezuela, the Venezuelan Coastal Range or Cordillera de la Costa (CC) is a mountain range that extends 780 km (63,000 km^2^) in an east-west orientation. If the outliers of the Venezuelan Isla de Margarita, Trinidad’s Northern Range, the Main Ridge on the island of Tobago, and the Sierra Nevada de Santa Marta, all of which are associated with the adjacent Caribbean region, are also included, the east-west distance of this coastal montane complex increases to about 1500 km [31] (Fig 1). The CC’s high elevation peaks are patchy and therefore many areas of upper elevation with moist forest are isolated from the lower, drier topography that surrounds river valleys, and saltwater barriers [32,33] (Fig 2). Rising and falling sea levels, changing climate, and tectonic events have shaped the landscape and have repeatidly isolated and reconnected multiple populations and communities of organisms [34]. The CC is divided into west (Central Coastal Range, CCR) and east ranges (Eastern Coastal Range, ECR) separated by the Unare depression, which has likely acted as a barrier for amphibian and reptile migration [35].

The “Serranía de Paria”, here refered to as the Paria Range, constitues the Paria Peninsula; and lies within the Venezuelan CC bioregion; its biological diversity is only moderately well known [36]. Species surveys and inventories, particularly those done during the last two decades, have revealed a high diversity with local endemism, highlighting the need for additional studies of the Paria Range’s biota to help better understand the region’s biogeography [36,37]. Paria’s diverse herpetofauna likely results from the cloud forest habitat on most summits of this high elevation mountain complex. There are two outlier peaks nearby associated with the Paria range: the Cerros Campeare and La Cerbatana (Fig 1). The discontinuity and remoteness of La Cerbatana have deterred exploration by scientists, and Campeare’s fauna remains almost completely unexplored. New species of animals and plants have recently been discovered in these isolated mountains [38,39]. Results from expeditions at La Cerbatana by Wilfried Meier [40] and subsequent surveys in 2013 by two of us (GAR and MDF), constitute what is known of the vegetation and herpetofauna at these two localities.

The “Península de Paria National Park” legally protects most of the Paria Range’s cloud forests. Yet, the laws are not robust enough, nor sufficiently enforced, to protect the flora and fauna on some of its most isolated peaks. The summit of Campeare is completely deforested; this is in stark contrast with the lush cloud forest that still caps La Cerbatana (Fig 2). Isolated peaks in northeastern Venezuela, i.e. Paria’s summits, and Cerro El Copey on Isla de Margarita, share some endemic taxa. These taxa may provide key evidence to understand the biogeographic patterns in the region and on the islands of the eastern Caribbean [41]. The islands of Trinidad, Tobago, and Margarita were part of the same landscape during the last glacial maxima at 15–20 kya when sea levels were approximately 116 m lower than they are today [42]. Miocene and Pliocene-Pleistocene connections across the region during other low sea level stands suggest that repeated and prolonged periods of faunal interchange between the islands and the mainland may account for some of the similarities in biotic composition across the region [43–47].

This study aims to establish updated, region-wide, faunal lists for amphibians and reptiles and then to use these to assess biogeographical patterns of their distribution. We also aim to demonstrate how individual montane segments are related based on their species compositions; examine the herpetofauna and its richness on the Paria Peninsula; assess the relationships of adjacent Venezuelan biogeographic regions, including the: Sierra de San Luis, Central Coastal Range, Turimiquire Massif, the mountains on the islands of Margarita, Trinidad and Tobago, and the Sierra Nevada de Santa Marta, Colombia. From a biogeographic perspective, we also investigate the Paria Range to determine whether it forms a distinct biogeographic unit. Additionally, we compare the herpetofauna at low- to mid-level altitudes to those at higher elevations to determine whether a sky island effect (i.e., higher biodiversity on mountain sides) is present. These data are pivotal for future conservation management planning in the region for prioritizing those areas with the highest species richness and biodiversity. The coastal Sierra Nevada de Santa Marta, Colombia is included in this study because of its proximity to the Caribbean, but its geography, tectonic history and fauna are quite different from those of the ranges and peaks of the CC in Venezuela. The Sierra Nevada de Santa Marta is at the northermost extend of the Andes and thus serves as an outgroup for the ranges to the east. This is the first study of this scope that has been done for this region.

## 2 Methods and materials

The species compositions of the herpetofauna for the Sierra Nevada de Santa Marta, most of the Central Coastal Range, Sierra San Luis, Turimiquire Massif, Isla de Margarita and Trinidad and Tobago were based on museum specimens and the on literature, much of which was collected and written by authors of this work (GAR, JCM, MJJ). However within the Paria Range, all data on the presence of reptiles and amphibians on the mountains (the so called “Cerros”) of the Cerbatana and Campeare are from surveys we (GAR, MDF, OL-A) conducted and report on here for the first time.

### 2.1 Ethics statement

Collection permits number 0878 and 1375 were granted to Gilson A. Rivas by the Ministerio del Poder Popular para el Ecosocialismo y Aguas, Venezuela. This study was carried out in strict accordance with the recommendations in the “Guidelines for the use of live amphibians and reptiles in the field and laboratory research”, Second Edition, Revised by the Herpetological Animal Care and Use Committee (HACC) of the American Society of Ichthyologists and Herpetologists (ASIH), 2004. Animals were euthanized using the methods and following approved ASIH guidelines: adult reptiles were euthanized with pentobarbital; amphibians were euthanized using MS-222 in water, or by ventral application of Orajel® (a 20% benzocaine gel).

### 2.2 Surveyed areas (La Cerbatana and Campeare)

The primary field study area is located in northeastern Venezuela (about 550 km East of Caracas) within the Paria Peninsula (10° 42’ N, 62° 37’ W), which forms a narrow strip of high land aligned geologically and geographically with Trinidad´s Northern Range (Fig 1). The Caribbean Sea is north of the Paria Peninsula, and the Gulf of Paria is south of the peninsula. The range is oriented in an east-west direction with elevations that range up to 1250 m (Cerro Humo). The Paria Range forms a major segment of Venezuela’s Coastal Range (“Cordillera de La Costa”: CC), a chain of mountains extending eastward from the Andes for about 925 km into Trinidad [31].

Surveys of the Paria Range are a key part of this study; thus a brief description of the unexplored Cerro La Cerbatana and Campeare is given here. At least the Cerbatana, had not been studied prior the visits of botanist Winfred Meier [40] and ornithologist Gedio Marín [48], and three of us (GAR, MDF, OL-A). Campeare and La Cerbatana are separated by 18 km, and another ~60–100 km separates each of them from the Paria Range. Cerro La Cerbatana is separated from the Paria Range by coastal lowlands and a distance of about 100 km; the elevations in this intervening area are not higher than 250 m [39]. At its summit (1000 m), a remnant cloud forest is present, which resembles in many aspects the high-elevation forests found on many of Paria’s summits and on the mountain peaks in Trinidad’s Northern Range. The endemic and rare tank bromeliad *Glomeropitcairnia erectiflora* is shared between Trinidad’s Northern Range and the Paria Range [37]. Along its northern slope, the habitat of Cerro La Cerbatana has undergone significant degradation. Campeare´s highest elevation is 1000 m. This mountain, also isolated from Paria’s summits, has been almost completely deforested. Little of its summit and slopes are covered by its original cloud forest, and secondary coffee plantations dominate its slopes.

### 2.3 Surveys and updated records

Eleven surveys were conducted at Cerros La Cerbatana, Campeare, Cachipal, Humo and Macuro and its surroundings; some localities were surveyed more than once (S1 Table). These surveys were used to assess the composition of the herpetofauna at the summits (800–1250 m asl) and at medium elevations (500–800 m asl). We calculated our survey effort in person-time per site. A total of 80 specimens belonging to 20 species were recorded (S1 Text). Samples of muscle or liver were collected from cryptic species to corroborate field identifications through DNA anlayses (S2 Table). Surveys were conducted from 0800 to 2100 h, daily by two or three researchers, using visual encounters, and during diurnal and nocturnal walks to find and capture specimens [49] (S1 Table). All specimens were deposited in the herpetological collections of the Museo de Biología, La Universidad del Zulia, Maracaibo (MBLUZ) and the Estación Biológica de Rancho Grande, Maracay (EBRG). (S1 Table) contains a compilation of the number of surveys in the Paria Region, survey dates, species collected, and survey efforts.

To summarize the information on the species we obtained in the field, we constructed a taxonomic list of species (S3 and S4 Tables); performed an exhaustive bibliographical review (S2 Text); and examined Venezuelan museum collections, including those of the Museo de Ciencias Naturales, Caracas (MCNC), Museo de la Estación Biológica de Rancho Grande, Maracay (EBRG), Museo de Biología, Universidad Central de Venezuela, Caracas (MBUCV) and Museo de Biología, Universidad del Zulia, Maracaibo (MBLUZ). We updated the main international biodiversity database (Global Biodiversity Information Facility: www.gbif.org), and categorize species according to their distributions; for example, an exclusive species is defined as that restricted to only one of the eight regions within our study area. However, exclusive species may be known from outside our studied regions, and may occur elsewhere in South America (S3 Table). The term endemic is used to refer to those species that occur in a single mountain area or region within the study area and nowhere else.

### 2.4 Biogeographical patterns

Several analyses were employed to compare the relationships among the native herpetofaunas from different mountain systems within our study region. Introduced species were excluded from these analyses. The montane systems evaluated from east to west are: Tobago’s Main Ridge (TOB), Trinidad’s Northern Range (TRI), Paria Range (PR; including the Cerros Campeare and La Cerbatana), Turimiquire Massif (TUR), mountains of Isla de Margarita (IMA), Central Coastal Range (CCR), Sierra de San Luis (SSL), and Sierra Nevada de Santa Marta (SNSM) (Fig 1). To assess species richness in relation to geography, we plotted the number of species per area (km^2^) using a linear function with logarithmic scales (log-log Plot) in a Spatial Auto Regression (SAR) Model. The species-area relationships were done in PAST 4.0 [50]. The areas (km^2^) of the mountain systems were calculated using CalcMaps (www.calcmaps.com), taking as a minimum elevations of 200 m above sea level (S5 Table). A second area analysis was then done to calculate mid-altitude vegetation areas, such as those of humid montane forests, or evergreen forests (S6 Table), as well as the areas of forests that develop in the highest regions or sectors of mountain systems using Geographic Information System software in ESRI ArcGIS® (S5 Table). We then applied Simpson’s Index [51] to compare species compositions between different faunal groups of northern South America’s montane systems. A Simpson similarity index coefficient greater than 66.66% considers shared species between the montane systems, indicates a similar faunal group [51].

A multivariate analysis (classification and ordination methods) was next used to distinguish and compare the species composition and to recognize relationships between different faunal groups of the montane systems of northern Venezuela, Trinidad’s Northern Range, Tobago’s Main Ridge and the Sierra Nevada de Santa Marta, Colombia [52]. These methods are useful for discerning biogeographic patterns in amphibians and reptiles [53–56]. Cluster analyses (a classification method) were applied to amphibians and reptile matrices following a UPGMA algorithm and Jaccard similarity coefficient [57]. The Jaccard index coefficient considers shared species between the montane systems and the uniqueness (endemism) of species in each montane system, while cophenetic correlations tested natural groupings in the data [51,57–59]. Additionally, to contrast with multivariate classification, an ordination analysis (nonmetric multidimensional scaling analysis: MDS) was done using the Jaccard coefficient. All multivariate analyses were developed in PAST 4.0 [50].

### 2.5 Species biodiversity at mountain delimitation of 200 m

In our analyses, all species included in this study have a lowermost altitudinal distribution extent of approximately 200 m asl. Also, our analysis attempted to consider only species from mountainous habitats associated with forest surrounded by drier areas. However, this last condition is not met equally throughout the study region. For example, the area surrounding Sierra de San Luis is extremely dry, compared with the extremely humid region found in the northern versant of the Paria Range, with its abrupt slopes and humid forest extending to the coast. Therefore, using the approximate 200 m asl mark allowed us to include all species that inhabit low altitude mountain forests, including the highest points (e.g. 500 m asl), and exclude shoreline or lowland species (S3 Table). An example is Beebe´s Toad (*Rhinella beebei*); although excluded from our analysis, this species inhabits most of the ecosystems from northern Venezuela, including Isla de Margarita and Trinidad, but is found primarily at elevations under 200 m asl. The opposite occurs with the turtle *Kinosternon scorpioides* and the lizard *Anolis onca*; both species occur mostly near lowland water bodies and in xeric ecosystems near the coastal line, however, museum records support their presence at 300 m asl within the study area.

### 2.6 Species biodiversity at mid mountain altitudes in relation to vegetation

A second analysis of species-area relationships with species that only inhabit the humid montane forests was performed to assess association to mid-altitude montane habitats (S6 Table). For this analysis, we consider only those species present in montane forests such as semideciduous and cloud forests, including the low stratum vegetation found on some of the summits in the study region (e.g. those found on the Turimiquire Massif and Cerro El Copey on Isla de Margarita). The altitude at which these types of forests occur varies among the different montane systems and according to their slopes. To determine the lower altitude where these montane forests begin, we followed the literature (S6 Table). The second analysis resulted in much-reduced areas (km^2^) and occurrances on multiple separated peaks (polygons) on some of the summits (S5 Table).

### 2.7 Molecular analyses for species identification

Cryptic or juvenile species of reptiles and amphibians from the surveyed areas (La Cerbatana, Campeare, and Paria Range) were next studied using DNA analysis. Whole genomic DNA was extracted from tissue samples using the commercial DNA extraction kit Dneasy Tissue Kit (Qiagen, Hilden, Germany) following the manufacturers’ instructions. The targeted mitochondrial gene fragment was the 16S rDNA. The primers were: 16S rDNA: 16SL 5’-GCCTGTTTATCAAAAACAT-3’, 16SH 5’- CCGGTCTGAACTCAGATCACGT- 3’ [60]. All amplified sequences were approximately 510 base pairs long. Sequences were checked by eye, edited, and aligned using the program Sequencer v.4.9 and Seaview v.4.2.12 [61]. All sequences generated for this study are deposited in GenBank (S2 Table). Blast searches were conducted in GenBank for sequence identification. *P-*uncorrected distances were computed in MEGA [62].

## 3 Results

### 3.1 Species biodiversity at mountain delimitation of 200 m

Fieldwork, museum specimens, and literature reviews (S2 Text) suggest that 294 native species of amphibians (112 species) and reptiles (182 species) occur in the SNSM, and the mountain range complex of northern Venezuela (including IMA and TRI-TOB) (Tables 1 and S3, for a list of undescribed species see S3 Text). Forty-five percent of the species (131 taxa: 79 amphibians and 52 reptiles) are endemic or exclusive to the different sub-regions (Tables 1 and S3). The CCR had the highest endemism level with 149 native species (52 amphibians and 96 reptiles), with 32% of species (47 species, 31 amphibians, and 16 reptiles) only found there. The SNSM is the region with the next highest percentage of endemism: of the 117 reported native species, 36 (31%) are exclusive to this system.

**Table 1:** Total number of native species, endemic and introduced amphibians (top) and reptiles (bottom) in the studied region.

**Amphibians**	**SNSM**	**SSL**	**CCR**	**IMA**	**TUR**	**PR**	**TRI**	**TOB**	**Total**
**Total Species**	38	14	53	4	17	26	15	15	113
**Native Species**	38	14	52	4	16	26	15	15	112
**Endemic (%)**	24 (63)	3 (21)	31 (61)	0 (0)	4 (25)	12 (46)	1 (7)	4 (27)	79 (70)
**Introduced Species**	0	0	1	0	1	0	0	0	1
**Reptiles**	**SNSM**	**SSL**	**CCR**	**IMA**	**TUR**	**PR**	**TRI**	**TOB**	**Total**
**Total Species**	81	32	102	41	56	50	59	39	193
**Native Species**	79	32	96	39	54	49	56	36	182
**Endemic (%)**	12 (15)	0 (0)	16 (17)	4 (10)	4 (7)	5 (10)	6 (11)	5 (14)	52 (29)
**Introduced Species**	2	0	6	2	2	1	3	3	11

SNSM (Sierra Nevada de Santa Marta, Colombia), SSL (Sierra de San Luis), CCR (Central Coastal Range), IMA (Isla de Margarita), TUR (Turimiquire Massif), PR (Paria Range), TRI (island of Trinidad), TOB (island of Tobago).

The CCR in northern Venezuela harbors the highest number of native amphibians and reptiles (n = 148) and is the largest in area (30,190 km^2^) of all studied ranges; it is also the most well-studied area in Venezuela. In the Paria Region (PR), the Paria Range and associated mountains (that we herein assign to the PR), have a total of 75 native species, 26 amphibians and 49 reptiles, with a high percentage of endemism (23%, 12 amphibians and 5 reptiles) (Tables 1 and S3). In detail, the Cerro La Cerbatana has 19 species, followed by Cerro Campeare with 16 species, and the Paria Range with 72 species. Despite the 90 km separation created by the Unare depression between the eastern and the CCR, and a distance of 340 km separating the SNSM (Colombia) from the Venezuelan portion of this mountain system, the overall high number of shared species in two or more mountain systems (n = 118) among regions suggests a similar ancestral origin of species in the area or argues for efficient dispersal. Among the shared species, 92 (78%) are reptiles and 26 (22%) amphibians (S3 Table). This is perhaps explained by the greater relative dispersal capability of reptiles.

### 3.2 Species biodiversity delimitation following mid-altitude vegetation

Delimiting species richness to mid-elevations (montane forest), resulted in a much reduced species data matrix with a poorer representation of endemics or taxa associated only to this habitat, as present in the three islands. The less homogeneous topography at high elevations resulted in some mountain systems partitioning into separate peaks with each broad mountain top consisting of a few different polygons of varying size. Major differences compared to those using the 200 m altitude cut-off were found in Trinidad and CCR, each with 17 and 18 polygons, respectively (S5 Table). Using this approach, we find that a total of 125 exclusive or restricted species, 66 amphibians and 59 reptiles, occur in the eight mountain systems studied (Tables 2 and S4). For the amphibians, the highest number and percentage of exclusive species are present in the CCR (27 species: 41%) and SNSM, (21 species: 30%), followed by the PR (12 species: 18%). This species richness pattern in the western mountains is also observed in reptiles (CCR: 25 species, 31% and SNSM: 16 species, 20%), however, a decrease in species numbers is observed in PR and TUR (11 species: 14% each) (Table 2). It is important to point out that a low richness of restricted species is present in the montane forests of the systems’ islands (IMA, TRI and TOB), with no amphibian species on IMA and only one species in TRI (*Phytotriades auratus*) and TOB (*Pristimantis turpinorum*). This pattern is repeated with reptile richness, with only 3–4 species, and was also found in the continental mountain system of the SSL, with six reptiles and three amphibians.

**Table 2:** Total number of exclusive and restricted species of amphibians and reptiles to humid or evergreen mountain forests in the studied region.

**Amphibians**	**SNSM**	**SSL**	**CCR**	**IMA**	**TUR**	**PR**	**TRI**	**TOB**	**Total**
**Total Exclusive Species**	21	3	27	0	4	12	1	1	66
**(%)**	(30)	(4)	(41)	(0)	(6)	(18)	(1)	(1)	
**Reptiles**	**SNSM**	**SSL**	**CCR**	**IMA**	**TUR**	**PR**	**TRI**	**TOB**	**Total**
**Total Exclusive Species**	16	6	25	3	11	11	4	4	59
**(%)**	(20)	(7)	(31)	(4)	(14)	(14)	(5)	(5)	

SNSM (Sierra Nevada de Santa Marta, Colombia), SSL (Sierra de San Luis), CCR (Central Coastal Range), IMA (Isla de Margarita), TUR (Turimiquire Massif), PR (Paria Range), TRI (island of Trinidad), TOB (island of Tobago).

### 3.3 Biogeographic data analyses at mountain delimitation from 200 m

**Fig 3:**
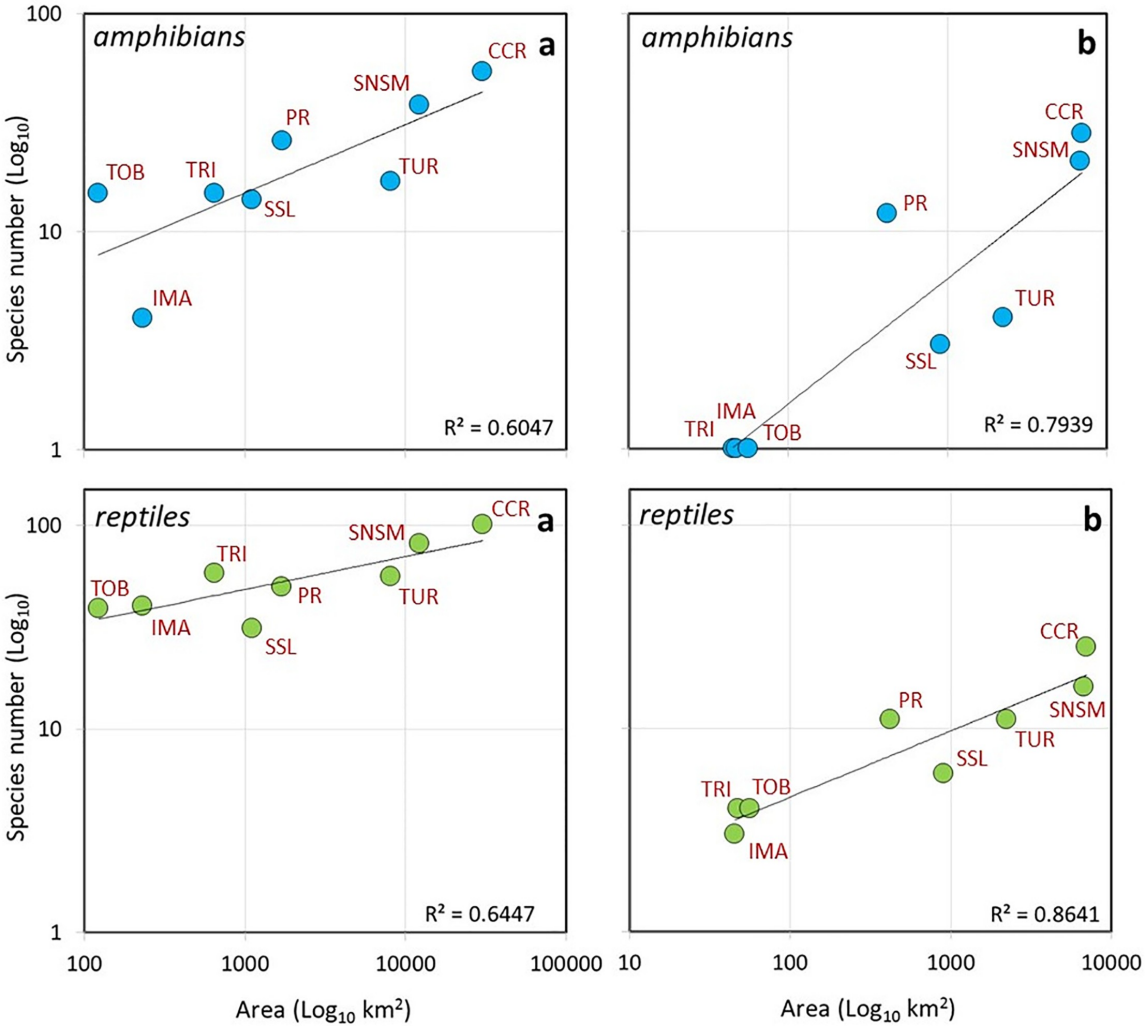
Species-area relationships using the linear function for (a) amphibians (blue circles) and (b) reptiles (green circles). The two graphs on the left (a) are from species-area calculated above 200 m in elevation and the two right graphs labelled (b) are from species-area calculated from mid mountain vegetation. SNSM (Sierra Nevada de Santa Marta, Colombia), SSL (Sierra de San Luis), CCR (Central Coastal Range), IMA (Isla de Margarita), TUR (Turimiquire Massif), PR (Paria Range), TRI (island of Trinidad), TOB (island of Tobago).

The species-area relationship showed expected trends, but the deviation of the montane complex above or below the predicted line suggests areas with higher (TRI, PR, CCR and SNSM) and lower (IMA, TUR, TOB) species numbers in relation to those predicted with species-area relationships model (Spatial Auto Regression (SAR) Model) [63]) (Fig 3A). This is also explained by the humidity differences between the mountains and the lowlands: wetter regions had a greater number of species than drier ones. There was a strong correlation between both reptiles and amphibian richness and area, with CCR and SNSM recovering the highest correlations for both groups. Despite the considerably smaller areas of the PR and TRI they recovered higher species richness than TOB, SSL, IMA and TUR (Fig 3A).

Our data revealed a high number of widely distributed species shared among all systems (118 species = 40% of the total native species). A Simpson similarity coefficient, was significant between the amphibian fauna of the montane systems of the CCR and SSL (S = 78%), and IMA (S = 100%), between IMA and the TUR (S = 75%), as well as between TRI and the PR and between TRI and TOB (S = 73%, respectively) (Table 3). For reptiles, this index showed significant associations between the CCR and SSL (S = 97%), IMA (S = 84%), the TUR (S = 73%), with the PR (S = 66%) and with TOB (S = 67%). In the same way, the TUR showed a high similarity with the IMA and with the PR (S = 68% and 66%, respectively), as did the island of TRI with the PR (S = 66%) and TRI with TOB (S = 81%) (Table 3).

**Table 3:** Matrix of Simpson´s similarity index of amphibians (top) and reptile (bottom) between all regions.

	SNMS	SSL	CCR	IMA	TUR	PR	TRI	TOB
**SNMS**	-	0.21	0.23	0.5	0.5	0.23	0.4	0.4
**SSL**	0.5	-	0.78	0.5	0.28	0.28	0.28	0.21
**CCR**	0.49	0.97	-	1.00	0.62	0.30	0.53	0.46
**IMA**	0.55	0.38	0.84	-	0.75	0.5	0.5	0.5
**TUR**	0.43	0.41	0.73	0.68	-	0.62	0.53	0.46
**PR**	0.4	0.41	0.66	0.5	0.66	-	0.73	0.53
**TRI**	0.35	0.41	0.6	0.5	0.62	0.66	-	0.73
**TOB**	0.44	0.31	0.67	0.47	0.61	0.56	0.81	-

SNSM (Sierra Nevada de Santa Marta, Colombia), SSL (Sierra de San Luis), CCR (Central Coastal Range), IMA (Isla de Margarita), TUR (Turimiquire Massif), PR (Paria Range), TRI (island of Trinidad), TOB (island of Tobago).

**Fig 4:**
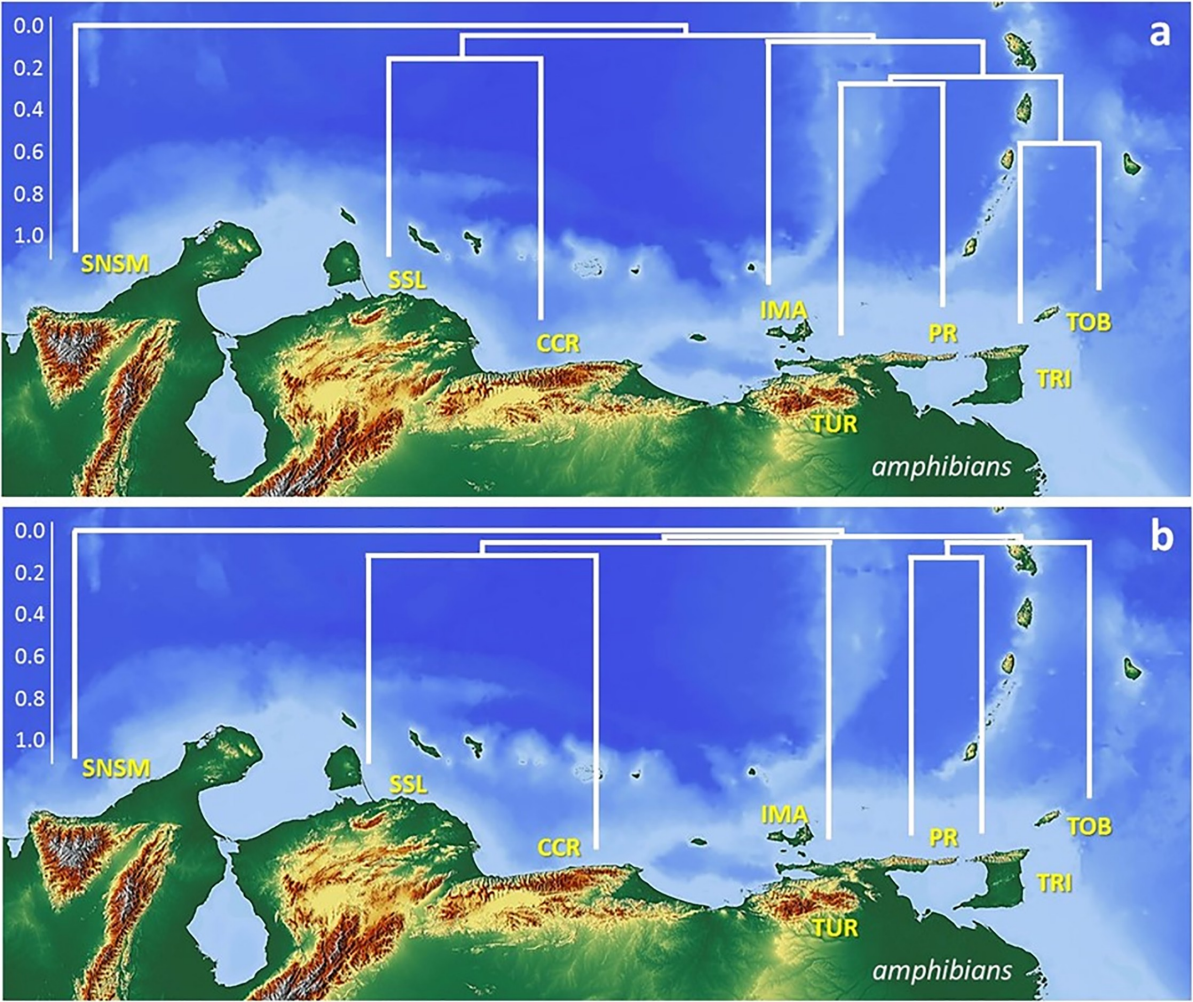
Distribution patterns of amphibians in the different orographic regions. Cluster classification for amphibians (a) found above 200 m in elevation and (b) found exclusively at mid altitude mountain vegetation, based on the UPGMA algorithm and the Jaccard coefficient (cophenetic correlation r = 0.95 and r = 0.92). SNSM (Sierra Nevada de Santa Marta, Colombia), SSL (Sierra de San Luis), CCR (Central Coastal Range), IMA (Isla de Margarita), TUR (Turimiquire Massif), PR (Paria Range), TRI (island of Trinidad), TOB (island of Tobago). Map build with GIS Cloud Apps (www.giscloud.com). Reprinted from Relief Free Map under a CC BY license, with permission from the Open Data Commons Open Database License (ODbL) by the OpenStreetMap Foundation (OSMF), original copyright OpenStreetMap^®^.

**Fig 5:**
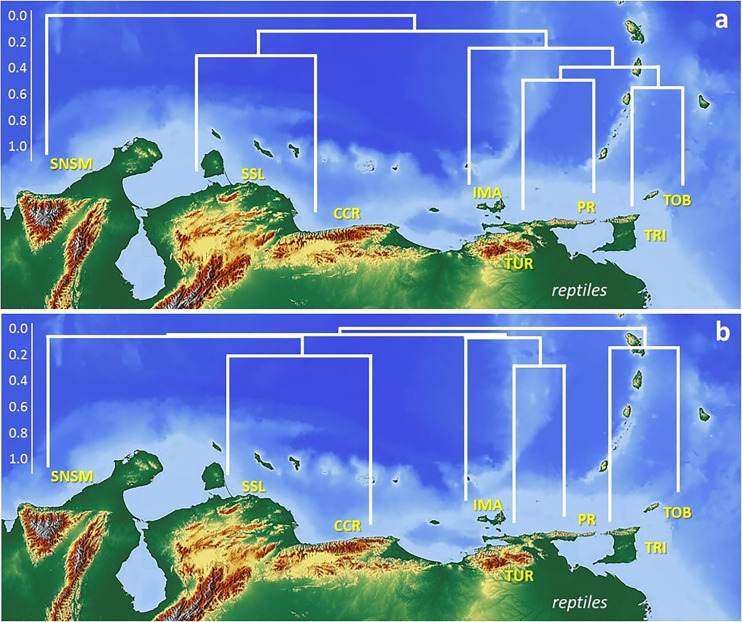
Distribution patterns of reptiles in the different orographic regions. Cluster classification for reptiles (a) found above 200 m in elevation and (b) found exclusively at mid altitude mountain vegetation, based on the UPGMA algorithm and the Jaccard coefficient (cophenetic correlation r = 0.81, and r = 0.84). SNSM (Sierra Nevada de Santa Marta, Colombia), SSL (Sierra de San Luis), CCR (Central Coastal Range), IMA (Isla de Margarita), TUR (Turimiquire Massif), PR (Paria Range), TRI (island of Trinidad), TOB (island of Tobago). Map build with GIS Cloud Apps (www.giscloud.com). Reprinted from Relief Free Map under a CC BY license, with permission from the Open Data Commons Open Database License (ODbL) by the OpenStreetMap Foundation (OSMF), original copyright OpenStreetMap^®^.

**Fig 6:**
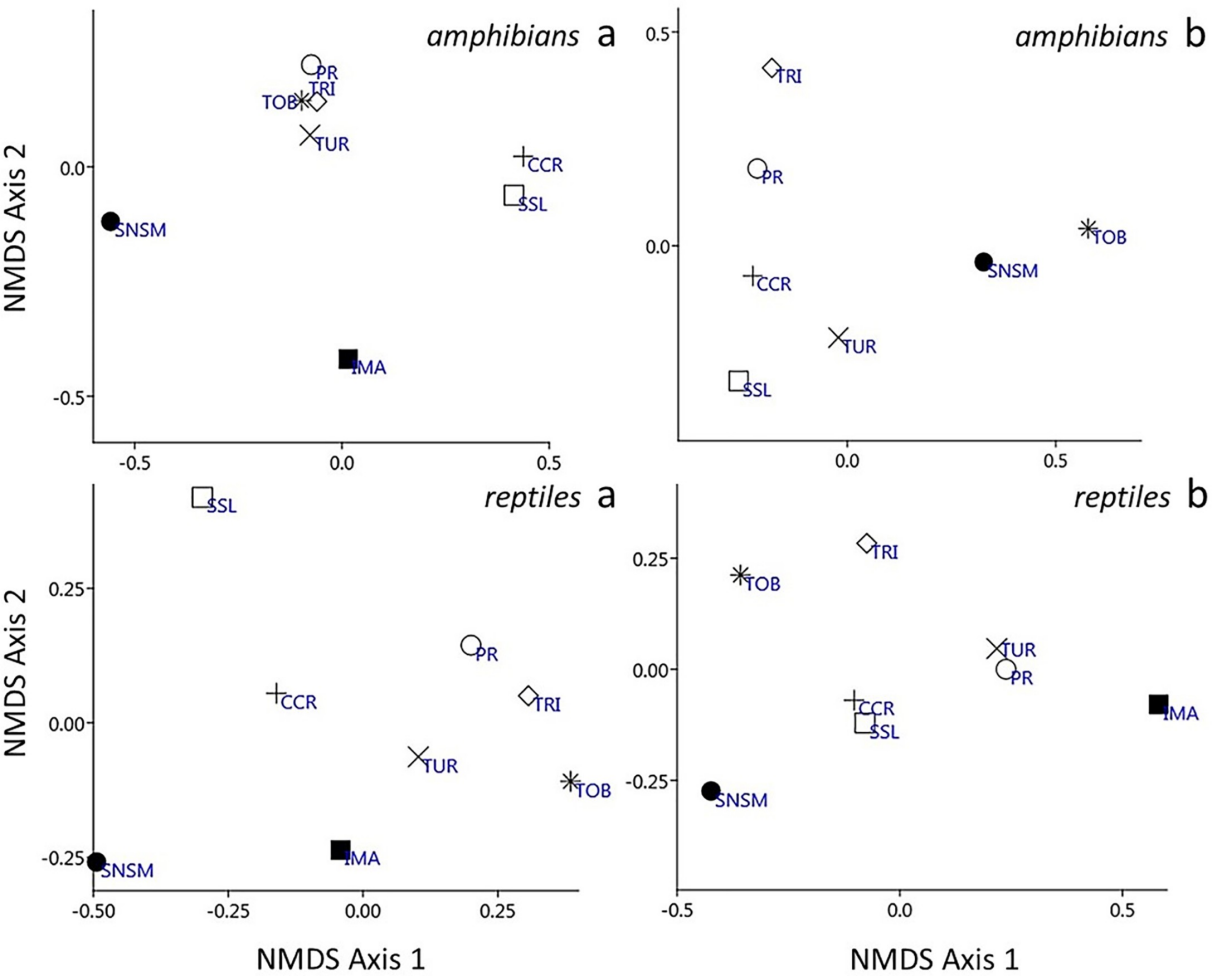
Metric multidimensional ordination and scaling (NMDS) based on the UPGMA algorithm and the Jaccard coefficient for amphibians and reptiles. The two graphs on the left are (a) amphibians (top) and reptiles (bottom) found above 200 m in elevation and the two graphs at the right labelled (b) are amphibians (top) and reptiles (bottom) found exclusively associated to mountain vegetation. SNSM (Sierra Nevada de Santa Marta, Colombia), SSL (Sierra de San Luis), CCR (Central Coastal Range), IMA (Isla de Margarita), TUR (Turimiquire Massif), PR (Paria Range), TRI (island of Trinidad), TOB (island of Tobago).

The results from the cluster classification analyses (Jaccard coefficient) for both amphibians and reptiles (Figs 4A and 5A) revealed a good adjustment of the data with high cophenetic correlation for both groups (r = 0.95, Fig 4A and r = 0.81, Fig 5A) with under 20% similarity between both groups. The classification and ordination sorting followed the mountainous geography disposition. The relationships between the amphibians and reptiles were differentiated in two large groups: one corresponded to fauna that inhabits the occidental and central mountain ranges (SNSM, SSL, CCR) and the other to a group strictly associated with the oriental mountain ranges (IMA, TUR, PR, TRI, TOB). This group showed a closer association than the former group. At the second level (~ 25% similarity), two groups were detected: SSL, CCR, and IMA, TUR, PR, TRI, TOB. The multidimensional ordination recovered a similar pattern congruent with the geographic mountain ranges, moderately low in amphibians (stress 0.0135; axis 1: 0.368; axis 2: 0.026) and reptiles (stress 0.123; axis 1: 0.586; axis 2: 0.254) (Fig 6A). In all cases, the similarity in the sorting by geographic range disposition corroborates the natural arrangement of the data.

### 3.4 Biogeographic data analyses at mountain system delimitation following mid-altitude vegetation

Here, the relationships between species richness and the calculated areas suggest that PR has a higher number of species than predicted by the model, contrary to TUR and SSL, and to a lesser degree IMA, TRI and TOB (Fig 3B). The fit to the model is the consequence of very large areas being compared to very small ones and important differences in species richness. Likewise, in this case, the model’s high slope suggests a rapid increase in the number of species in response to increases in area (Fig 3B).

Only a small number of species share distributions between any two mountain systems. Four amphibian species (5% of the total species) share their distribution, two between nearby mountain systems: *Flectonotus pygmaeus* in SSL and CCR, *Phytotriades auratus* in PR and TRI, and for one case, between two more distant mountain systems: *Strabomantis biporcatus* in CCR and PR (S5 Table). The situation above may be better characterized and assessed when we apply the Simpson similarity coefficient (Table 4); in this case, no true associations are formed between the amphibian fauna. There are slight differences regarding the reptile fauna since only 14 species (24%) of the 59 species share their distribution between two and four mountain systems. However, only in one case, true associations are formed. The six species registered in the forests of the Sierra de San Luis (SSL), are also found in the Central Coastal Range (CCR), showing a 100% Simpson similarity coefficient (Table 4). In all remaining areas, the values of this coefficient do not exceed 50%, and only in a specific case, where one of the four species present in TRI (*Helminthophis flavoterminatus*) are also present in the CCR (Tables 2 and S4).

**Table 4:** Matrix of Simpson´s similarity index of amphibians (top) and reptile (bottom) between all regions.

	SNMS	SSL	CCR	IMA	TUR	PR	TRI	TOB
**SNMS**	-	0.00	0.00	0.00	0.00	0.00	0.00	0.00
**SSL**	0.16	-	0.33	0.00	0.00	0.00	0.00	0.00
**CCR**	0.06	1.00	-	0.00	0.25	0.08	0.00	0.00
**IMA**	0.00	0.00	0,00	-	0.00	0.00	0.00	0.00
**TUR**	0.00	0.33	0.36	0.00	-	0.00	0.00	0.00
**PR**	0.00	0.33	0.27	0.33	0.45	-	0.00	0.00
**TRI**	0.00	0.00	0.5	0.00	0.25	0.00	-	0.00
**TOB**	0.00	0.00	0.25	0.00	0.00	0.00	0.25	-

Species restricted to humid or evergreen montain forests in the studied region. SNSM (Sierra Nevada de Santa Marta, Colombia), SSL (Sierra de San Luis), CCR (Central Coastal Range), IMA (Isla de Margarita), TUR (Turimiquire Massif), PR (Paria Range), TRI (island of Trinidad), TOB (island of Tobago).

In general, the biogeographic relationships calculated through the classification and ordination analyses are very low for both the fauna and montane systems. Despite the high cophenetic correlation we derive from the classification analyses (r = 0.92 Fig 4B and r = 0.84 Fig 5B), the observed relationships are extremely low. Amphibians recovered a more disorderly arrangement of the groups, associated with low species richness detected in certain sites (TRI-TOB), and lack of species (IMA). The SNSM recovered the lowest relationship to the remaining montane systems (Jaccard coefficient, <10%). Two groups are recovered (<20%); a western group (SSL, CCR), including the eastern TUR system, and an eastern group (PR, TRI) differentiated from TOB (Fig 4B). The reptile fauna recovered three groups; a first western group with SNSM associated with a low coefficient (<20%) to SSL and CCR, which are more related to each other (30%), and two eastern groups (TUR, PR 30%, each with an association to IMA (<20%), and (TRI, TOB, ca. 20%). There is a stronger association of eastern fauna (Fig 5B).

The ordination (non-metric multidimensional scaling) analysis corroborates the results detected in the previous classification (Figs 4B and 5B) with minimal relationships between faunal groups. Two groups, a western (CCR-SSL) and eastern (TUR-PR) were recovered for the reptile fauna. However, unlike the results found with the analysis of faunas studied from 200 m asl, in this case, the arrangements were more unstable with stress dramatically increased (amphibian stress 0.2722; axis 1: 0.195; axis 2: 0.028 and reptiles: stress 0.2629; axis 1: 0.291; axis 2: 0.207) (Fig 6B). This can be explained by the comparison of areas very different in size (45 to 7.026 km^2^ S5 Table) and the much lower numbers of species (amphibians: 1 to 27, reptiles: 3 to 25: Table 2).

### 3.5 The faunas at Cerro La Cerbatana and Cerro Campeare

Our surveys demonstrate that both mountains Campeare and La Cerbatana are in fact biological extensions of Paria Range and that they likely represent relict remnants of forests that occupied this entire region during interglacial periods. The following species are noted for Cerro La Cerbatana and Cerro Campeare (for genetic data see S2 Table and for undescribed species S3 Text): *Mannophryne venezuelensis*, *Rhinella marina*, *Flectonotus fitzgeraldi*, *Pristimantis nubisilva*, *Gonatodes ceciliae*, *Gonatodes* sp, *Thecadactylus rapicauda*, *Anolis planiceps*, *Plica caribeana*, *Ameiva atrigullaris*, *Cnemidophorus lemniscatus*, *Ninia atrata*, and *Tantilla melanocephala*. Additionally, *Phyllomedusa trinitatis*, *Boana xerophylla* and *Hemidactyus mabouia* occur in Cerro Campeare. Interestingly, a specimen of *Ninia atrata* from Campeare (MBLUZ 1426) has three labials contacting the primary temporal scale, suggesting that it may be *Ninia franciscoi*, a species described based on a single male specimen from northern Trinidad [64]. However, a DNA analysis of the specimen showed that this individual belongs to the species *Ninia atrata* from the Central Coastal Range. Finally, *Leptodactylus* sp., *Gonatodes vittatus*, *Oreosaurus rhodogaster*, *Copeoglossum aurae*, *Phrynonax polylepis* and *Bothrops venezulensis* were found in La Cerbatana but not in Campeare.

## 4 Discussion

We recovered contrasting reptile and amphibian species diversity attributed to two different altitude delimitations. Species diversity increased when using a cut-off point at 200 m in elevation in all montane systems (294 species) in comparison to fauna associated strictly with mid-altitude montane areas. This was true for the relationship to vegetation cover as well as climatic conditions (125 species). The more than two-fold reptile and amphibian species biodiversity recorded above 200 m, distributed from the lower mountain areas to the summits, suggests a gradual distribution throughout the elevation gradient. This distribution offers weak environmental barriers, habitat ecotones, or slopes. Similar patterns of shifts in montane situations during the Quaternary are well documented [43]. Montane species increase their distribution along elevation gradients in response to cooling and warming temperatures [65]; however, in relatively low altitude homogenous tropical climate montane environments, distribution shifts are more likely to result in intermixing of species and populations throughout the altitudinal gradient [43] rather than isolation of species in specific areas. Except for the high elevations of SNSM, the remaining mountain peaks are of relatively low altitude; and may not be sufficient for large (with the exception of CCR), stable cloud forest habitats to accumulate higher endemism or numbers of species specialized for such habitats.

This effect is more accentuated in the islands’ lower altitude montane systems, as sea-level fluctuations were essential in species diversity and mixing of populations [43,66] and similarly by lower altitude ranges. During interglacials, high sea levels reduced the area of islands and increased their isolation. In contrast, during glacial periods, terrain expanded and former isolated islands were connected to other islands or the mainland. Continental shelf islands or fragmented islands [67] such as Trinidad and Margarita, and even Tobago depending on the time under consideration, connected via land bridges to the mainland [46,47,68], and this reflect on the lower number of endemics when faunas interconnect [69]. Therefore, past surface area and inter-island connections play an important role in explaining present-day richness and genetic diversity. Looking at only the current snapshot is insufficient to fully understand the observed patterns; incorporating more geology into analyses like ours will be required to do that [3].

Interestingly, the biodiversity at higher elevations in the PR exceeded our model expectations. However, peninsulas are known to have the capacity to harbor a more significant number of reptile and amphibian species. They represent distributional limits for species dispersal, especially throughout changing climatic conditions such as glacial and interglacial periods [70,71]. Furthermore, the PR has likely been colonized from both southern and northern regions, increasing its biodiversity. Some evidence for colonization routes may derive from the low genetic divergence of species between Guyana, French Guiana, and Trinidad (an extension of the PR) from eastern coastal regions, most likely through Pleistocene eustatic sea-level falls [72–74]. Similarly, genetic divergence of *Elachistocleis* from the southern Lago de Maracaibo basin, a region located between the Cordillera de Mérida and Serranía de Perijá (Western versant Cordillera de Mérida), show high genetic affinities to Trinidad *Elachistoclies* sp. (unpublished data).

Overall, the resulting differences in the altitude delimitation of species above 200 m suggest a natural or realistic model for representing biodiversity in the montane systems, with similar biodiversity indexes following similar topographic dispositions (Fig 4A and 4B). Unlike the results found with the analysis of faunas studied from 200 m asl, arrangements of higher elevation taxa were more unstable, with lower numbers of species shared between areas (n = 17: 3 amphibian and 14 reptiles, S4 Table) and a minimal association between areas (Fig 5A and 5B). It is therefore apparent that faunal patterns at higher elevations represent more exclusive taxa which are less useful to assess biogeographic relationships in the region. In accordance, we next focus our discussion mainly on the first analyses of species biodiversity from 200 m in elevation to unravel those biogeographic patterns and associations.

We recorded a total of 294 species (112 amphibians and 182 reptiles) present throughout the region. As discussed in the Results section, the CCR and SMSM recovered the highest numbers of endemic species. The high number of Trinidad´s herpetofauna compared to the other two other islands in our analyses, TOB and the IMA, is evidence of its prolonged connections to the PR [41] and its larger size. Interestingly SSL recovered low species diversity in comparison to the proximal CCR (Fig 1). The richness of the PR is related to some degree with the amphibian and reptiles of other mountain systems of northern Venezuela, as well as those from the mountains of IMA, TRI, and TOB.

Islands such as Trinidad and Margarita, which were previously part of a common landmass that encompassed much of northern Venezuela, from which they became isolated, are important to understand the patterns of endemism [68]. Fragment islands such as these, which initially contained continuous terrestrial ecosystems before marine inundation, started out with species sets that are more representative of the regional pool [67] and therefore conform better to the Paria Range biota than to those of the other western mountain systems (Fig 4A and 4B). Furthermore, isolation is highly dependent on the bathymetry between islands and archipelago configurations [75–77]; the low marine depths between Trinidad and Margarita to the mainland (under 100 m) suggest prolonged connections [46,78]. This, in turn, affects how and how fast endemism develops, and whether isolation is strong enough and persists long enough. Therefore, these islands have mostly a subset of the regional species pool. The very high reptile and amphibian diversity of TRI and TOB follow patterns related to the PR peninsula effect. When these two systems were part of the end of the peninsula mountain system, they acculumated species biodiversity, and then remained isolated after their last connection to the peninsula.

Within the Paria Peninsula, the most studied area has been its mountains, where research began in the 1960s [79–81]. Other systems in the Paria Range have been poorly studied, e.g., La Cerbatana and Campeare were studied here for the first time. However, all species recorded from Campeare and La Cerbatana are also found in the Paria Range, except for an undescribed frog (*Leptodactylus* sp.), apparently endemic to La Cerbatana. This seems to indicate that the three regions constitute a single biogeographical unit for the studied vertebrate clades. Similar findings have been supported by botanical data conducted in these three montane systems [40]. Herein we designate this unit as the Paria Range or Paria Region.

The high diversity of reptile species found throughout the study area reflects their dispersal capability and presence of habitat generalists when compared to the more restricted and ecologically constrained amphibians. Trinidad´s Northern Range shares the highest numbers of reptiles and amphibians with the PR (11 amphibians and 34 reptiles) followed by the CCR (8 amphibians and 35 reptiles) and TUR (8 amphibians and 35 reptiles). Tobago has the highest number of shared species of reptiles and amphibians with TRI (11 amphibians and 30 reptiles). These patterns suggest that all of these areas form a single biogeographic unit. Interestingly, the herpetofauna composition of the montane systems of Isla de Margarita does not follow similar expected biogeographical patterns. Margarita island shares a higher number of species (4 amphibians and 34 reptiles) with the CCR (240 km west) than with PR (75 km south east) sharing only 23 species (2 amphibians and 21 reptiles). However, at higher altitudes, a different pattern is observed with an overall more even reptile-amphibian ratio. The grouping of reptiles at higher altitudes in CCR-SSL and TUR-PR suggests likely connections and, or dispersal between these localities, possibly at glacial times (20 kya), that were not observed in the amphibian fauna. This likely reflects less specific adaptation to habitat by reptiles than amphibians, especially in cloud forest environments. Amphibians show a more restrictive distribution than reptiles do in the high elevations of this mountain system.

According to the Simpson´s similarity index, the PR does not form a faunistic group with TUR for amphibians (63%), but does for reptiles (66%), (Table 3). The highest Simpson´s similarities followed geographical proximity, with the CCR and SSL (amphibians 78% and reptiles 97%) and the montane range of IMA (amphibians 100% and reptiles 84%). The TUR and IMA recovered high similarity (75% amphibians, 68% reptiles). Trinidad and Tobago faunal similarity (73% amphibians and 81% reptiles) likely reflect prolonged periods of connections between these two neighboring islands during low sea level stands during the Pleistocene [46]. However, the similarity between TRI and the PR was equally high for amphibians (73%) but lower for reptiles (66%) when comparing TOB to TRI.

The Jaccard similarity index dendrogram clearly identified three groups for reptiles and amphibians, with minor intergroup relationships; an eastern group composed of TRI and TOB, and the PR including the TUR and the mountains of IMA. The western Venezuelan group is composed of the CCR and SSL and the Colombian SNSM. The groupings (TRI–TOB, PR–TUR) suggest similar biogeographic and phylogeographical scenarios in the region. Again, at higher elevations, and except for SSL and CCR (S = 100%), all other associations were weak, suggesting limiting mixing between regions and limited dispersal.

### 4.1 Biogeography

Within the last two decades, advances in molecular methodologies have greatly changed perspectives and understanding of the phylogeographic and biogeographic processes across our studied region [43,45,82]. Most of these studies have however focused on the eastern rather than the western region of northern Venezuela, especially around the PR, and the islands of TRI-TOB [43,45,73,82]. Such study bias results in part from the enticing evolution that the region’s unique habitats and topography (Fig 1), proximity to northern Venezuela, and the existence of well-dated Pliocene-Pleistocene land-bridge formations to the mainland play in the complex pattern of stepping stone colonization of the Caribbean [83–85]. Furthermore, TRI-TOB have served as refugia [43], as has the CCR, contributing to endemism in these areas [43,86]. The region’s exceptional evolutionary history and the use of molecular methods have also opened new insights into the tempo and mode of speciation in the region [87,88].

**Fig 7:**
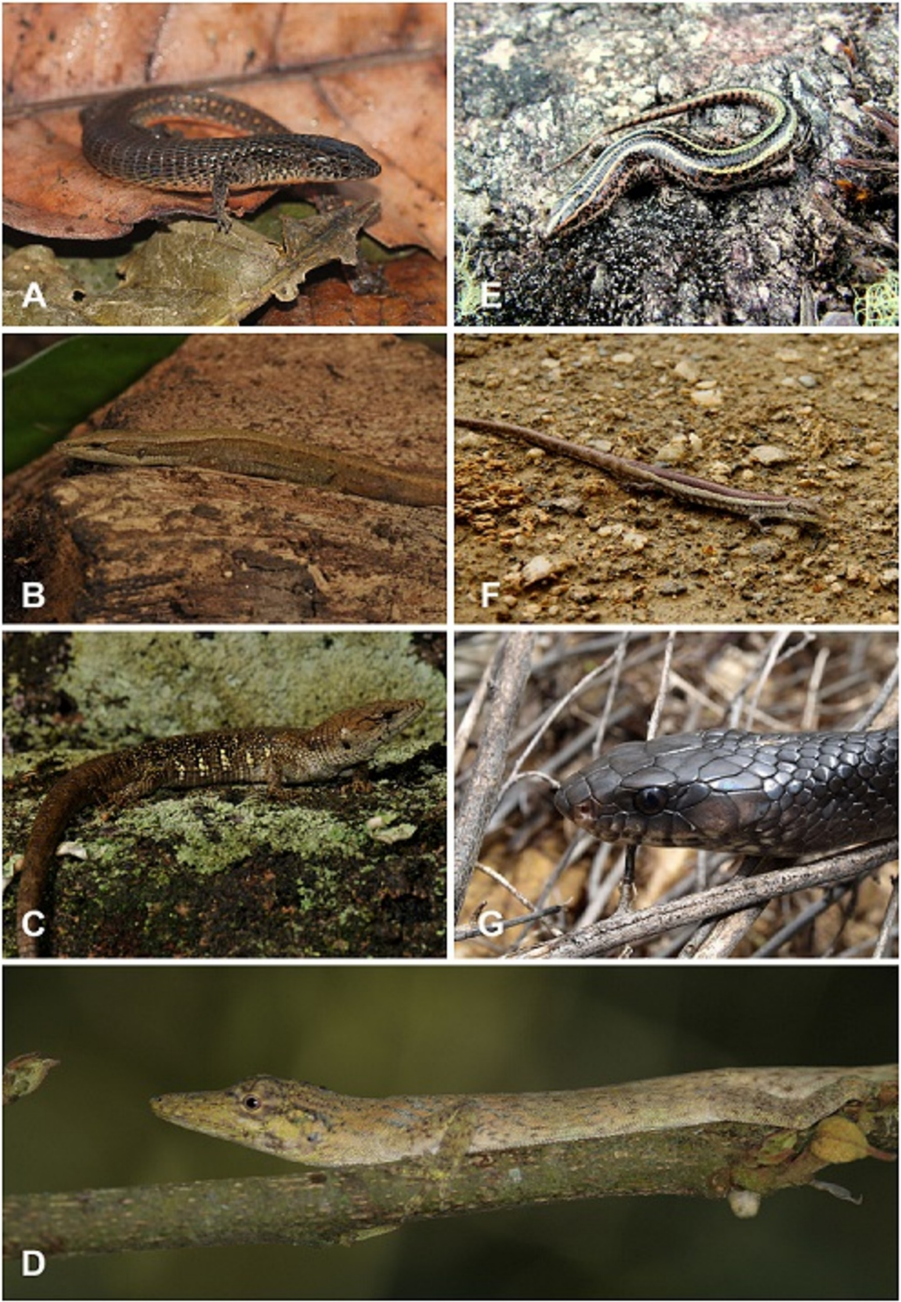
Reptiles found on Sierra de San Luis, Central Coastal Range, Turimiquire Massif and Isla de Margarita, Venezuela. A. Kugler´s Largescale Lizard *Ptychoglossus kugleri* (MBLUZ 1445). B, Sharp-snouted Sun Tegu *Euspondylus acutirostris* (EBRG 5857). C. The Spotted Anadia *Anadia marmorata*. D. The Twing Anole *Anolis tigrinus*. E. the Turimiquire Blue-Tailed Skink *Panopa croizati*. F. Female specimen of Steyer´s Anadia *Anadia steyeri* (MBLUZ 1314). G. The Margarita Cribo *Drymarchon margaritae*. Photo credits Helga Terzenbach (A, F), Luis A. Rodríguez J. (B-C), Rosario Castañeda (D), Gilson A. Rivas (E), Gonzalo Medina with cortesy of Maria Abarca (G).

**Fig 8:**
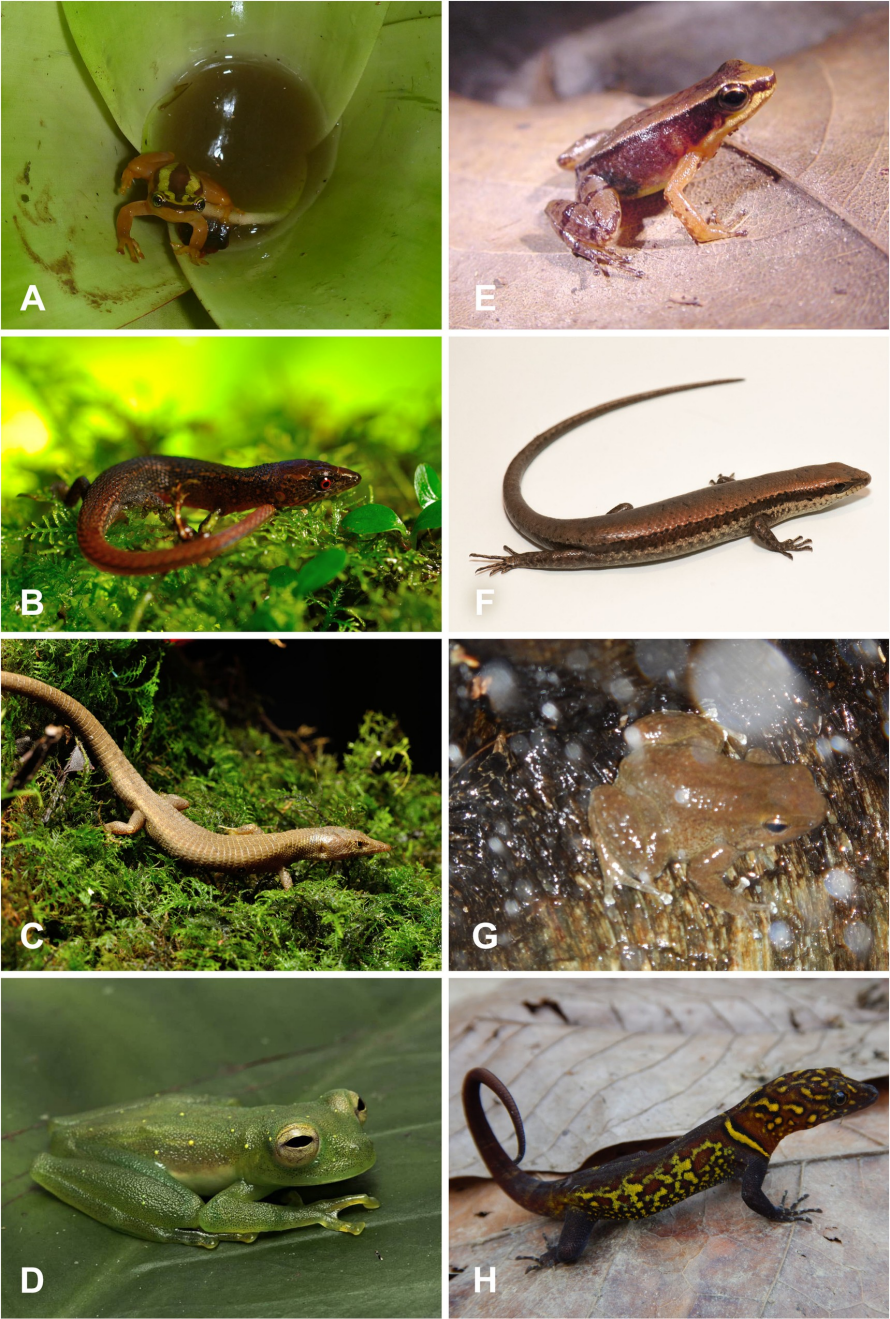
Amphibians and reptiles from the Paria Range in Venezuela. A. Individual of Golden tree frog *Phytotriades auratus* from Cerro Humo. B. Red bellied lizard *Oreosaurus rhodogaster*, described initially from the Peninsula de Paria. C. Sucre sun Teju lizard *Euspondylus monsfumus*. D. Castroviejo’s Glassfrog *Vitreorana castroviejoi*. E. The Caribbean nurse frog *Allobates caribe* (MHNLS 17498), is known only from three individuals (all females) collected on Cerro Humo (Paria Range). F. Greater windward skink *Copeoglossum aurae* (MBLUZ 1412). G. The Rivero´s nurse frog *Mannophryne riveroi*. H. The variegated gecko *Gonatodes ceciliae*. Photo credits Mayke De Freitas (A-C), Luis Merlo (D), Hinrich Kaiser (E), Luis A. Rodríguez J. (F), Gilson A. Rivas (G-H).

Steyermark [89] noted affinities of the flora of the Venezuelan CC, including the Paria Range and TUR, with those from the Amazonian and the Guiana Shield. Three species of reptiles from the eastern CC have their origin outside of northern Venezuela (Figs 7 and 8). Among them, the snake *Taeniophallus nebularis* from the highland of Paria forest is closely related to *T*. *nicagus* and *T*. *brevirostris* from the Amazonian/Guiana lowland [90]. The skink, *Panopa croizati* from the high peaks in the TUR is the sister species of *P*. *carvalhoi*, which inhabit the Brazilian Amazonia [91]. Similarly, the species of microteiid lizards of the genus *Oreosaurus* from northern Venezuela and TRI show affinities with *O*. *macdiarmidi* from the Venezuelan Guyana, as noted by Santiago-Pacheco et al. [92]. The endemism and composition of the herpetofauna in northeastern Venezuela have also been suggested to reflect geological events in the region [90–92] (Figs 7 and 8).

**Fig 9:**
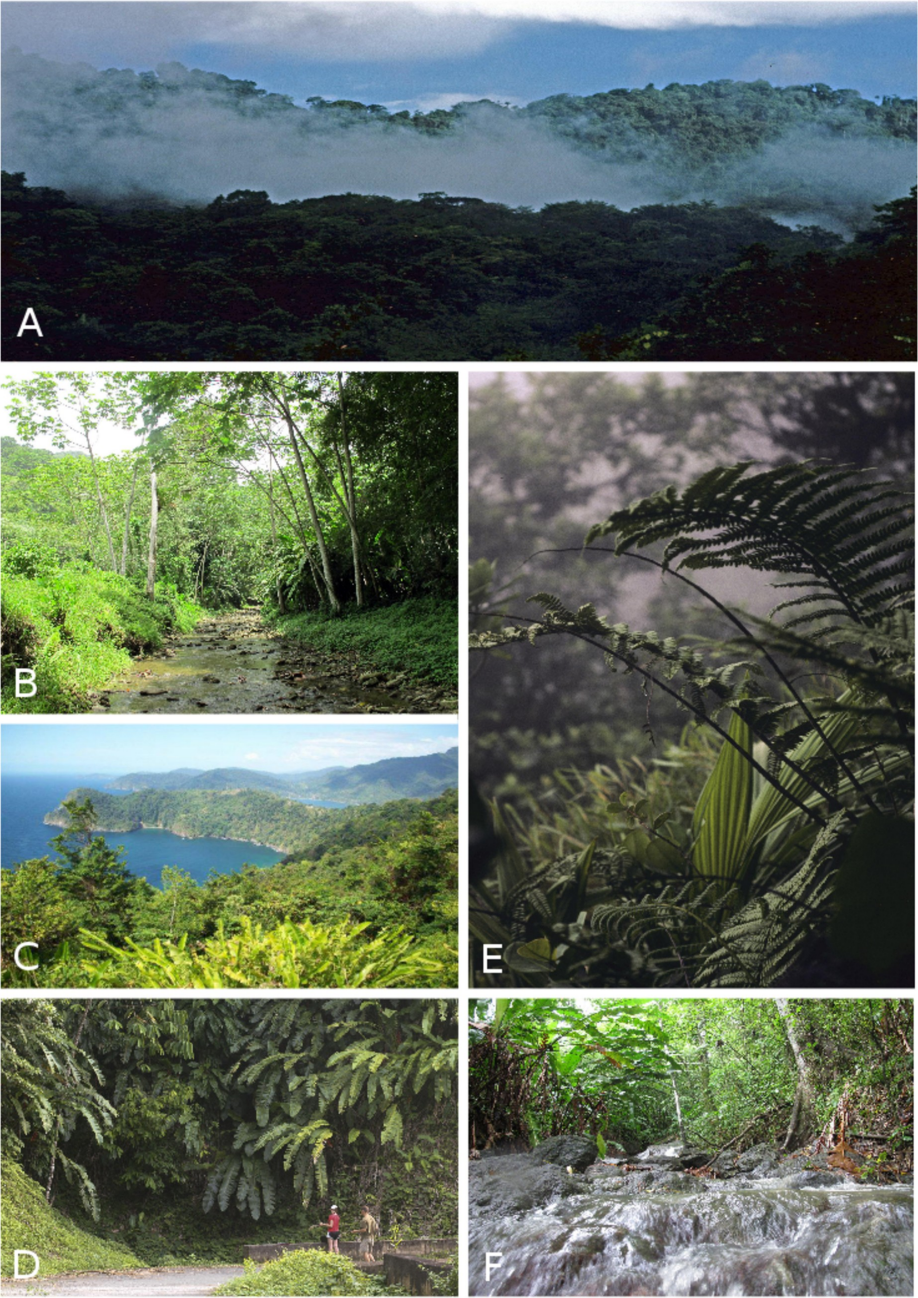
Habitats of Trinidad´s Northern Range and Tobago´s Main Ridge. A. Trinidad´s Northern Range holds most of the endemic and near endemic species known from the island; B. A stream in Tobago´s Main Ridge; C. A view of Trinidad´s coastal Northern Range to the West at Maracas Bay; D. A ravine on Tobago´s Main Ridge; E. Trinidad´s Northern Range El Tucuche cloud forest with elfin woodland; F. A stream in Tobago´s Main Ridge. Photo credits John Weber and John C Murphy.

**Fig 10:**
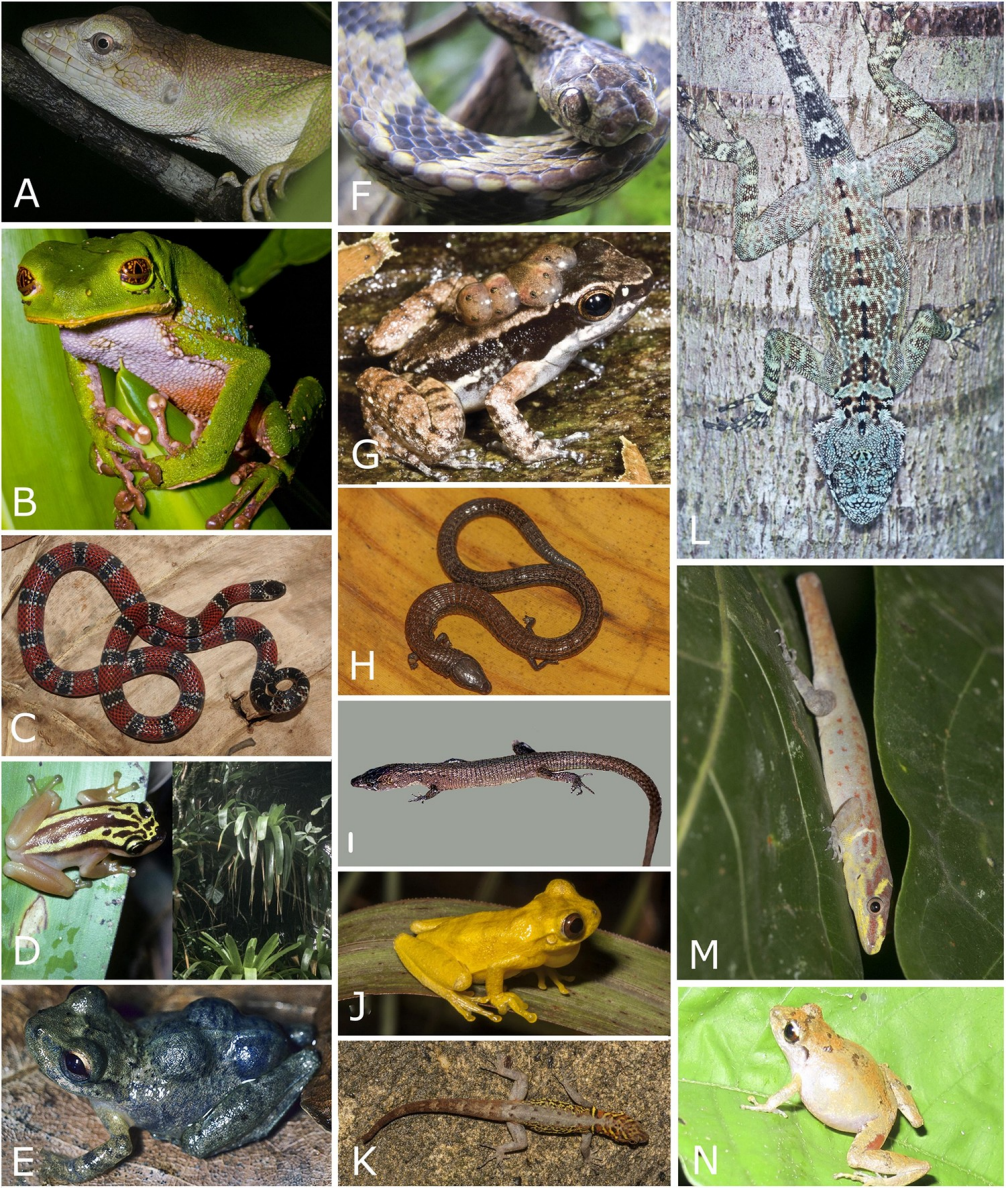
Some Trinidad amphibians and reptiles. A. The Venezuelan Bush Anole, *Polychrus auduboni*. B. Trinidad Leaf-nesting Frog, *Phyllomedusa trinitatis*. C. Trinidad Coral Snake, *Micrurus circinalis*. D. Golden Treefrog, *Phytotriades auratus*. E. Stimson’s Parrot Snake, *Leptophis stimsoni*. F. Trinidad Snail-eating Snake, *Dipsas trinitatus* (Trinidad endemic). G. Trinidad Stream Frog, *Mannophryne trinitatis* (a male transporting tadpoles endemic). H. Hex-scaled Bachia, *Bachia trinitatus*. I. Luminous Lizard, *Oreosaurus shrevei*. J. Minute Yellow Treefrog, *Dendropsophuscf minutus*, K. Variegated Gecko, *Gonatodes ceciliae*. L. Caribbean Tree Runner, *Plica caribeana*. M. The Trinidad Spot-nosed Gecko, *Gonatodes ferrugineus*. N. Urich’s Rain Frog, *Pristimantis urichi*. Photo credits John C Murphy (A-N).

The “continental” island of Trinidad (Fig 9) has six endemic species (1 frog, 2 lizards, and 3 snakes) (Fig 10), mostly found in the Northern Range [47]. Trinidad harbors one more endemic species (though see [47]), the Trinidad snail-eating snake (*Dipsas trinitatis*), but this species was not considered here because it inhabits lowlands below 200 m and outside the Northern Range. The smaller number of endemic species in Trinidad than that for the much smaller island of Tobago is further evidence of its past geology. Trinidad’s Northern Range was a connected northeastern extension of the Paria Range until the Pliocene when subsidence associated with the Gulf of Paria (Boca del Dragón) separated these two land-masses [93–96]. Prolonged land bridge connections thereafter, at low sea level stands in the Pliocene and Pleistocene, have however facilitated the exchange of faunas and thus gene flow [46,74,97]. Accordingly, Trinidad shares many species with the Paria Range [37,47]. Recent work on the evolutionary history of the microteiids of the genus *Oreosaurus* has shown that, despite the distance between the highlands of the SNSM, CCR and TRI, their fauna shares an ancient connection [92]. Similarly, studies have suggested connections between the western and eastern areas of the CC, and TRI [98–102]. Interestingly, patterns of endemism in these highland areas have been observed in the Green Toucanets (genus *Aulacorhynchus*) with different subspecies in northeast Venezuela, western Venezuela and northern Venezuela–CC [102]. Such findings have also been observed in the Robinson’s Mouse Opossum, *Marmosa robinsoni*, with apparent western and eastern clades and shared haplotypes between Trinidad and Eastern Venezuela [44], with a northern coastal regional chorotype as inferred by marsupial species presence-absence [103]. Similarly, phylogenetic analyses on the Buthidae scorpion genus *Tityus* show relationships associated to geographically distinct areas, which suggest vicariance as a means of speciation in the region [100]. Again, the presence of a poor disperser as *Tytuis trinitatis* in TRI and in the PR suggests likely allopatric divergence when Trinidad detached from the mainland.

**Fig 11:**
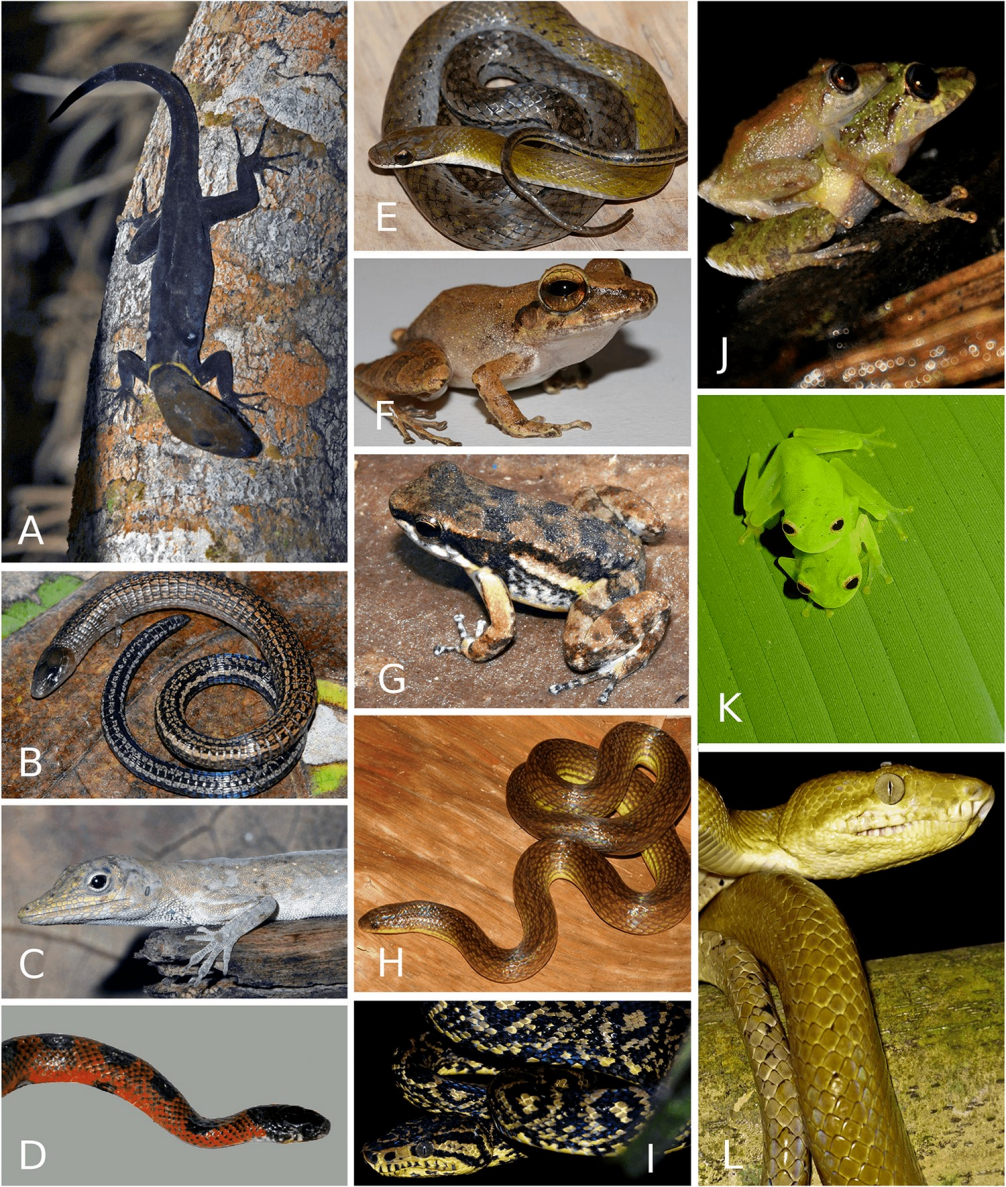
Amphibians and reptiles found on Tobago. A. The Ocellated Day Gecko, *Gonatodes ocellatus*. B. Square-scaled Bachia, *Bachia whitei* (Tobago endemic). C. Tobago Anole, *Anolis* cf *tigrinus*. D. Red Snake, *Erythrolamprus ocellatus* (Tobago endemic). E. Tobago Stream Snake, *Erythrolamprus pseudoreginae* (Tobago endemic). F. Charlotteville Litter Frog, *Pristimantis charlottevillensis* (Tobago endemic). G. Tobago Stream Frog, *Mannophryne olmonae* (Tobago endemic). H. Forest Ground Snake, *Atractus fuliginosa* on Tobago and in north central Venezuela. I and L Ruschenberger’s Treeboa, *Corallus ruschenbergerii* (different color morphs), J. Turpin’s Litter Frog, *Pristimantis turpinorum* (Tobago endemic). K. the Coastal Glassfrog, *Hyalinobatrachium orientale* (Tobago and northcentral Venezuela). Photo credits John C. Murphy (A-C, E-G, I-L), Richard M. Lehtinen (D), and Alvin L. Braswell (H).

The island of Tobago (Fig 9) has a total of eight endemic species (3 frogs, 2 lizards, and 3 snakes) [47] (Fig 11). Most of the TOB endemic species seem to have their closest living relatives in the CCR instead of the more proximal island of Trinidad. For example, the Central Coastal Range endemic stream frog *Mannophryne riveroi* is the sister to *M*. *olmonae* [85] and Tobago’s Charlotteville Litter Frog, *Pristimantis charlottevillensis* is related to *P*. *incertus* (as *P*. *terraebolivaris*) and members of the *P*. *conspicillatus* group [104]. As suggested by Jowers et al. [45], such relationships suggest a Pliocene “land bridge” connection between Tobago and central Venezuela in the past to explain the presence of the glass frog *Hyalinobatrachium orientale* on Tobago and northeastern Venezuela as it is likely with the ground snake *Atractus cf fuliginosus*. Another snake, the Tobago Stream Snake, *Erythrolamprus pseudoreginae* is known only from Tobago, but again its closest relatives are present in north-central Venezuela [31], not nearby Trinidad.

To date, the most extensive delimitation of biogeographic “provinces” in Northern Venezuela and its Caribbean coast are based on freshwater fish presence-absence classification records and ordination models [105]. Two faunas are currently recognized; Maracaibo and Caribbean, with a significant decline in species from east to west [34,105]. The reasons for such biogeographical zones are a consequence of geology, geomorphology, active tectonics, and past climatic conditions [96]. Throughout the Pliocene and Pleistocene, marine transgression isolated terrestrial taxa in mountainous areas, while marine regression opened new areas for colonization by terrestrial taxa dispersal. Molecular work on the killifish *Rivulus hartii* (= *Anablepsoides hartii*) has revealed recent dispersal events between the IMA, PR, and TRI-TOB throughout interglacial periods and by decreased marine salinity at that time [66]. Our results are consistent with the freshwater fish inventories which show that fewer species counts in the eastern Caribbean regions are less species richness than those the west [105].

### 4.2 Anthropogenic impacts

From a legal perspective, most of Venezuela’s bio-regions are well represented within the different conservation categories assigned by the government’s laws. The problem with the aforementioned legal framework is that the law itself does not establish clear procedures for administration and zoning of protected areas. In fact, there are no conservation areas as such. Despite the laws, the focus of most conservation organizations working in Venezuela over the past thirty years has been to conduct surveys and to do assessments that could inform zoning and regulation procedures within conservation areas, as well as maintaining up-to-date species fact-sheets at both national and international levels.

Almost two decades after the first surveys by some of this paper’s co-authors, the aforementioned efforts have revealed a much better understanding of Paria’s ecosystems, even though most efforts to influence environmental and conservation policy have failed. In retrospect, both national and international species fact-sheets are maintained and are up-to-date. This article is yet another effort to understand the region better and highlight its bio-diverse richness. Nevertheless, several high biodiversity areas are not parts of the national park system, such as Campeare, La Cerbatana, Cachipal, and the whole southern versant of the most significant peak, Cerro Humo. Despite the well-known connection between species of the lower mangrove forests in the Orinoco Delta and Paria Peninsula’s summits, there are no extant protected corridors between these two areas.

Notwithstanding the fact that all Venezuelan national parks are officially protected by law, the enforcement of such legislation is difficult. Illegal logging, hunting, and fires are important problems in the region, as is the introduction of invasive species, such as feral dogs, which roam freely in these parks. In addition, nearly every peak in the coastal range has communication infrastructure on it, and that infrastructure has replaced the pristine elfin forests and cloud forests there. Aggressive deforestation has occurred across the area since the early 20^th^ century and is well-documented [106]. The oil industry, active since the early 1960s, has severely impacted the habitat in the PR, as has the mining industry, which dates to the early 1940s. Lack of human oversight is also quite apparent in all national parks. For example, there are only two park rangers in the 37,500 ha of the Península de Paria National Park, and they are mainly without any equipment. In Paria, high cocoa prices have spurred extensive deforestation. Many of the cocoa plantations then since been abandoned due to declines in the economy. Fires and logging have the most detrimental effects and are the most imminent threats to the Paria Region. Each year, thousands of hectares of forest are lost to agricultural activity, which often begins by burning the forests.

Low altitude tropical cloud forests such as those found in PR and TRI are among the most vulnerable terrestrial ecosystems to climate change [107,108]. Some cloud forests have exceptional low altitudinal ranges (<950 in TRI and <1250 m in PR). In such systems, species adaptation to climatic conditions is crucial for survival as cloud cover can rapidly diminish with minor atmospheric changes. In the study area, cloud forest peaks are generally restricted to cloud/mist area (circa 300–600 m asl), and mountain summits are overall of relatively low elevations. Organisms inhabiting such environments cannot migrate up an altitudinal gradient to find more suitable conditions as they begin at their uppermost limits [107,109]. These relatively low altitude cloud forest are incredibly vulnerable to small changes in climate conditions; any resulting changes in forest habitat will directly impact the fauna. The higher-than-expected biodiversity in the high-risk PR montane forests suggest the need for detailed species inventories, population assessments, and the establishment of new, well-run, national parks to protect its biodiversity.

### 4.3 Final comments

This work results from several years of fieldwork in some of the most remote and challenging areas of Venezuela. Sucre State includes the PR and is one of the poorest economic and most degraded regions in Venezuela. The demise of the Venezuelan “petro-state” during the first decade of the 21^st^ century and the subsequent collapse of Venezuela’s economy has exacerbated historical and structural problems in this region. Pirates and drug traffickers have exploited its proximity to Trinidad and the Lesser Antilles. As a result, criminal activity has rendered fieldwork extremely dangerous [110]. This situation extends to the entire CCR and IMA, where high crime rates pose risks to researchers. Furthermore, low salaries, hyperinflation, a near-complete lack of scientific resources due to insufficient funding from both the private and public sectors, and the deterioration of the institutional infrastructure impose great difficulties to scientific research in Venezuela [111]. This study was achieved thanks to the kind collaboration of the local communities and through local agencies. We hope that studies such as this one will help other’s understanding of this unique biodiversity hotspot as well as the challenges of working there.

## Supporting information

S1 Table (DOCX)

S2 Table (DOCX)

S3 Table (DOCX)

S4 Table (DOCX)

S5 Table (DOCX)

S6 Table (DOCX)

S1 Text (DOCX)

S2 Text (DOCX)

S3 Text (DOCX)
