# Supplementary material for: Biogeographical patterns of amphibians and reptiles in the northernmost coastal montane complex of South America
Source: PLoS One. 2021 Mar 4;16(3):e0246829. doi: 10.1371/journal.pone.0246829 (PMC7932178; doi:10.1371/journal.pone.0246829)
Supplement: S1 Table — (DOCX) [file pone.0246829.s001.docx]

**S1 Table**

Survey localities throughout the Paria Region or Paria Range, dates, species collected and numbers of hours at each locality. Voucher specimens are deposited in the Museo de Biología, La Universidad del Zulia, Maracaibo (MBLUZ); Museo de la Estación Biológica de Rancho Grande, Maracay (EBRG), see Supporting Information 2 for details. The total survey effort was 32.7 h per person, and the survey effort per localities varied between 5.1 and 68.9 h per person. *Observed.

| **Localities** | **Date** | **Species collected** | **Survey effort** |
| --- | --- | --- | --- |
| Cerro La Cerbatana  10º37' N- 63º10' W | 10-12 Sept. 2013 (three days) | *Gonatodes ceciliae Gonatodes* sp*, Plica caribeana*, Tantilla melanocephala*, Ninia atrata*, Rhinella marina*, Leptodactylus* sp | Two observers; 43.4 person-hours. |
| Cerro La Cerbatana  10º37'N- 63º10' W | 11 Nov. 2016  (one day) | *Gonatodes* sp*, Copeoglossum aurae, Plica caribeana*, Oreosaurus rhodogaster, Thecadactyus rapicauda, Ameiva atrigullaris*, Cnemidophorus lemniscatus*, Mannophryne venezuelensis, Rhinella marina*, Flectonotus fitzgeraldi, Pristimantis nubisilva* | Two observers; 17.5 person-hours. |
| Campeare  10º32'45,4" N- 63º19'45, 6" W | 13 Nov. 2016  (one day) | *Gonatodes* sp*, Ameiva atrigullaris*, Cnemidophorus lemniscatus*, Mannophryne venezuelensis, Boana xerophylla*, Plica caribeana*, Hemidactylus mabouia, Pristimantis nubisilva.* | Two observers; 16.3 person-hours. |
| Campeare  10º32'45,4" N- 63º19'45, 6" | 20 Oct. 2017  (one day) | *Tantilla melanocephala, Ninia atrata, Mannophryne venezuelensis, Phyllomedusa trinitatis* | Two observers; 19.1 person-hours. |
| Cachipal   \| 10°38'02,5" N, 62°45'01,8" \| \| --- \| | 22-23 Oct. 2017  (two days) | *Gonatodes ceciliae, Mastigodryas boddaertii*, Bothrops venezuelensis, Mannophryne venezuelensis, Leptodactylus fuscus, Scinax ruber, Flectonotus fitzgeraldi, Pristimantis nubisilva* | Two observers; 30 person-hours. |
| Cerro Humo  10°41'32" N, 62°37' 52" W | 27-29 June, 2014  (three days) | *Gonatodes ceciliae, Gonatodes* sp, *Euspondylus monsfumus, Anadia pariaensis, Oreosaurus rhodogaster, Bachia trinitatis, Ninia atrata, Anolis tigrinus, Plica caribeana, Hyalinobatrachium orientale, Pristimantis nubisilva, P. pariagnomus, Flectonotus fitzgeraldi* | Two observers; 47.2 person-hours. |
| Cerro Humo  10°41'32" N, 62°37' 52" W | 29-31 August 2014 (three days) | *Anolis tigrinus, A. planiceps, Ninia atrata, Hyalinobatrachium orientale.* | Two observers; 45.8 person-hours. |
| Cerro Humo  10°41'32" N, 62°37' 52" W | 26-28 July 2015  (three days) | *Gonatodes ceciliae, Euspondylus monsfumus, Imantodes cenchoa, Phytotriades auratus, Pristimantis nubisilva* | Two observers; 51.1 person-hours. |
| Cerro Humo  10°41'32" N, 62°37' 52" W | 08-10 July 2016  (three days) | *Bothrops venezuelensis, Gonatodes ceciliae, Plica caribeana, Sibon nebulatus, Mannophryne venezuelensis, Phytotriades auratus, Pristimantis nubisilva, P. pariagnomus* | Three observers; 68.9 person-hours. |
| Macuro (trail between Macuro and Los Chorros)  10°41' 54 "N, 61°54' 45"W | 13 Aug. 2014  (one day) | *Pseudogonatodes* sp*, Tantilla melanocephala, Ninia trata, Bothrops venezuelensis, M. riveroi, M. venezuelensis, Vitreorana castroviejoi* | Two observers; 14.9 person-hours. |
| Cerro Las Melenas  10°41' 32.1 "N, 62°37' 24.9"W | 24 Oct. 2017  (one day) | *Bothrops venezuelensis*, Gonatodes ceciliae, Mannophryne venezuelensis, M. riveroi, Pristimantis nubisilva* | One observer; 5.1 person-hour. |
