# Supplementary material for: Biogeographical patterns of amphibians and reptiles in the northernmost coastal montane complex of South America
Source: PLoS One. 2021 Mar 4;16(3):e0246829. doi: 10.1371/journal.pone.0246829 (PMC7932178; doi:10.1371/journal.pone.0246829)
Supplement: S2 Table — (DOCX) [file pone.0246829.s002.docx]

**S3 Table**

Specimens identified through 16S rDNA sequencing and Genbank blasts. All from Paria Range (PR) Venezuela

| *Mannophryne venezuelensis* | Quebrada Seca, Cerro Campeare | EBRG 7341.  Genbank ac: MT975327 | 16S rDNA 99% identical *Mannophryne venezuelensis* |
| --- | --- | --- | --- |
| *Flectonotus fitzgeraldi* | Cerro La Cerbatana | EBRG 7337  Genbank ac: MT968886 | The 16S rDNA Genbank blast matched *Flectonotus fitzgeraldi* at 99% identity. The other individual from Cerro Campeare shared the same haplotype. |
| *Flectonotus fitzgeraldi* | Cerro Campeare | EBRG 7346  Genbank ac: MT968887 | 16S rDNA same haplotype as ERBG 7337 |
| *Pristimantis nubisilva* | Cerro Campeare | MBLUZ 426  Genbank ac: MT975334 | *p*-uncorrected mean distances between all *nubisilva* were 0.5% Match 95% *Pristimantis rozei.* |
| *Pristimantis nubisilva* | Cerro La Cerbatana | EBRG 7338  Genbank ac: MT975333 | *p*-uncorrected mean distances between all *P. nubisilva* were 0.4% Match 95% *Pristimantis rozei.* |
| *Phyllomedusa trinitatis* | Cerro Campeare | MBLUZ 431  Genbank ac: MT975329 | Match 100% *Phyllomedusa trinitatis* |
| *Copeoglossum* *aurae* | Cerro La Cerbatana | MBLUZ 1412  Genbank ac: MT975324 | The 16S rDNA Genbank blast matched 100% *Copeoglosum aurae* |
| *Ninia atrata* | Cerro Campeare | MBLUZ 1426  Genbank ac: MT975328 | DNA analysis of this specimen showed that this individual belongs to the species *Ninia atrata.* |
| *Pristimantis nubisilva* | Cachipal, Península de Paria, 800 m | MBLUZ 446  Genbank ac: MT975336 | *p*-uncorrected mean distances between all *P. nubisilva* were 0.4% Match 95% *Pristimantis rozei.* |
| *Pristimantis nubisilva* | South of Cerro Humo, Península de Paria, 800 m | MBLUZ 404  Genbank ac: MT975335 | *p*-uncorrected mean distances between all *P. nubisilva* were 0.4% Match 95% *Pristimantis rozei.* |
| *Pristimantis pariagnomus* | Southern versant of Cerro Humo, Península de Paria, 1000 m | MBLUZ 427–28.  Genbank ac: MT975331- MT975330 | *p*-uncorrected mean distances between all *P. pariagnomus* were 0.5% Match 95% *Pristimantis rozei.* Match 94% *Pristimantis rozei.* |
| *Leptodactylys fuscus* | Cachipal, Península de Paria, 800 m | MBLUZ 449  Genbank ac: MT975325 | 99% match to *Leptodactylus fuscus* |
| *Flectonotus fitzgeraldi* | South of Cerro Humo, Península de Paria, 800 m | MBLUZ 395, MBLUZ 448  Genbank ac: MT968884- MT968885 | Identical to the specimens from Cerro La Cerbatana and Cerro Camperare and to Trinidad specimens |
| *Mannophryne riveroi* | Quebrada Las Melenas, Península de Paria, 773 m | MBLUZ 450  Genbank ac: MT975326 | 100% match to *Mannophryne riveroi* |
| *Bachia trinitatis* | South of Cerro Humo, Península de Paria, 800 m | MBLUZ 1298  Genbank ac: MT975323 | 98% match to *B. h. trinitatis* from Trinidad. |
| *Pristimantis pariagnomus* | Quebada Las Melenas, Peninsula de Paria | MBLUZ461  Genbank ac: MT975332 | *p*-uncorrected mean distances between all *P. pariagnomus* were 0.5% Match 95% *Pristimantis rozei.* Match 94% *Pristimantis rozei.* |

Note: Because of no sequences of *Pristimantis nubisilva* and *P. pariagnomus* in Genbank, the closest matches were of *P. “rozei” (P. turpinorum)*. *P-*uncorrected mean distances between *Pristimantis nubisilva* and *P. pariagnomus* are 1.7%.

*P*-uncorrected distances between *Pristimantis nubisilva* and *P. pariagnomus*

|  | MBLUZ428 | MBLUZ427 | MBLUZ461 | EBRG7338 | MBLZ426 | MBLUZ404 | MBLUZ446 |
| --- | --- | --- | --- | --- | --- | --- | --- |
| *Pristimantis pariagnomus* MBLUZ428 | - |  |  |  |  |  |  |
| *Pristimantis pariagnomus* MBLUZ427 | 0,008 | - |  |  |  |  |  |
| *Pristimantis pariagnomus* MBLUZ461 | 0,006 | 0,002 | - |  |  |  |  |
| *Pristimantis nubisilva* EBRG7338 | 0,016 | 0,016 | 0,014 | - |  |  |  |
| *Pristimantis nubisilva* MBLUZ426 | 0,018 | 0,018 | 0,016 | 0,002 | - |  |  |
| *Pristimantis nubisilva* MBLUZ404 | 0,016 | 0,016 | 0,014 | 0,004 | 0,002 | - |  |
| *Pristimantis nubisilva* MBLUZ446 | 0,021 | 0,021 | 0,02 | 0,006 | 0,004 | 0,006 | - |
