# Supplementary material for: Biogeographical patterns of amphibians and reptiles in the northernmost coastal montane complex of South America
Source: PLoS One. 2021 Mar 4;16(3):e0246829. doi: 10.1371/journal.pone.0246829 (PMC7932178; doi:10.1371/journal.pone.0246829)
Supplement: S3 Table — (DOCX) [file pone.0246829.s003.docx]

**S4 Table**

List of all amphibians and reptiles in the studied region. SNSM (Sierra Nevada de Santa Marta, Colombia), SSL (Sierra de San Luis), CCR (Central Coastal Range), IMA (Isla de Margarita), TUR (Turimiquire Massif), PR (Paria Range), TRI (island of Trinidad), TOB (island of Tobago). EN (Endemic), INT (introduced).

|  |  |  |  |  |  |  |  |  |  |  |
| --- | --- | --- | --- | --- | --- | --- | --- | --- | --- | --- |
|  | SNSM | SSL | CCR | IMA | TUR | PR | TRI | TOB | EN | INT |
|  |  |  |  |  |  |  |  |  |  |  |
|  |  |  |  |  |  |  |  |  |  |  |
| **CLASS AMPHIBIA**  **ORDER ANURA** |  |  |  |  |  |  |  |  |  |  |
| **FAMILY AROMOBATIDAE** |  |  |  |  |  |  |  |  |  |  |
| *Allobates bromelicola* (Test 1956) |  |  | X |  |  |  |  |  | X |  |
| *Allobates caribe* (Barrio-Amorós, Rivas & Kaiser 2006) |  |  |  |  |  | X |  |  | X |  |
| *Allobates mandelorum* (Schmidt 1932) |  |  |  |  | X |  |  |  | X |  |
| *Allobates pittieri* (La Marca et al. 2004) |  | X | X |  |  |  |  |  |  |  |
| *Mannophryne herminae* (Boettger 1893) |  |  | X |  |  |  |  |  | X |  |
| *Mannophryne leonardoi* (Manzanilla et al. 2007) |  |  |  |  | X |  |  |  | X |  |
| *Mannophryne molinai* (Rojas-Runjaic et al. 2018) |  |  | X |  |  |  |  |  | X |  |
| *Mannophryne neblina* (Test 1956) |  |  | X |  |  |  |  |  | X |  |
| *Mannophrynre oblitterata* (Rivero 1984) |  |  | X |  |  |  |  |  | X |  |
| *Mannophryne olmonae* (Hardy 1983) |  |  |  |  |  |  |  | X | X |  |
| *Mannophryne riveroi* (Donoso-Barros 1964) |  |  |  |  |  | X |  |  | X |  |
| *Mannophryne trinitatis* (Garman 1887) |  |  |  |  |  |  | X |  | X |  |
| *Mannophryne venezuelensis* Manzanilla et al. 2007 |  |  |  |  |  | X |  |  | X |  |
| *Mannophryne vulcano* Barrio-Amorós et al. 2010 |  |  | X |  |  |  |  |  | X |  |
| *Mannophryne* sp. 1 |  | X |  |  |  |  |  |  | X |  |
| *Mannophryne* sp. 2 |  |  | X |  |  |  |  |  | X |  |
| *"Prostherapis" dunni* Rivero 1961 |  |  | X |  |  |  |  |  | X |  |
| **FAMILY DENDROBATIDAE** |  |  |  |  |  |  |  |  |  |  |
| *"Colostethus" ruthveni* Kaplan 1997 | X |  |  |  |  |  |  |  | X |  |
| *"Colostethus"* sp (*ruthveni-*like) | X |  |  |  |  |  |  |  | X |  |
| **FAMILY BUFONIDAE** |  |  |  |  |  |  |  |  |  |  |
| *Atelopus cruciger* (Lichtenstein & Martens 1856) |  |  | X |  |  |  |  |  | X |  |
| *Atelopus vogli* Müller 1934 |  |  | X |  |  |  |  |  | X |  |
| *Atelopus arsyecue* Rueda-Almonacid 1994 | X |  |  |  |  |  |  |  | X |  |
| *Atelopus carrikeri* Ruthven 1916 | X |  |  |  |  |  |  |  | X |  |
| *Atelopus laetissimus* Ruíz-Carranza et al. 1994 | X |  |  |  |  |  |  |  | X |  |
| *Atelopus nahumae* Ruíz-Carranza et al. 1994 | X |  |  |  |  |  |  |  | X |  |
| *Atelopus walkeri* Rivero 1963 | X |  |  |  |  |  |  |  | X |  |
| *Rhinella humboldti* (Gallardo 1965) | X |  |  |  |  |  |  |  |  |  |
| *Rhinella horribilis* (Wiegmann 1833) | X |  |  |  |  |  |  |  |  |  |
| *Rhinella marina* (Linnaeus 1758) |  | X | X | X | X | X | X | X |  |  |
| *Rhinella margartifera* group (Laurenti 1768) |  |  | X |  |  |  |  |  |  |  |
| *Rhinella sclerocephala* (Mijares-Urrutia & Arends 2001) |  | X |  |  |  |  |  |  | X |  |
| *Rhinella sternosignata*(Günther 1858) |  | X | X |  |  |  |  |  |  |  |
| **FAMILY CENTROLENIDAE** |  |  |  |  |  |  |  |  |  |  |
| *Celsiella vozmedianoi* (Ayarzagüena & Señaris 1997) |  |  |  |  |  | X |  |  | X |  |
| *Celsiella revocata* (Rivero 1985) |  |  | X |  |  |  |  |  | X |  |
| *Hyalinobatrachium orientale orientale* (Rivero 1968) |  |  |  |  | X | X |  |  |  |  |
| *Hyalinobatrachium orientale tobagoense* (Hardy 1984) |  |  |  |  |  |  |  | X | X |  |
| *Hyalinobatrachium orocostale* (Rivero 1968) |  |  | X |  |  |  |  |  | X |  |
| *Hyalinobatrachium fragile* (Rivero 1985) |  |  | X |  |  |  |  |  | X |  |
| *Hyalinobatrachium guairarepanensis* Señaris 2001 |  |  | X |  |  |  |  |  | X |  |
| *Hyalinobatrachium* sp. |  |  | X |  |  |  |  |  | X |  |
| *Ikakogi tayrona* (Ruiz-Carranza & Lynch 1991) | X |  |  |  |  |  |  |  | X |  |
| *Vitreorana castroviejoi* (Ayarzagüena & Señaris 1997) |  |  |  |  |  | X |  |  | X |  |
| *Vitreorana antisthenesi* (Goin 1963) |  |  | X |  |  |  |  |  | X |  |
| **FAMILY CERATOPHRYIDAE** |  |  |  |  |  |  |  |  |  |  |
| *Ceratophrys calcarata* Boulenger 1890 | X |  |  |  |  |  |  |  | X |  |
| **FAMILY LEPTODACTYLIDAE** |  |  |  |  |  |  |  |  |  |  |
| *Engystomops pustulosus* (Cope 1864) | X |  | X |  | X | X | X | X |  |  |
| *Leptodactylus fragilis* (Brocchi 1877) | X |  | X |  | X |  |  |  |  |  |
| *Leptodactylus fuscus* (Schneider 1799) | X |  | X | X | X | X | X | X |  |  |
| *Leptodactylus poecilochilus* (Cope 1862) | X |  | X |  |  |  |  |  |  |  |
| *Leptodactylus savagei* Heyer 2005 | X |  |  |  |  |  |  |  | X |  |
| *Leptodactylus turimiquensis* Heyer 2005 |  |  |  |  | X | X |  |  |  |  |
| *Leptodactylus* sp. |  |  |  |  |  | X |  |  | X |  |
| *Leptodactylus validus* Garman 1888 |  |  | X |  |  |  | X | X |  |  |
| *Physalaemus fischeri* (Boulenger 1890) |  |  | X |  |  |  |  |  |  |  |
| *Pleurodema brachyops* (Cope 1889) | X |  | X | X | X |  |  |  |  |  |
| *Pseudopaludicola pusilla* (Ruthven 1916) | X |  |  |  |  |  |  |  |  |  |
| **FAMILY HEMIPHRACTIDAE** |  |  |  |  |  |  |  |  |  |  |
| *Cryptobatrachus boulengeri* Ruthven 1916 | X |  |  |  |  |  |  |  | X |  |
| *Cryptobatrachus ruthveni* Lynch 2008 | X |  |  |  |  |  |  |  | X |  |
| *Flectonotus fitzgeraldi* (Parker 1934) |  |  |  |  |  | X | X | X |  |  |
| *Flectonotus pygmaeus* (Boettger 1893) |  | X | X |  |  |  |  |  |  |  |
| *Gastrotheca ovifera* (Lichtenstein & Weinland 1854) |  |  | X |  |  |  |  |  | X |  |
| *Gastrotheca walkeri* Duellman 1980 |  |  | X |  |  |  |  |  | X |  |
| *Gastrotheca williamsoni* Gaige 1922 |  |  | X |  |  |  |  |  | X |  |
| *Gastrotheca* sp. |  |  |  |  |  | X |  |  | X |  |
| **FAMILY STRABOMANTIDAE** |  |  |  |  |  |  |  |  |  |  |
| *Geobatrachus walkeri* Ruthven 1915 | X |  |  |  |  |  |  |  | X |  |
| *Pristimantis anotis* (Walker & Test 1955) |  |  | X |  |  |  |  |  | X |  |
| *Pristimantis bicumulus* (Peters 1864) |  |  | X |  |  |  |  |  | X |  |
| *Pristimantis charlottevillensis* (Kaiser et al. 1995) |  |  |  |  |  |  |  | X | X |  |
| *Pristimantis geminus* Kaiser et al. 2015 |  |  |  |  |  | X |  |  | X |  |
| *Pristimantis hoogmoedi* Kaiser et al. 2015 |  |  |  |  |  | X |  |  | X |  |
| *Pristimantis longicorpus* Kaiser et al. 2015 |  |  |  |  |  | X |  |  | X |  |
| *Pristimantis nubisilva* Kaiser et al. 2015 |  |  |  |  |  | X |  |  | X |  |
| *Pristimantis pariagnomus* Kaiser et al. 2015 |  |  |  |  |  | X |  |  | X |  |
| *Pristimantis reticulatus* (Walker & Test 1955) |  |  | X |  |  |  |  |  | X |  |
| *Pristimantis riveroi* (Lynch & La Marca 1993) |  |  | X |  |  |  |  |  | X |  |
| *Pristimantis rozei* (Rivero 1961) |  |  | X |  |  |  |  |  | X |  |
| *Pristimantis stenodiscus* (Walker & Test 1955) |  |  | X |  |  |  |  |  | X |  |
| *Pristimantis incertus* (Lutz 1924) |  |  | X |  |  |  |  |  | X |  |
| *Pristimantis turpinorum* (Hardy 2001) |  |  |  |  |  |  |  | X | X |  |
| *Pristimantis turimiquirensis* (Rivero 1961) |  |  |  |  | X |  |  |  | X |  |
| *Pristimantis urichi* (Boettger 1894) |  |  |  |  |  |  | X | X |  |  |
| *Pristimantis carmelitae* (Ruthven 1922) | X |  |  |  |  |  |  |  | X |  |
| *Pristimantis cristinae* (Lynch & Ruiz-Carranza 1985) | X |  |  |  |  |  |  |  | X |  |
| *Pristimantis delicatus* (Ruthven 1917) | X |  |  |  |  |  |  |  | X |  |
| *Pristimantis insignitus* (Ruthven 1917) | X |  |  |  |  |  |  |  | X |  |
| *Pristimantis megalops* (Ruthven 1917) | X |  |  |  |  |  |  |  | X |  |
| *Pristimantis ruthveni* (Lynch & Ruiz-Carranza 1985) | X |  |  |  |  |  |  |  | X |  |
| *Pristimantis sanctaemartae* (Ruthven 1917) | X |  |  |  |  |  |  |  | X |  |
| *Pristimantis tayrona* (Lynch & Ruiz-Carranza 1985) | X |  |  |  |  |  |  |  | X |  |
| *Pristimantis w-nigrum* (Boettger 1892) | X |  |  |  |  |  |  |  | X |  |
| *Pristimantis* sp. |  |  |  |  | X |  |  |  | X |  |
| *Strabomantis biporcatus* Peters 1863 |  |  | X |  |  | X |  |  |  |  |
| **FAMILY HYLIDAE** |  |  |  |  |  |  |  |  |  |  |
| *Boana boans* (Linnaeus 1758) |  |  |  |  |  | X | X |  |  |  |
| *Boana pugnax* (Schmidt 1857) | X |  |  |  |  |  |  |  |  |  |
| *Boana xerophylla* (Duméril and Bibron 1841) | X | X | X |  | X | X | X | X |  |  |
| *Dendropsophus battersbyi* (Rivero 1961) |  |  | X |  |  |  |  |  | X |  |
| *Dendropsophus luteocellatus* (Roux 1927) |  | X | X |  |  |  |  |  |  |  |
| *Dendropsophus microcephalus* (Cope 1886) | X | X | X |  | X | X | X | X |  |  |
| *Dendropsophus* aff. *minutus* (Peters 1872) |  | X | X |  |  |  |  |  |  |  |
| *Dendropsophus goughi* (Boulenger 1911) |  |  |  |  |  |  | X | X |  |  |
| *Dendropsophus yaracuyanus* (Mijares-Urrutia & Rivero 2000) |  |  | X |  |  |  |  |  | X |  |
| *Phytotriades auratus* (Boulenger 1917) |  |  |  |  |  | X | X |  |  |  |
| *Scinax ruber* (Laurenti 1768) | X |  |  |  | X | X | X | X |  |  |
| *Scinax rostratus* (Peters 1863) | X | X | X |  |  |  |  |  |  |  |
| *Scinax x-signatus* (Spix 1824) |  | X | X | X |  |  |  |  |  |  |
| *Trachycephalus typhonius* (Linnaeus 1758) | X |  | X |  | X | X | X | X |  |  |
| **FAMILY PHYLLOMEDUSIDAE** |  |  |  |  |  |  |  |  |  |  |
| *Agalychnis medinae* (Funkhouser 1962) |  |  | X |  |  |  |  |  | X |  |
| *Phyllomedusa trinitatis* Mertens 1926 |  | X | X |  | X | X | X |  |  |  |
| **FAMILY ELEUTHERODACTYLIDAE** |  |  |  |  |  |  |  |  |  |  |
| *Eleutherodactylus johnstonei* Barbour 1914 |  |  | X |  | X |  |  |  |  | X |
| **FAMILY RANIDAE** |  |  |  |  |  |  |  |  |  |  |
| *Lithobates vaillanti* (Brocchi 1877) | X |  |  |  |  |  |  |  | X |  |
| **ORDER CAUDATA**  **FAMILY PLETHODONTIDAE** |  |  |  |  |  |  |  |  |  |  |
| *Bolitoglossa borburata* Trapido 1942 |  |  | X |  |  |  |  |  | X |  |
| *Bolitoglossa savagei* (Brame & Wake 1963) | X |  |  |  |  |  |  |  | X |  |
| *Bolitoglossa* sp. |  | X |  |  |  |  |  |  | X |  |
| **ORDER GYMNOPHIONA**  **FAMILY CAECILIDAE** |  |  |  |  |  |  |  |  |  |  |
| *Caecilia flavopunctata* Roze 1963 |  |  | X |  |  |  |  |  | X |  |
|  |  |  |  |  |  |  |  |  |  |  |
| Total | 38 | 14 | 53 | 4 | 17 | 26 | 15 | 15 | 79 | 1 |
|  |  |  |  |  |  |  |  |  |  |  |
|  |  |  |  |  |  |  |  |  |  |  |
|  | SNSM | SSL | CCR | IMA | TUR | PR | TRI | TOB | EN | INT |
|  |  |  |  |  |  |  |  |  |  |  |
|  |  |  |  |  |  |  |  |  |  |  |
| **CLASS REPTILIA** |  |  |  |  |  |  |  |  |  |  |
| **ORDER CROCODYLIA** |  |  |  |  |  |  |  |  |  |  |
| **FAMILY ALLIGATORIDAE** |  |  |  |  |  |  |  |  |  |  |
| *Caiman crocodilus* Linnaeus 1758 |  |  | X |  |  |  |  |  |  | X |
| **ORDER TESTUDINES** |  |  |  |  |  |  |  |  |  |  |
| **FAMILY KINOSTERNIDAE** |  |  |  |  |  |  |  |  |  |  |
| *Kinosternon scorpioides* (Linnaeus 1766) |  | X | X |  |  |  |  |  |  |  |
| **FAMILY PODOCNEMIDIDAE** |  |  |  |  |  |  |  |  |  |  |
| *Podocnemis expansa* (Schweigger 1812) |  |  | X |  |  |  |  |  |  | X |
| **ORDER SQUAMATA** |  |  |  |  |  |  |  |  |  |  |
| *Amphisbaena alba* (Linnaeus 1758) |  |  | X | X | X | X | X |  |  |  |
| *Amphisbaena fuliginosa* (Linnaeus 1758) | X |  | X |  | X | X | X |  |  |  |
| **FAMILY CORYTOPHANIDAE** |  |  |  |  |  |  |  |  |  |  |
| *Basiliscus basiliscus* (Linnaeus 1758) | X |  | X |  |  |  |  |  |  |  |
| **FAMILY IGUANIDAE** |  |  |  |  |  |  |  |  |  |  |
| *Iguana iguana* (Linnaeus 1758) | X |  | X | X | X | X | X | X |  |  |
| *Ctenosaura similis* (Gray 1831) |  |  |  |  | X |  |  |  |  | X |
| **FAMILY DACTYLOIDAE** |  |  |  |  |  |  |  |  |  |  |
| *Anolis aeneus* (Gray 1840) |  |  |  |  |  |  | X | X |  | X |
| *Anolis auratus* Daudin 1802 | X |  |  |  | X |  |  |  |  |  |
| *Anolis biporcatus* (Wiegmann 1834) | X |  |  |  |  |  |  |  |  |  |
| *Anolis extremus* Garman 1887 |  |  | X |  |  |  |  |  |  | X |
| *Anolis fuscoauratus* Duméril & Bibron 1837 |  | X | X |  |  |  |  |  |  |  |
| *Anolis gaigei* Ruthven 1916 | X |  |  |  |  |  |  |  |  |  |
| *Anolis menta* Ayala, Harris & Williams 1984 | X |  |  |  |  |  |  |  | X |  |
| *Anolis onca* (O'Shaughnessy 1875) |  |  |  | X |  |  |  |  |  |  |
| *Anolis paravertebralis* Bernal-Carlo & Roze 2005 | X |  |  |  |  |  |  |  | X |  |
| *Anolis planiceps* Troschel 1848 |  | X | X | X | X | X | X |  |  |  |
| *Anolis richardii* (Duméril & Bibron 1837) |  |  |  |  |  |  |  | X |  | X |
| *Anolis santamartae* Williams 1982 | X |  |  |  |  |  |  |  | X |  |
| *Anolis solitarius* Ruthven 1916 | X |  |  |  |  |  |  |  | X |  |
| *Anolis squamulatus* Peters 1863 |  |  | X |  |  |  |  |  | X |  |
| *Anolis tigrinus* Peters 1863 |  | X | X |  | X | X |  |  |  |  |
| *Anolis trinitatis* (Reinhardt & Lütken 1862) |  |  |  |  |  |  | X |  |  | X |
| *Anolis umbrivagus* Bernal-Carlo & Roze 2005 | X |  |  |  |  |  |  |  | X |  |
| *Anolis* sp. |  |  |  |  |  |  | X | X |  |  |
| **FAMILY POLYCHROTIDAE** |  |  |  |  |  |  |  |  |  |  |
| *Polychrus auduboni* (Hallowell 1845) |  |  | X | X | X | X | X | X |  |  |
| *Polychrus gutturosus* Berthold 1846 | X |  |  |  |  |  |  |  |  |  |
| *Polychrus "marmoratus"*(Linnaeus 1758) | X |  |  |  |  |  |  |  |  |  |
| **FAMILY TROPIDURIDAE** |  |  |  |  |  |  |  |  |  |  |
| *Plica caribeana* Murphy & Jowers 2013 |  |  | X |  | X | X | X |  |  |  |
| *Stenocercus erythrogaster* (Hallowell 1856) | X |  |  |  |  |  |  |  |  |  |
| *Tropidurus hispidus* (Spix 1825) |  |  | X | X | X | X |  |  |  |  |
| **FAMILY SPHAERODACTYLIDAE** |  |  |  |  |  |  |  |  |  |  |
| *Gonatodes albogularis* (Duméril & Bibron 1836) | X |  |  |  |  |  |  |  |  |  |
| *Gonatodes ceciliae* Donoso-Barros 1966 |  |  |  |  |  | X | X |  |  |  |
| *Gonatodes falconensis* Shreve 1947 |  | X | X |  |  |  |  |  |  |  |
| *Gonatodes ferrugineus* Cope 1964 |  |  |  |  |  |  | X |  | X |  |
| *Gonatodes humeralis* (Guichenot 1855) |  |  | X |  |  | X |  |  |  |  |
| *Gonatodes ocellatus* (Gray 1831) |  |  |  |  |  |  |  | X | X |  |
| *Gonatodes rozei* Rivero-Blanco & Schargel 2012 |  |  | X |  |  |  |  |  | X |  |
| *Gonatodes seigliei* Donoso-Barros 1966 |  |  |  |  | X |  |  |  | X |  |
| *Gonatodes taniae* Roze 1963 |  |  | X |  |  |  |  |  | X |  |
| *Gonatodes vittatus* (Lichtenstein 1856) | X | X | X | X | X | X | X | X |  |  |
| *Gonatodes machelae* Rivero-Blanco & Schargel 2020 |  |  |  | X |  |  |  |  | X |  |
| *Gonatodes* sp. |  |  |  |  | X | X |  |  |  |  |
| *Lepidoblepharis miyatai* Lamar 1985 | X |  |  |  |  |  |  |  |  |  |
| *Lepidoblepharis sanctaemartae* (Ruthven 1916) | X |  |  |  |  |  |  |  |  |  |
| *Pseudogonatodes furvus* Ruthven1915 | X |  |  |  |  |  |  |  | X |  |
| *Pseudogonatodes lunulatus* (Roux 1927) |  |  | X |  |  |  |  |  |  |  |
| *Pseudogonatodes manessi* Avila-Pires & Hoogmoed 2000 |  |  | X |  |  |  |  |  | X |  |
| *Pseudogonatodes* sp. |  |  |  |  |  | X |  |  | X |  |
| *Sphaerodactylus heliconiae* Harris1982 | X |  |  |  |  |  |  |  |  |  |
| *Sphaerodactylus molei* Boettger 1894 |  |  | X | X |  | X | X | X |  |  |
| **FAMILY GEKKONIDAE** |  |  |  |  |  |  |  |  |  |  |
| *Hemidactylus angulatus* Hallowell 1854 | X |  |  |  |  |  |  |  |  | X |
| *Hemidactylus frenatus* Schlegel 1836 | X |  | X | X |  |  |  |  |  | X |
| *Hemidactylus mabouia* (Moreau de Jonnés 1818) |  |  | X | X | X | X | X | X |  | X |
| *Hemidactylus palaichthus* Kluge 1969 |  |  |  |  |  | X | X | X |  |  |
| *Lepidodactylus lugubris* (Dumeril & Bibron 1836) |  |  | X |  |  |  |  |  |  | X |
| **FAMILY PHYLLODACTYLIDAE** |  |  |  |  |  |  |  |  |  |  |
| *Phyllodactylus ventralis* O'Shaughnessy 1875 | X | X | X | X |  | X |  |  |  |  |
| *Thecadactylus rapicauda* (Houttuyn 1782) | X | X | X | X | X | X | X | X |  |  |
| **FAMILY GYMNOPHTHALMIDAE** |  |  |  |  |  |  |  |  |  |  |
| *Anadia altaserrania* Harris & Ayala 1987 | X |  |  |  |  |  |  |  | X |  |
| *Anadia blakei* Schmidt 1932 |  |  |  |  | X | X |  |  |  |  |
| *Anadia marmorata* (Gray 1846) |  |  | X |  |  |  |  |  | X |  |
| *Anadia pariaensis* Rivas, La Marca & Oliveros 1999 |  |  |  |  |  | X |  |  | X |  |
| *Anadia pulchella* Ruthven 1926 | X |  |  |  |  |  |  |  | X |  |
| *Anadia steyeri* Nieden 1914 |  | X | X |  |  |  |  |  |  |  |
| *Bachia bicolor* (Cope 1896) | X |  |  |  |  |  |  |  |  |  |
| *Bachia heteropa* (Lichhtenstein 1856) |  |  | X | X |  |  |  |  |  |  |
| *Bachia talpa* Ruthven 1925 |  |  |  |  |  |  |  |  |  |  |
| *Bachia trinitatis* (Barbour 1914) |  |  |  | X |  | X | X | X |  |  |
| *Bachia whitei* Murphy et al. 2019 |  |  |  |  |  |  |  | X | X |  |
| *Euspondylus acutirostris* (Peters 1862) |  | X | X |  |  |  |  |  |  |  |
| *Euspondylus monsfumus* Mijares-Urrutia et al. 2001 |  |  |  |  |  | X |  |  | X |  |
| *Gymnophthalmus* sp. | X |  | X | X | X | X |  |  |  |  |
| *Gymnophthalmus undewoodi* (Grant 1958) |  |  |  |  |  |  | X | X |  |  |
| *Oreosaurus achlyens* (Uzzell 1958) |  |  | X |  |  |  |  |  | X |  |
| *Oreosaurus luctuosus* (Peters 1862) |  |  | X |  |  |  |  |  | X |  |
| *Oreosaurus rhodogaster* (Rivas, Schargel & Meik 2005) |  |  |  |  |  | X |  |  | X |  |
| *Oreosaurus serranus* Sánchez-Pacheco et al. 2017 | X |  |  |  |  |  |  |  | X |  |
| *Oreosaurus shrevei* (Parker 1935) |  |  |  |  |  |  | X |  | X |  |
| *Oreosaurus* sp. |  |  |  |  | X |  |  |  | X |  |
| *Tretioscincus bifasciatus* (Duméril 1851) | X |  | X | X | X | X |  |  |  |  |
| **FAMILY ALOPOGLOSSIDAE** |  |  |  |  |  |  |  |  |  |  |
| *Ptychoglossus kugleri* Roux 1927 |  |  | X |  |  |  |  |  | X |  |
| *Ptychoglossus romaleos* Harris 1994 | X |  |  |  |  |  |  |  | X |  |
| **FAMILY TEIIDAE** |  |  |  |  |  |  |  |  |  |  |
| *Ameiva atrigullaris* (Garman 1887) |  | X | X | X | X | X | X | X |  |  |
| *Ameiva bifrontata* Cope 1862 | X | X | X | X |  |  |  |  |  |  |
| *Ameiva praesignis* (Baird & Girard 1852) | X |  |  |  |  |  |  |  |  |  |
| *Cnemidophorus gaigae* Ruthven 1915 | X |  |  |  |  |  |  |  |  |  |
| *Cnemidophorus lemniscatus* (Linnaeus 1758) |  |  | X |  | X | X |  |  |  |  |
| *Cnemidophorus senectus* Ugueto et al 2009 |  |  |  | X |  |  |  |  | X |  |
| *Kentropyx striata* (Daudin 1802) |  |  | X |  |  |  |  |  |  |  |
| *Tupinambis cryptus* (Murphy et al. 2016) |  |  | X | X | X |  | X | X |  |  |
| *Tupinambis* aff. *teguixin* (Linnaeus 1758) | X |  |  |  |  |  |  |  |  |  |
| **FAMILY MABUYIDAE** |  |  |  |  |  |  |  |  |  |  |
| *Copeoglossum aurae* (Hedges & Conn 2012) |  |  | X |  |  | X | X | X |  |  |
| *Copeoglossum margaritae* (Hedges & Conn 2012) |  |  |  | X |  |  |  |  | X |  |
| *Copeoglossum* sp. |  | X | X |  |  |  |  |  |  |  |
| *Marisora falconensis* (Mijares-Urrutia & Arends 1997) |  |  | X |  |  |  |  |  |  |  |
| *Orosaura nebulosylvestris* (Miralles et al. 2009) |  |  | X |  |  |  |  |  | X |  |
| *Panopa croizati* (Horton 1973) |  |  |  |  | X |  |  |  | X |  |
| *"Mabuya"* sp | X |  |  |  |  |  |  |  |  |  |
| **FAMILY BOIDAE** |  |  |  |  |  |  |  |  |  |  |
| *Boa constrictor* Linnaeus 1758 | X | X | X | X | X | X | X | X |  |  |
| *Corallus ruschenbergerii* (Cope 1876) | X |  | X | X | X | X | X | X |  |  |
| *Epicrates maurus* (Gray 1849) | X |  | X | X | X |  | X | X |  |  |
| **FAMILY COLUBRIDAE** |  |  |  |  |  |  |  |  |  |  |
| *Chironius carinatus* (Linnaeus 1758) | X |  |  |  |  | X | X |  |  |  |
| *Chironius spixi* (Hallowell 1845) |  |  | X |  | X |  |  |  |  |  |
| *Chironius monticola* Roze 1952 |  | X | X |  |  |  |  |  |  |  |
| *Chironius septentrionalis* (Dixon, Wiest & Cei 1993) |  |  | X |  | X |  | X |  |  |  |
| *Coluber mentovarius* (Duméril et al., 1854) | X |  | X | X | X |  |  |  |  |  |
| *Dendrophidion percarinatum* (Cope 1893) | X |  |  |  |  |  |  |  |  |  |
| *Dendrophidion nuchale* (Peters 1864) |  |  | X |  |  |  |  |  | X |  |
| *Drymarchon caudomaculatus* Wuster et al. 2001 |  | X |  |  |  |  |  |  |  |  |
| *Drymarchon corais* (Boie 1827) |  |  | X |  | X | X | X | X |  |  |
| *Drymarchon margaritae* (Roze 1959) |  |  |  | X |  |  |  |  | X |  |
| *Drymarchon melanurus* (Duméril et al. 1854) | X |  | X |  |  |  |  |  |  |  |
| *Drymobius rhombifer* (Günther 1860) | X |  |  |  |  |  |  |  |  |  |
| *Lampropeltis micropholis* (Cope 1861) | X |  |  |  |  |  |  |  |  |  |
| *Leptophis coeruleodorsus* (Oliver 1942) |  |  | X | X | X |  | X | X |  |  |
| *Leptophis haileyi* Murphy et al. 2013 |  |  |  |  |  |  |  | X | X |  |
| *Leptophis occidentalis* (Gunther 1859) | X | X | X |  |  |  |  |  |  |  |
| *Leptophis stimsoni* Harding 1995 |  |  |  |  |  |  | X |  | X |  |
| *Mastigodryas amarali* (Stuart 1938) |  |  |  | X | X |  |  |  |  |  |
| *Mastigodryas boddaerti* (Sentzen 1796) | X | X | X |  | X | X | X |  |  |  |
| *Mastigodryas dunni* (Stuart 1933) |  |  |  |  | X |  |  | X |  |  |
| *Mastigodryas pleei* (Duméril et al., 1854) | X |  | X | X |  |  |  |  |  |  |
| *Oxybelis aeneus* (Wagler 1824) | X |  | X | X | X | X | X | X |  |  |
| *Oxybelis fulgidus* (Daudin 1803) | X |  |  |  |  | X |  |  |  |  |
| *Phrynonax polylepis* (Peters 1867) |  |  |  |  |  |  | X |  | X |  |
| *Phrynonax shropshirei* (Barbour & Amaral 1924) | X |  | X |  |  |  |  |  |  |  |
| *Spilotes sulphureus* (Wagler & Spix 1824) |  |  |  |  |  |  | X |  |  |  |
| *Spilotes pullatus* (Linnaeus 1758) | X | X | X | X | X | X | X | X |  |  |
| *Stenorrhina degenhardtii* (Berthold 1846) | X | X | X |  |  |  |  |  |  |  |
| *Tantilla melanocephala* (Linnaeus 1758) | X |  | X |  | X | X | X | X |  |  |
| *Tantilla semicincta* (Duméril, Bibron & Duméril 1854) | X |  |  |  |  |  |  |  |  |  |
| **FAMILY DIPSADIDAE** |  |  |  |  |  |  |  |  |  |  |
| *Atractus fuliginosus* (Hallowell 1845) |  |  | X |  |  |  |  | X |  |  |
| *Atractus lancinii* Roze 1961 |  |  | X |  |  |  |  |  | X |  |
| *Atractus matthewi* Markezich & Barrio-Amorós 2004 |  |  |  |  | X |  |  |  | X |  |
| *Atractus sanctaemartae* Dunn 1946 | X |  |  |  |  |  |  |  | X |  |
| *Atractus trilineatus* Wagler 1828 |  |  |  |  | X |  | X | X |  |  |
| *Atractus vittatus* Boulenger 1894 |  |  | X |  |  |  |  |  | X |  |
| *Clelia clelia* (Daudin 1803) | X |  | X |  | X |  |  |  |  |  |
| *Dipsas praeornata* Werner 1909 |  |  | X |  |  |  |  |  | X |  |
| *Dipsas variegata* (Duméril, Bibron & Duméril 1854) |  |  | X |  | X |  | X |  |  |  |
| *Enulius flavitorques*(Cope 1868) | X |  |  |  |  |  |  |  |  |  |
| *Erythrolamprus aesculapii* (Linnaeus 1766) |  |  |  |  | X |  | X |  |  |  |
| *Erythrolamprus bizona* Jan 1863 | X | X | X |  |  |  | X |  |  |  |
| *Erythrolamprus epinephellus* (Cope 1862) | X |  |  |  |  |  |  |  |  |  |
| *Erythrolamprus melanotus* (Shaw 1802) | X |  | X | X | X | X | X | X |  |  |
| *Erythrolamprus mertensi* (Roze 1964) |  | X | X |  |  |  |  |  |  |  |
| *Erythrolamprus ocellatus* (Peters 1868) |  |  |  |  |  |  |  | X | X |  |
| *Erythrolamprus pseudoreginae* Murphy et al. 2019 |  |  |  |  |  |  |  | X | X |  |
| *Erythrolamprus williamsi* (Roze 1958) |  |  | X |  |  |  |  |  | X |  |
| *Erythrolamprus zweifeli* (Roze 1959) |  | X | X |  | X | X | X |  |  |  |
| *Helicops danieli* Amaral 1938 | X |  |  |  |  |  |  |  |  |  |
| *Imantodes cenchoa* (Linnaeus 1758) | X | X | X |  |  | X | X | X |  |  |
| *Leptodeira ashmeadi* (Hallowell 1845) | X | X | X | X | X |  | X | X |  |  |
| *Leptodeira ornata* (Bocourt 1884) | X |  |  |  |  |  |  |  |  |  |
| *Lygophis lineatus*(Linnaeus 1758) | X |  |  |  |  |  |  |  |  |  |
| *Ninia atrata* (Hallowell 1845) | X | X | X |  | X | X | X | X |  |  |
| *Ninia franciscoi* Angarita-Sierra 2014 |  |  |  |  |  |  | X |  | X |  |
| *Oxyrhopus doliatus* (Duméril, Bibron & Duméril 1854) |  | X | X |  |  |  |  |  |  |  |
| *Oxyrhopus petolarius* (Linnaeus 1758) | X |  | X |  | X | X | X | X |  |  |
| *Phimophis guianensis* (Troschel & Schomburgk 1848) | X |  | X | X |  |  |  |  |  |  |
| *Philodryas olfersii* (Wied-Neuwied 1825) |  |  | X |  |  |  |  |  |  |  |
| *Pseudoboa neuwiedii* (Duméril et al. 1854) | X | X | X | X | X |  | X | X |  |  |
| *Sibon nebulatus* (Linnaeus 1758) | X | X | X | X | X | X | X | X |  |  |
| *Siphlophis cervinus* (Laurenti 1768) |  |  |  |  |  | X | X |  |  |  |
| *Siphlophis compressus* (Daudin 1803) |  |  |  |  |  | X | X |  |  |  |
| *Taeniophallus nebularis* Schargel, Rivas & Myers 2005 |  |  |  |  |  | X |  |  | X |  |
| *Thamnodynastes gambotensis* Pérez-Santos & Moreno 1989 | X |  |  |  |  |  |  |  | X |  |
| *Thamnodynastes ramonriveroi* Manzanilla & Sánchez 2005 |  |  |  |  | X |  |  |  |  |  |
| *Urotheca multilineata* (Peters 1859) |  |  | X |  |  |  |  |  | X |  |
| *Xenodon severus* (Linnaeus 1758) | X | X | X |  |  |  |  |  |  |  |
| **FAMILY ELAPIDAE** |  |  |  |  |  |  |  |  |  |  |
| *Micrurus circinalis* (Duméril and Bibron 1854) |  |  |  |  | X | X | X |  |  |  |
| *Micrurus dissoleucus* (Cope 1860) | X |  | X |  |  |  |  |  |  |  |
| *Micrurus dumerilii* (Jan 1858) | X |  | X |  |  |  |  |  |  |  |
| *Micrurus isozonus* (Cope 1860) |  |  | X | X | X | X |  |  |  |  |
| *Micrurus diutus* (Burger 1955) |  |  | X |  | X |  | X |  |  |  |
| *Micrurus mipartitus* (Duméril, Bibron & Duméril 1854) | X | X | X |  |  |  |  |  |  |  |
| **FAMILY VIPERIDAE** |  |  |  |  |  |  |  |  |  |  |
| *Bothrops asper* (Garman 1883) | X |  |  |  |  |  |  |  |  |  |
| *Bothrops atrox* (Linnaeus 1758) |  |  |  |  |  |  | X |  |  |  |
| *Bothrops colombiensis* (Garman 1883) |  | X | X |  |  |  |  |  |  |  |
| *Bothrops medusa* (Sternfeld 1920) |  |  | X |  |  |  |  |  | X |  |
| *Bothrops venezuelensis* Sandner-Montilla 1952 |  |  | X |  | X | X |  |  |  |  |
| *Crotalus durissus* Linnaeus 1758 | X |  | X | X | X |  |  |  |  |  |
| *Lachesis muta* (Linnaeus 1766) |  |  |  |  | X | X | X |  |  |  |
| *Porthidium lansbergii* (Schlegel 1841) | X |  | X | X | X |  |  |  |  |  |
| **FAMILY ANOMALEPIDIDAE** |  |  |  |  |  |  |  |  |  |  |
| *Helminthophis flavoterminatus* (Peters 1857) |  |  | X |  |  |  | X |  |  |  |
| *Liotyphlops albirostris* (Peters 1881) | X |  | X |  |  |  |  |  |  |  |
| **FAMILY LEPTOTYPHLOPIDAE** |  |  |  |  |  |  |  |  |  |  |
| *Epictia* cf. *fallax* (Peters 1858) |  |  |  |  |  |  | X |  | X |  |
| *Epictia tenella* (Klauber 1939) |  |  |  |  | X | X | X |  |  |  |
| *Epictia goudotii* (Duméril & Bibron 1844) |  | X | X | X |  |  |  |  |  |  |
| *Trilepida macrolepis* (Peters 1857) | X |  | X |  |  |  |  |  |  |  |
| **FAMILY TYPHLOPIDAE** |  |  |  |  |  |  |  |  |  |  |
| *Amerotyphlops reticulatus* (Linnaeus 1766) |  |  | X |  |  |  |  |  |  |  |
| *Amerotyphlops trinitatus* Richmnond 1965 |  |  |  |  |  |  | X | X |  |  |
|  |  |  |  |  |  |  |  |  |  |  |
| **TOTAL** | 81 | 32 | 102 | 41 | 56 | 50 | 59 | 39 | 52 | 11 |
|  |  |  |  |  |  |  |  |  |  |  |

Several species have been considered in this study as “Exclusive” but treated in the richness analysis as “Unique to a region” due the fact that they only occur in a single mountain chain within our study area. However, besides being found in this mountain chain, they also occur elsewhere in South America. These species are: *Rhinella horribilis, R. humboldti, Ceratophrys calcarata, Physalaemus fisheri, Pseudopaludicola pusilla, Boana pugnax, Anolis biporcatus, Stenocercus erythrogaster, Gonatodes albogularis, Lepidoblepharis miyatai, L. sanctaemartae, Sphaerodactylus heliconiae, Bachia bicolor, Bachia talpa, Polychrus gutturosus, P. “marmoratus”, Ameiva praesignis, Cnemidophrus gaigae, Tupinambis “teguixin”, Bothrops asper, Dendrophidon percarinatum, Drymobius rhombifer, Enulius flavitorques, Erythrolamprus epinephellus, Helicops danieli, Lampropeltis triangulum, Leptodeira ornata, Lygophis lineatus, Tantilla semicincta, Thamnodynastes gambotensis* (SNSM), *Drymarchon caudomaculatus* (SSL), *Ptychoglossus kugleri, Pseudogonatodes lunulatus, Kentropyx striata, Marisora falconensis, Oreosaura nebulosylvestris, Amerotyphlops reticulatus, Dendrophidion nuchale, Philodryas olfersii, Urotheca multilineata* (CCR), *Anolis onca* (IMA), *Thamnodynastes ramonriveroi* (TUR), *Phrynonax polylepis* (PR), *Bothrops atrox* and *Spilotes sulphureus* (TRI). Also, while the term “Endemic” in its strict sense is used here to refer to those species that occurs in a single mountain system but nowhere else, and are the key species we used for biotas’ identification.
