# Supplementary material for: Biogeographical patterns of amphibians and reptiles in the northernmost coastal montane complex of South America
Source: PLoS One. 2021 Mar 4;16(3):e0246829. doi: 10.1371/journal.pone.0246829 (PMC7932178; doi:10.1371/journal.pone.0246829)
Supplement: S4 Table — (DOCX) [file pone.0246829.s004.docx]

**S5 Table**

List of exclusive and restricted species of amphibians and reptiles to humid or evergreen mountain forests in the studied region. SNSM (Sierra Nevada de Santa Marta, Colombia), SSL (Sierra de San Luis), CCR (Central Coastal Range), IMA (Isla de Margarita), TUR (Turimiquire Massif), PR (Paria Range), TRI (island of Trinidad), TOB (island of Tobago).

|  | | | | **SNSM** | | **SSL** | | **CCR** | | **IMA** | | **TUR** | **PR** | **TRI** | **TOB** |
| --- | --- | --- | --- | --- | --- | --- | --- | --- | --- | --- | --- | --- | --- | --- | --- |
|  | | | |  | |  | |  | |  | |  |  |  |  |
|  | | | |  | |  | |  | |  | |  |  |  |  |
| **CLASS AMPHIBIA** | | | |  | |  | |  | |  | |  |  |  |  |
| **ORDER ANURA** |  |  |  |  |  |  |  |  |  |  |  |  |  |  |  |
| **FAMILY AROMOBATIDAE** | | | |  | |  | |  | |  | |  |  |  |  |
| *Allobates bromelicola* (Test 1956) | | | |  | |  | | X | |  | |  |  |  |  |
| *Allobates caribe* (Barrio-Amorós et al. 2006) | | | |  | |  | |  | |  | |  | X |  |  |
| *Allobates mandelorum* (Schmidt 1932) | | | |  | |  | |  | |  | | X |  |  |  |
| *Mannophryne herminae* (Boettger 1893) | | | |  | |  | | X | |  | |  |  |  |  |
| *Mannophryne leonardoi* (Manzanilla et al. 2007) | | | |  | |  | |  | |  | | X |  |  |  |
| *Mannophryne molinai* (Rojas-Runjaic et al. 2108) | | | |  | |  | | X | |  | |  |  |  |  |
| *Mannophryne neblina* (Test 1956) | | | |  | |  | | X | |  | |  |  |  |  |
| *Mannophryne vulcano* Barrio-Amorós et al. 2010 | | | |  | |  | | X | |  | |  |  |  |  |
| "*Prostherapis*" *dunni* Rivero 1961 | | | |  | |  | | X | |  | |  |  |  |  |
| **FAMILY DENDROBATIDAE** | | | |  | |  | |  | |  | |  |  |  |  |
| "*Colostethus" ruthveni* Kaplan 1997 | | | | X | |  | |  | |  | |  |  |  |  |
| "*Colostethus*" sp (ruthveni-like) | | | | X | |  | |  | |  | |  |  |  |  |
| **FAMILY BUFONIDAE** | | | |  | |  | |  | |  | |  |  |  |  |
| *Atelopus arsyecue* Rueda-Almonacid 1994 | | | | X | |  | |  | |  | |  |  |  |  |
| *Atelopus carrikeri* Ruthven 1916 | | | | X | |  | |  | |  | |  |  |  |  |
| *Atelopus laetissimus* Ruíz-Carranza et al. 1994 | | | | X | |  | |  | |  | |  |  |  |  |
| *Atelopus nahumae* Ruíz-Carranza et al. 1994 | | | | X | |  | |  | |  | |  |  |  |  |
| *Atelopus walkeri* Rivero 1963 | | | | X | |  | |  | |  | |  |  |  |  |
| *Rhinella margartifera* group (Laurenti 1768) | | | |  | |  | | X | |  | |  |  |  |  |
| *Rhinella sclerocephala* (Mijares-Urrutia & Arends 2001) | | | |  | | X | |  | |  | |  |  |  |  |
| **FAMILY CENTROLENIDAE** | | | |  | |  | |  | |  | |  |  |  |  |
| *Celsiella vozmedianoi* (Ayarzagüena & Señaris 1997) | | | |  | |  | |  | |  | |  | X |  |  |
| *Celsiella revocata* (Rivero 1985) | | | |  | |  | | X | |  | |  |  |  |  |
| *Hyalinobatrachium orocostale* (Rivero 1968) | | | |  | |  | | X | |  | |  |  |  |  |
| *Hyalinobatrachium guairarepanensis* Señaris 2001 | | | |  | |  | | X | |  | |  |  |  |  |
| *Hyalinobatrachium* sp. | | | |  | |  | | X | |  | |  |  |  |  |
| *Ikakogi tayrona* (Ruiz-Carranza & Lynch 1991) | | | | X | |  | |  | |  | |  |  |  |  |
| *Vitreorana castroviejoi* (Ayarzagüena & Señaris 1997) | | | |  | |  | |  | |  | |  | X |  |  |
| **FAMILY LEPTODACTYLIDAE** | | | |  | |  | |  | |  | |  |  |  |  |
| *Leptodactylus* sp. | | | |  | |  | |  | |  | |  | X |  |  |
| **FAMILY HEMIPHRACTIDAE** | | | |  | |  | |  | |  | |  |  |  |  |
| *Cryptobatrachus boulengeri* Ruthven 1916 | | | | X | |  | |  | |  | |  |  |  |  |
| *Cryptobatrachus ruthveni* Lynch 2008 | | | | X | |  | |  | |  | |  |  |  |  |
| *Flectonotus pygmaeus* (Boettger 1893) | | | |  | | X | | X | |  | |  |  |  |  |
| *Gastrotheca ovifera* (Lichtenstein & Weinland 1854) | | | |  | |  | | X | |  | |  |  |  |  |
| *Gastrotheca walkeri* Duellman 1980 | | | |  | |  | | X | |  | |  |  |  |  |
| *Gastrotheca williamsoni* Gaige 1922 | | | |  | |  | | X | |  | |  |  |  |  |
| *Gastrotheca* sp. | | | |  | |  | |  | |  | |  | X |  |  |
| **FAMILY STRABOMANTIDAE** | | | |  | |  | |  | |  | |  |  |  |  |
| *Geobatrachus walkeri* Ruthven 1915 | | | | X | |  | |  | |  | |  |  |  |  |
| *Pristimantis anotis* (Walker & Test 1955) | | | |  | |  | | X | |  | |  |  |  |  |
| *Pristimantis bicumulus* (Peters 1864) | | | |  | |  | | X | |  | |  |  |  |  |
| *Pristimantis geminus* Kaiser et al. 2015 | | | |  | |  | |  | |  | |  | X |  |  |
| *Pristimantis hoogmoedi* Kaiser et al. 2015 | | | |  | |  | |  | |  | |  | X |  |  |
| *Pristimantis longicorpus* Kaiser et al. 2015 | | | |  | |  | |  | |  | |  | X |  |  |
| *Pristimantis nubisilva* Kaiser et al. 2015 | | | |  | |  | |  | |  | |  | X |  |  |
| *Pristimantis pariagnomus* Kaiser et al. 2015 | | | |  | |  | |  | |  | |  | X |  |  |
| *Pristimantis reticulatus* (Walker & Test 1955) | | | |  | |  | | X | |  | |  |  |  |  |
| *Pristimantis riveroi* (Lynch & La Marca 1993) | | | |  | |  | | X | |  | |  |  |  |  |
| *Pristimantis rozei* (Rivero 1961) | | | |  | |  | | X | |  | |  |  |  |  |
| *Pristimantis stenodiscus* (Walker & Test 1955) | | | |  | |  | | X | |  | |  |  |  |  |
| *Pristimantis incertus* (Lutz 1927) | | | |  | |  | | X | |  | |  |  |  |  |
| *Pristimantis turpinorum* (Hardy 21) | | | |  | |  | |  | |  | |  |  |  | X |
| *Pristimantis turimiquirensis* (Rivero 1961) | | | |  | |  | |  | |  | | X |  |  |  |
| *Pristimantis carmelitae* (Ruthven 1922) | | | | X | |  | |  | |  | |  |  |  |  |
| *Pristimantis cristinae* (Lynch & Ruiz-Carranza 1985) | | | | X | |  | |  | |  | |  |  |  |  |
| *Pristimantis delicatus* (Ruthven 1917) | | | | X | |  | |  | |  | |  |  |  |  |
| *Pristimantis insignitus* (Ruthven 1917) | | | | X | |  | |  | |  | |  |  |  |  |
| *Pristimantis megalops* (Ruthven 1917) | | | | X | |  | |  | |  | |  |  |  |  |
| *Pristimantis ruthveni* (Lynch & Ruiz-Carranza 1985) | | | | X | |  | |  | |  | |  |  |  |  |
| *Pristimantis sanctaemartae* (Ruthven 1917) | | | | X | |  | |  | |  | |  |  |  |  |
| *Pristimantis tayrona* (Lynch & Ruiz-Carranza 1985) | | | | X | |  | |  | |  | |  |  |  |  |
| *Pristimantis w-nigrum* (Boettger 1892) | | | | X | |  | |  | |  | |  |  |  |  |
| *Pristimantis* sp. | | | |  | |  | |  | |  | | X |  |  |  |
| *Strabomantis biporcatus* Peters 1863 | | | |  | |  | | X | |  | |  | X |  |  |
| **FAMILY PHYLLOMEDUSIDAE** | | | |  | |  | |  | |  | |  |  |  |  |
| *Agalychnis medinae* (Funkhouser 1962) | | | |  | |  | | X | |  | |  |  |  |  |
| **FAMILY HYLIDAE** | | | |  | |  | |  | |  | |  |  |  |  |
| *Dendropsophus battersbyi* (Rivero 1961) | | | |  | |  | | X | |  | |  |  |  |  |
| *Dendropsophus yaracuyanus* (Mijares-Urrutia & Rivero 2000) | | | |  | |  | | X | |  | |  |  |  |  |
| *Phytotriades auratus* (Boulenger 1917) | | | |  | |  | |  | |  | |  | X | X |  |
| **ORDER CAUDATA** | | | |  | |  | |  | |  | |  |  |  |  |
| **FAMILY PLETHODONTIDAE** | | | |  | |  | |  | |  | |  |  |  |  |
| *Bolitoglossa borburata* Trapido 1942 | | | |  | |  | | X | |  | |  |  |  |  |
| *Bolitoglossa savagei* (Brame & Wake 1963) | | | | X | |  | |  | |  | |  |  |  |  |
| *Bolitoglossa* sp. | | | |  | | X | |  | |  | |  |  |  |  |
|  | | | |  | |  | |  | |  | |  |  |  |  |
| **TOTAL** | | | | **21** | | **3** | | **27** | |  | | **4** | **12** | **1** | **1** |
|  | | | |  | |  | |  | |  | |  |  |  |  |
|  | | | |  | |  | |  | |  | |  |  |  |  |
|  | | | | **SNSM** | | **SSL** | | **CCR** | | **IMA** | | **TUR** | **PR** | **TRI** | **TOB** |
|  | | | |  | |  | |  | |  | |  |  |  |  |
|  | | | |  | |  | |  | |  | |  |  |  |  |
| **CLASS REPTILIA** | | | |  | |  | |  | |  | |  |  |  |  |
| **ORDER SQUAMATA** | | | |  | |  | |  | |  | |  |  |  |  |
| **FAMILY DACTYLOIDAE** | | | |  | |  | |  | |  | |  |  |  |  |
| *Anolis menta* Ayala, Harris & Williams 1984 | | | | X | |  | |  | |  | |  |  |  |  |
| *Anolis paravertebralis* Bernal-Carlo & Roze 2005 | | | | X | |  | |  | |  | |  |  |  |  |
| *Anolis santamartae* Williams 1982 | | | | X | |  | |  | |  | |  |  |  |  |
| *Anolis solitarius* Ruthven 1916 | | | | X | |  | |  | |  | |  |  |  |  |
| *Anolis squamulatus* Peters 1863 | | | |  | |  | | X | |  | |  |  |  |  |
| *Anolis tigrinus* Peters 1863 | | | |  | | X | | X | |  | | X | X |  |  |
| *Anolis umbrivagus* Bernal-Carlo & Roze 2005 | | | | X | |  | |  | |  | |  |  |  |  |
| *Anolis* sp. | | | |  | |  | |  | |  | |  |  | X | X |
| **FAMILY POLYCHROTIDAE** | | | |  | |  | |  | |  | |  |  |  |  |
| *Polychrus gutturosus* Berthold 1846 | | | | X | |  | |  | |  | |  |  |  |  |
| **FAMILY SPHAERODACTYLIDAE** | | | |  | |  | |  | |  | |  |  |  |  |
| *Gonatodes seigliei* Donoso-Barros 1966 | | | |  | |  | |  | |  | | X |  |  |  |
| *Gonatodes taniae* Roze 1963 | | | |  | |  | | X | |  | |  |  |  |  |
| *Gonatodes machelae* Rivero-Blanco & Schargel 2020 | | | |  | |  | |  | | X | |  |  |  |  |
| *Gonatodes* sp. | | | |  | |  | |  | |  | | X | X |  |  |
| *Pseudogonatodes furvus* Ruthven1915 | | | | X | |  | |  | |  | |  |  |  |  |
| *Pseudogonatodes manessi* Avila-Pires & Hoogmoed 2000 | | | |  | |  | | X | |  | |  |  |  |  |
| *Pseudogonatodes* sp. | | | |  | |  | |  | |  | |  | X |  |  |
| **FAMILY GYMNOPHTHALMIDAE** | | | |  | |  | |  | |  | |  |  |  |  |
| *Anadia altaserrania* Harris & Ayala 1987 | | | | X | |  | |  | |  | |  |  |  |  |
| *Anadia blakei* Schmidt 1932 | | | |  | |  | |  | |  | | X | X |  |  |
| *Anadia marmorata* (Gray 1846) | | | |  | |  | | X | |  | |  |  |  |  |
| *Anadia pariaensis* Rivas, La Marca & Oliveros 1999 | | | |  | |  | |  | |  | |  | X |  |  |
| *Anadia pulchella* Ruthven 1926 | | | | X | |  | |  | |  | |  |  |  |  |
| *Bachia trinitatis* (Barbour 1914) | | | |  | |  | |  | | X | |  | X | X |  |
| *Bachia whitei* Murphy et al. 2019 | | | |  | |  | |  | |  | |  |  |  | X |
| *Euspondylus acutirostris* (Peters 1862) | | | |  | | X | | X | |  | |  |  |  |  |
| *Euspondylus monsfumus* Mijares-Urrutia et al. 2001 | | | |  | |  | |  | |  | |  | X |  |  |
| *Oreosaurus achlyens* (Uzzell 1958) | | | |  | |  | | X | |  | |  |  |  |  |
| *Oreosaurus luctuosus* (Peters 1862) | | | |  | |  | | X | |  | |  |  |  |  |
| *Oreosaurus rhodogaster* (Rivas et al. 2005) | | | |  | |  | |  | |  | |  | X |  |  |
| *Oreosaurus serranus* Sánchez-Pacheco et al. 2017 | | | | X | |  | |  | |  | |  |  |  |  |
| *Oreosaurus shrevei* (Parker 1935) | | | |  | |  | |  | |  | |  |  | X |  |
| *Oreosaurus* sp. | | | |  | |  | |  | |  | | X |  |  |  |
| **FAMILY ALOPOGLOSSIDAE** | | | |  | |  | |  | |  | |  |  |  |  |
| *Ptychoglossus romaleos* Harris 1994 | | | | X | |  | |  | |  | |  |  |  |  |
| **FAMILY MABUYIDAE** | | | |  | |  | |  | |  | |  |  |  |  |
| *Copeoglossum margaritae* (Hedges & Conn 2012) | | | |  | |  | |  | | X | |  |  |  |  |
| *Orosaura nebulosylvestris* (Miralles et al. 2009) | | | |  | |  | | X | |  | |  |  |  |  |
| *Panopa croizati* (Horton 1973) | | | |  | |  | |  | |  | | X |  |  |  |
| **FAMILY COLUBRIDAE** | | | |  | |  | |  | |  | |  |  |  |  |
| *Chironius monticola* Roze 1952 | | | |  | | X | | X | |  | |  |  |  |  |
| *Chironius septentrionalis* (Dixon, Wiest & Cei 1993) | | | |  | |  | | X | |  | |  |  |  |  |
| *Dendrophidion percarinatum* (Cope 1893) | | | | X | |  | |  | |  | |  |  |  |  |
| *Dendrophidion nuchale* (Peters 1864) | | | |  | |  | | X | |  | |  |  |  |  |
| *Lampropeltis micropholis* (Cope 1861) | | | | X | |  | |  | |  | |  |  |  |  |
| **FAMILY DIPSADIDAE** | | | |  | |  | |  | |  | |  |  |  |  |
| *Atractus fuliginosus* (Hallowell 1845) | | | |  | |  | | X | |  | |  |  |  | X |
| *Atractus lancinii* Roze 1961 | | | |  | |  | | X | |  | |  |  |  |  |
| *Atractus matthewi* Markezich & Barrio-Amorós 2004 | | | |  | |  | |  | |  | | X |  |  |  |
| *Atractus sanctaemartae* Dunn 1946 | | | | X | |  | |  | |  | |  |  |  |  |
| *Atractus vittatus* Boulenger 1894 | | | |  | |  | | X | |  | |  |  |  |  |
| *Dipsas praeornata* Werner 1909 | | | |  | |  | | X | |  | |  |  |  |  |
| *Dipsas variegata* (Duméril, Bibron & Duméril 1854) | | | |  | |  | | X | |  | | X |  |  |  |
| *Erythrolamprus epinephellus* (Cope 1862) | | | | X | |  | |  | |  | |  |  |  |  |
| *Erythrolamprus mertensi* (Roze 1964) | | | |  | | X | | X | |  | |  |  |  |  |
| *Erythrolamprus pseudoreginae* Murphy et al. 2019 | | | |  | |  | |  | |  | |  |  |  | X |
| *Erythrolamprus williamsi* (Roze 1958) | | | |  | |  | | X | |  | |  |  |  |  |
| *Erythrolamprus zweifeli* (Roze 1959) | | | |  | | X | | X | |  | | X | X |  |  |
| *Taeniophallus nebularis* Schargel et al. 2005 | | | |  | |  | |  | |  | |  | X |  |  |
| *Thamnodynastes ramonriveroi* Manzanilla & Sánchez 2005 | | | |  | |  | |  | |  | | X |  |  |  |
| *Urotheca multilineata* (Peters 1859) | | | |  | |  | | X | |  | |  |  |  |  |
| **FAMILY ELAPIDAE** | | | |  | |  | |  | |  | |  |  |  |  |
| *Micrurus mipartitus* (Duméril et al. 1854) | | | | X | | X | | X | |  | |  |  |  |  |
| **FAMILY VIPERIDAE** | | | |  | |  | |  | |  | |  |  |  |  |
| *Bothrops medusa* (Sternfeld 1920) | | | |  | |  | | X | |  | |  |  |  |  |
| *Bothrops venezuelensis* Sandner-Montilla 1952 | | | |  | |  | | X | |  | | X | X |  |  |
| **FAMILY ANOMALEPIDIDAE** | | | |  | |  | |  | |  | |  |  |  |  |
| *Helminthophis flavoterminatus* (Peters 1857) | | | |  | |  | | X | |  | |  |  | X |  |
|  | | | |  | |  | |  | |  | |  |  |  |  |
| **TOTAL** | | | | **16** | | **6** | | **25** | | **3** | | **11** | **11** | **4** | **4** |

The endemic species listed here belongs only to those found from the middle and high elevation within the study area. Most of them are exclusives of a single mountain chain, although with few exceptions, some taxa (p.e. *Phytotriades auratus*), also occurs in more than one system. The lowland endemic species are not considered (p.e. *Allobates pittieri* or *Flectonotus fitzgeraldi*), in spite that some of them also occurs in highest elevations. The elevations considered here are: SNSM (1000-4500 m asl), SSL (450-1500 m asl.), CCR (Serranía del Litoral: North side: 600-1650 m asl; South side: 900-1650 m asl. Serranía del Interior: 900-1700 m asl: Sierra de Aroa: 600-1950 m asl), IMA (refers to the Cerro Copey West side: 500-930 m asl; East side: 250-930 m asl), TUR (900-2400 m asl), PR (Serranía de Paria: 500-1250 m asl: Campeare: 750-950 m asl; Cerbatana 600-1000 m asl), TRI (550-940 m asl.), TOB (300-540 m asl.).
