# Supplementary material for: Biogeographical patterns of amphibians and reptiles in the northernmost coastal montane complex of South America
Source: PLoS One. 2021 Mar 4;16(3):e0246829. doi: 10.1371/journal.pone.0246829 (PMC7932178; doi:10.1371/journal.pone.0246829)
Supplement: S5 Table — (DOCX) [file pone.0246829.s005.docx]

**S7 Table**

**Polygons calculated for areas above 200 m in altitude.**

|  |  |  |  |  |
| --- | --- | --- | --- | --- |
|  | **Shape Length (km)** | **Area (km²)** | **System Name** | **Montain Name** |
|  |  |  |  |  |
| 1 | 96 | 123 | Tobago | Tobago Main Ridge |
|  |  |  |  |  |
| 2 | 310 | 650 | Trinidad | Trinidad Northern Range |
|  |  |  |  |  |
| 3 | 385 | 1124 | Paria Range | Serraía de Paria |
|  |  |  |  |  |
| 4 | 68 | 222 |  | Cerro Campeare |
|  |  |  |  |  |
| 5 | 112 | 366 |  | Cerro La Cerbatana |
|  |  |  |  |  |
|  | 565 | 1712 | Total Paria Range |  |
|  |  |  |  |  |
| 6 | 180 | 231 | Isla de Margarita | Cerros del Norte |
|  |  |  |  | Cerro Guayamurí |
|  |  |  |  | Cerro Mata Siete |
|  |  |  |  | Cerro Copey* |
|  |  |  |  | Cerro de Macanao |
|  |  |  |  |  |
| 7 | 555 | 8125 | Macizo del Turimiquire |  |
|  |  |  |  |  |
| 8 | 1447 | 26764 | Central Coastal Range | Serranía del Litoral |
|  |  |  |  | Serranía del Interior |
|  |  |  |  | Macizo de Nirgua |
|  |  |  |  |  |
| 9 | 473 | 3526 |  | Sierra de Aroa |
|  |  |  |  |  |
|  | 1920 | 30190 | Total Central Costal Range |  |
|  |  |  |  |  |
| 10 | 292 | 1112 | Sierra de San Luis |  |
|  |  |  |  |  |
| 11 | 778 | 12230 | Sierra Nevada de Santa Marta |  |

**Area calculated for altitude according to mountain vegetation**

|  | **Shape Length (km)** | **Area (km²)** | **System Name** | **Montain Name** |
| --- | --- | --- | --- | --- |
|  |  |  |  |  |
|  |  |  |  | Tobago Main Ridge |
|  |  |  |  |  |
| 1 | 84040,17754 | 55,83 | Tobago | TOB-1 |
| 2 | 4452,861206 | 0,61 |  | TOB-2 |
| 3 | 890,6213845 | 0,04 |  | TOB-3 |
|  |  |  |  |  |
|  | 89383,66013 | 56,48 | Total Tobago |  |
|  |  |  |  |  |
|  |  |  |  |  |
|  |  |  |  | Trinidad Northern Range |
|  |  |  |  |  |
| 4 | 3037,779414 | 0,42 | Trinidad | TRI-1 |
| 5 | 1824,489376 | 0,11 |  | TRI-2 |
| 6 | 3540,536467 | 0,51 |  | TRI-3 |
| 7 | 23215,35266 | 7,45 |  | TRI-4 |
| 8 | 2316,225421 | 0,27 |  | TRI-5 |
| 9 | 4626,467942 | 0,61 |  | TRI-6 |
| 10 | 5544,931045 | 0,83 |  | TRI-7 |
| 11 | 1428,730478 | 0,11 |  | TRI-8 |
| 12 | 738,3644516 | 0,04 |  | TRI-9 |
| 13 | 68756,85512 | 32,56 |  | TRI-10 |
| 14 | 1035,608405 | 0,07 |  | TRI-11 |
| 15 | 1055,017207 | 0,06 |  | TRI-12 |
| 16 | 785,911146 | 0,04 |  | TRI-13 |
| 17 | 18853,48869 | 3,31 |  | TRI-14 |
| 18 | 1571,921135 | 0,08 |  | TRI-15 |
| 19 | 4323,499999 | 0,59 |  | TRI-16 |
| 20 | 2368,211184 | 0,22 |  | TRI-17 |
|  |  |  |  |  |
|  | 145023,3901 | 47,28 | Total Trinidad |  |
|  |  |  |  |  |
|  |  |  |  |  |
|  |  |  | Paria Range | Serranía de Paria |
|  |  |  |  |  |
| 21 | 84909,49755 | 54,27 |  | PAR-1 |
| 22 | 3015,95971 | 0,31 |  | PAR-2 |
| 23 | 3952,831097 | 0,54 |  | PAR-3 |
| 24 | 19610,16132 | 6,5 |  | PAR-4 |
| 25 | 404985,4376 | 293,74 |  | PAR-5 |
| 26 | 10302,10477 | 1,56 |  | PAR-6 |
|  |  |  |  |  |
|  |  |  |  | Cerro Campeare |
|  |  |  |  |  |
| 27 | 12942,56262 | 3,98 |  | PR 1 |
|  |  |  |  |  |
|  |  |  |  | Cerro La Cerbatana |
|  |  |  |  |  |
| 28 | 37370,5128 | 16,43 |  | PR 2 |
|  |  |  |  |  |
|  | 577089,0675 | 377,33 | Total Paria Range |  |
|  |  |  |  |  |
|  |  |  |  |  |
| 29 | 4663,84917 | 0,7 | Isla de Margarita | IMAw-1 |
| 30 | 1848,510755 | 0,19 |  | IMAw-2 |
| 31 | 10402,83769 | 2,19 |  | IMAw-3 |
| 32 | 1256,783629 | 0,11 |  | IMAw-4 |
| 33 | 46860,3143 | 25,63 |  | IMAe-1 |
| 34 | 12413,20603 | 3,99 |  | IMAe-2 |
| 35 | 13612,40295 | 2,28 |  | IMAe-3 |
| 36 | 14058,82989 | 3,18 |  | IMAe-4 |
| 37 | 11039,13632 | 1,86 |  | IMAe-5 |
| 38 | 16842,59768 | 3,94 |  | IMAe-6 |
| 39 | 6598,590057 | 1,14 |  | IMAe-7 |
|  |  |  |  |  |
|  | 139597,0585 | 45,21 | Total Isla de Margarita |  |
|  |  |  |  |  |
|  |  |  |  |  |
| 40 | 11810,56952 | 3,15 | Turimiquire Massif | TUR-w-4 |
| 41 | 692003,5339 | 1268,31 |  | TUR-w-1 |
| 42 | 344772,8826 | 868,98 |  | TUR-e-1 |
| 43 | 47174,36652 | 49,3 |  | TUR-w-2 |
| 44 | 41770,77905 | 33,88 |  | TUR-w-3 |
| 45 | 16713,68469 | 7,29 |  | TUR-e-2 |
|  |  |  |  |  |
|  | 1154245,816 | 2230,91 | Total Turimiquire Massif |  |
|  |  |  |  |  |
|  |  |  |  |  |
|  |  |  | Central Coastal Range | Serrania del Litoral |
|  |  |  |  |  |
| 46 | 839256,0517 | 3393,74 |  | CCR-sl |
|  |  |  |  |  |
|  |  |  |  | Serranía del Interior (Include Macizo de Nirgua) |
|  |  |  |  |  |
| 47 | 340493,7658 | 631 |  | CCR-si-1 |
| 48 | 28821,48166 | 44,27 |  | CCR-si-2 |
| 49 | 241446,0457 | 617,24 |  | CCR-si-3 |
| 50 | 123087,7528 | 102,24 |  | CCR-si-4 |
| 51 | 220128,6093 | 265,74 |  | CCR-si-5 |
| 52 | 96370,54037 | 68,66 |  | CCR-si-6 |
| 53 | 87840,80255 | 93,52 |  | CCR-si-7 |
| 54 | 83007,70544 | 72,94 |  | CCR-si-8 |
| 55 | 23429,10864 | 13,95 |  | CCR-si-9 |
| 56 | 277230,4862 | 326,68 |  | CCR-si-10 |
| 57 | 116454,6237 | 123,97 |  | CCR-si-11 |
| 58 | 94272,23056 | 70,55 |  | CCR-si-12 |
| 59 | 69655,10047 | 93,43 |  | CCR-si-13 |
| 60 | 103408,7125 | 137,04 |  | CCR-si-14 |
| 61 | 24474,26288 | 16,51 |  | CCR-si-15 |
| 62 | 36210,03934 | 21,56 |  | CCR-si-16 |
| 63 | 16199,19808 | 10,34 |  | CCR-si-17 |
| 64 | 18310,50213 | 8,35 |  | CCR-si-18 |
|  |  |  |  |  |
|  |  |  |  | Sierra de Aroa |
|  |  |  |  |  |
| 65 | 200279,442 | 914,72 |  | CCR-sa |
|  |  |  |  |  |
|  | 3040376,462 | 7026,45 | Total Central Coastal Range |  |
|  |  |  |  |  |
|  |  |  |  |  |
| 66 | 222238,2207 | 907,89 | Sierra de San Luis | SSL-1 |
|  |  |  |  |  |
|  |  |  |  |  |
| 67 | 1086548,805 | 6635,42 | Sierra Nevada de Santa Marta | SNSM-1000 |
| 68 | 27268,08869 | 12,13 |  | SNSM4500-1 |
| 69 | 167486,5391 | 152,53 |  | SNSM4500-2 |
| 70 | 3844,990318 | 0,45 |  | SNSM4500-3 |
| 71 | 10340,37798 | 1,72 |  | SNSM4500-4 |
| 72 | 15085,62977 | 4,37 |  | SNSM4500-5 |
|  |  |  |  |  |
|  | 1310574,431 | 6806,62 | Total Sierra Nevada de Santa Marta |  |
|  |  |  |  |  |
