# Supplementary material for: Biogeographical patterns of amphibians and reptiles in the northernmost coastal montane complex of South America
Source: PLoS One. 2021 Mar 4;16(3):e0246829. doi: 10.1371/journal.pone.0246829 (PMC7932178; doi:10.1371/journal.pone.0246829)
Supplement: S6 Table — (DOCX) [file pone.0246829.s006.docx]

**S8 Table**

Mountain systems and altitude delimitations and bibliography references used for all systems. SNSM (Sierra Nevada de Santa Marta, Colombia), SSL (Sierra de San Luis), CCR (Central Coastal Range), IMA (Isla de Margarita), TUR (Turimiquire Massif), PR (Paria Range), TRI (island of Trinidad), TOB (island of Tobago).

**References**

Ataroff S, M. Selvas y bosques de montaña. pp. 762–810. In: M. Aguilera, A. Azócar & E. González Jiménez (eds.). Biodiversidad en Venezuela. Tomo II. Fundación Polar. Ministerio de Ciencia y Tecnología. 2003.

Beard JS. The natural vegetation of the island of Tobago, British West Indies. Ecol Monogr. 1944;14:136-63.

Beard JS. The natural vegetation of Trinidad. The natural vegetation of Trinidad. Oxford at the Clarendon Press. 152 pp. 1946.

García F, Aular L, Camargo-Siliet, E, Mujica Y. Murciélagos de la Sierra de Aroa, Estado Yaracuy, Venezuela. Memoria Fund La Salle de Cienc Nat. 2012 (“2010”);173-174:135-154.

Heald WF. The Chiricahuas Sky Island. Bantlin Pub., Tucson, Az. 1993.

Helmer EH, Ruzycki TS, Benner J, Voggesser SM, Scobie BP, Park C, Fanning DW. Ramnarine S. Detailed maps of tropical forest types are within reach: Forest tree communities for Trinidad and Tobago mapped with multiseason Landsat and multiseason fine-resolution imagery. Forest Evol Manag. 2012;279:147-166.

Hernández Camacho, J. & Sánchez Paéz, H. 1992. Biomas terrestres de Colombia. pp. 153−169. In: Hallfter (compilador). La diversidad biológica de Iberoamérica. Programa Iberoamericano de Ciencia y Tecnología para el Desarrollo Instituto de Ecología, A.C. Secretaria de Desarrollo Social. México. 1992.

Hoyos J. Flora de la Isla Margarita, Venezuela. Soc Fund La Salle Cienc Nat Monogr. 1985;34:1-927.

PROSIERRA (Fundación Pro-Sierra Nevada de Santa Marta). Evaluación Ecológica Rápida de la Sierra Nevada de Santa Marta. Definición de Áreas Criticas para la Conservación de la Sierra Nevada de Santa Marta, Colombia. Ministerio del Medio Ambiente, UAESPPNN, The Nature Conservancy, USAID, Embajada de Japón. 134 pp. 1998.

Silva Ríos A. Sucesión secundaria después de actividades agrícolas en el bosque semideciduo, península de Paria, Venezuela: aplicación para un programa de restauración. pp:73−96. In: F. Herrera & I. Herrera (ed.). La Restauración Ecológica en Venezuela: Fundamentos y Experiencias. Ediciones IVIC, Instituto Venezolano de Investigaciones Científicas, Caracas. 2011.

Steyermark J, Agostini G. Exploración botánica del Cerro Patao y zonas adyacentes a Puerto Hierro, en la Península de Paria, edo. Sucre. Acta Bot Venez. 1966;2:7-80.

Sugden AM. The montane vegetation and flora of Margarita island, Venezuela. J Arnold Arbor. 1986;67:187-232.

van der Hammen T, Ruiz-Carranza P. (eds.). La Sierra Nevada de Santa Marta Transecto Buritaca- La Cumbre. Estudios de ecosistemas tropandinos 2. J. Cramer, Berlín-Stuttgart. 1984.
