# Supplementary material for: Biogeographical patterns of amphibians and reptiles in the northernmost coastal montane complex of South America
Source: PLoS One. 2021 Mar 4;16(3):e0246829. doi: 10.1371/journal.pone.0246829 (PMC7932178; doi:10.1371/journal.pone.0246829)
Supplement: S1 Text — (DOCX) [file pone.0246829.s007.docx]

**S2 Text**

Specimens examined. Some of these specimens have already been mentioned from the literature. Others however, increase the known distribution range of some of the species (see Supporting Information 4). Voucher specimens are deposited in the Museo de Biología, La Universidad del Zulia, Maracaibo (MBLUZ); Museo de la Estación Biológica de Rancho Grande, Maracay (EBRG); Museo de Ciencias Naturales, Caracas (MCNC); Museo de Biología, Universidad Central de Venezuela (MBUCV); Museo de Historia Natural La Salle (MHNLS), Caracas; American Museum of Natural History, New York (AMNH); Amphibian and Reptile Diversity Research Center, The University of Texas at Arlington, Arlington (UTA). Similarly, some of these specimens are support for undescribed species considered in this study.

AMPHIBIA

**Aromobatidae**

*Allobates caribe.* SUCRE: southern slope of Cerro Humo, Península de Paria,

1050 m (MHNLS 17462-63, 17498, type series).

*Allobates mandelorum.* ANZOATEGUI: Serranía de Turimiquire, cumbre Cerro La Laguna, 2200 m (EBRG 3697, 3699).

*Mannophryne riveroi.* SUCRE: Quebrada Las Melenas, 773 m (MBLUZ 450, MHNLS 16172–16176).

*Mannophryne venezuelensis.* SUCRE: Quebrada Seca, Cerro Campeare (EBRG 7341-42). Rio Santa Isabel, Vertiente Norte de la Península de Paria (MBLUZ 410-412). Cachipal, Península de Paria (MBLUZ 453-460). La Toma, La Cerbatana (MBLUZ).

**Centrolenidae**

*Celsiella vozmedianoi.* SUCRE: Quebrada Las Melenas, vertiente Sur de Cerro Humo, Península de Paria (MHNLS 17877).

*Hyalinobatrachium orientale.* SUCRE: Quebrada Las Melenas, vertiente Sur de Cerro Humo, Península de Paria (MHNLS 17878–17879, MBLUZ). Camino entre Macuro y Los Chorros, Península de Paria (MHNLS 16443–16445, 16647–16651).

*Vitreorana castroviejoi.* SUCRE: camino entre Macuro y Los Chorros, Península de Paria (MBLUZ 379–380). Cabeceras del Rio Tacarigua, vertiente norte de la Península de Paria (MBLUZ 422).

**Strabomantidae**

*Craugastor biporcatus*. SUCRE: Península de Paria, carretera cerro Humo, vía Las Melenas (EBRG 6424).

*Pristimantis* *nubisilva.* SUCRE: Vertiente Sur de Cerro Humo, Península de Paria, 800 m (MBLUZ 404–409). La Cerbatana (EBRG 7338). Cerro Campeare (MBLUZ 426). Cachipal, Península de Paria, 800 m (MBLUZ 446).

*Pristimantis* *pariagnomus.* SUCRE: Vertiente Sur de Cerro Humo, Península de Paria, 800 m (MBLUZ 427–428). Quebrada Las Melenas, Península de Paria, 773 m (MBLUZ 461).

**Hemiphractidae**

*Flectonotus fitzgeraldi*. MONAGAS: La Margarita, 1200 m (EBRG 7034-35): SUCRE: Mauraco, N del Pilar (EBRG 519-520). Marauquito, Península de Paria, 400 m (MHNLS 10859). Vertiente Sur de Cerro Humo, Península de Paria, 800 m (MBLUZ 0395, 0448). Alrededores de las Melenas, Península de Paria (MHNLS 15741, 16187). Camino desde Macuro a los Chorros, Península de Paria, 500 m (MHNLS 16199-17201). Uquire, vertiente Norte de la Península de Paria, 150 m (EBRG 2585). Cachipal, Península de Paria, 800 m (MBLUZ 447). Cerro La Cerbatana, 800 m (EBRG 7336–7337). Cerro Campeare, 800 m (EBRG 7343–7346).

**Hylidae**

*Boana boans.* SUCRE: Santa Isabel, vertiente Norte de la Península de Paria (MBLUZ 397).

*Phyllomedusa trinitatis*. SUCRE: Campeare (MBLUZ 431, tadpoles).

*Phytotriades auratus*. SUCRE: Cerro Humo, Serranía de Paria (MBLUZ 393–394, 413).

*Scinax ruber*. SUCRE: Cachipal, Península de Paria, 800 m (MBLUZ 451-452).

**Leptodactylidae**

*Leptodactylus* *fuscus*. SUCRE: Cachipal, Península de Paria, 800 m (MBLUZ 449).

*Leptodactylus turimiquensis.* MONAGAS: Distrito Caripe, El Caliche, Juasjuillar (EBRG 2085).

*Leptodactylus* sp. SUCRE: La Cerbatana (MBLUZ 396, EBRG 7339–7340).

**Plethodontidae**

*Bolitoglossa* *borburata*. ARAGUA: Rancho Grande (EBRG 1048, 4077). YARACUY: Las Lajas, sector Carabobo, Parque Nacional Yurubí (MBLUZ 391). Río Carabobo, Parque Nacional Yurubí (MBLUZ 392).

*Bolitoglossa* sp. FALCÓN: Curimagua, Sierra San Luis (EBRG 4056, 4068–4070).

**Caecilidae**

*Caecilia flavopunctata*. YARACUY: Albarico (MBUCV 5358, Holotype). Sector Mayorica, Municipio San Felipe (EBRG 5780). Hacienda el Jaguar, 15 km NE Aroa, Sierra de Bobare, 600 m (EBRG 3343).

REPTILIA

**Kinosternidae**

*Kinosternon scorpioides*. FALCÓN: Agropecuaria El Haitón, Sierra de San Luis, 297 m (EBRG 4035).

**Sphaerodactylidae**

*Gonatodes ceciliae.* SUCRE: La Toma, La Cerbatana (MBLUZ 1254). Cerro Campeare (MBLUZ 1427). Cachipal, Península de Paria, 800 m (UTA uncatalogued). Las Melenas, Península de Paria, 700 m (MHNLS 15541, 15727–15729, 17087). Carretera Güiria-Macuro, Península de Paria, 20 m (MHNLS 16695, 15725–15726). Balneario Río Guayana, carretera Carupano-Güiria, 50 m (MHNLS 18047–18049).

.

*Gonatodes seiglei.* MONAGAS: alrededores de la cueva del Guácharo, Parque Nacional Guácharo (MHNLS 16716–17, 16721–23).

*Gonatodes* sp. SUCRE: Cerro Campeare (MBLUZ 1413–1615). La Cerbatana (MBLUZ 1255–56, 1409). Maturincito, La Cerbatana (MBLUZ 1257–58). Cachipal, Península de Paria, 800 m (UTA uncatalogued).

*Pseudogonatodes* sp. SUCRE: Camino entre Macuro y Los Chorros, 500 m (MBLUZ 1292–1294).

**Phyllodactylidae**

*Thecadactylus rapicauda*. SUCRE: Macuro, Península de Paria (MHNLS 11646).

**Gekkonidae**

*Hemidactylus mabouia.* SUCRE: Cerro Campeare, 950 m (MBLUZ).

**Gymnophthalmidae**

*Anadia blakei*. VENEZUELA: Sucre: Cerro Humo, Península de Paria (EBRG 2746).

*Anadia marmorata*. ARAGUA: Rancho Grande (MHNLS 14473–14474). MIRANDA: carretera Panamericana, km 11, IVIC, Centro de Ecología (EBRG 5919). YARACUY: Los Bacos, Sierra de Aroa, 1300 m (EBRG 3211).

*Anadia pariaensis*. SUCRE: Las Melenas, Península de Paria, 700 m (MBLUZ 930). Vertiente Sur de Cerro Humo, Serranía de Paria, 800 m (EBRG 1307). Rio Tacarigua, Parque Nacional Península de Paria, 450 m (EBRG 2742, holotype).

*Anadia steyeri*. FALCÓN: Cataratas de Hueque, Sierra de San Luis, municipio Petit, (MBLUZ 397); Parque Nacional Juan Crisóstomo Falcón (EBRG 4335). Cerro Misión, Este del estado Falcón, a 20 km del suroeste del pueblo de Sanare, 400 m (MBLUZ 762). Miranda: 25 km north of Altagracia, Parque Nacional Guatopo, guest house, 700 m (TCWC 59856). YARACUY: Hacienda el Zinc, Sierra de Aroa, 1220 m (EBRG 5995). Sector La Capilla, Sierra de Aroa, 1400 m (MBLUZ 1314). Hacienda el Jaguar, sierra de Bobare, Guasanillar, municipio Bolívar, 700 m (EBRG 2967). 2 km de los Ureros–Barlovento, Municipio Bolívar, sector Campochal, Sierra de Aroa, 600 m (MBLUZ 894).

*Bachia trinitatis*. SUCRE: Caserío de Roma, vertiente Sur de Cerro Humo, Serranía de Paria, ca. 800 m (MBLUZ 1298).

*Euspondylus acutirostris*. YARACUY: Sierra de Aroa (EBRG 5857). FALCÓN: Cerro Galicia, Sector La Soledad, Sierra de San Luís, 1200 m (MBLUZ 1232).

*Euspondylus monsfumus*. SUCRE: Cerro Humo, Serranía de Paria, 1240 m (MBLUZ 1287, 1346).

*Gymnophthalmus* sp. Avenida Maripérez con Avenida Boyacá, Estacionamiento del Edificio Fundación La Salle, Caracas, 1000 m (MBLUZ 1448).

*Oreosaurus achlyens.* ARAGUA: Rancho Grande, Parque Nacional Henri Pittier, 1100-1200 m (EBRG 5783, MBLUZ 901, MHNLS 16170, 3075, AMNH 137267–69, 137271–76, 137278–82); Tiara, 1000 m (MHNLS 1278). DISTRITO CAPITAL: Quebrada La Negra, embalse Agua Fría, Parque Nacional Macarao (MHNLS 17073–75). VARGAS: Las Llanadas, Hacienda El Limón, 1000 m (MHNLS 4924–25). Estado YARACUY: Hacienda La Guaquira, macizo de Nirgua, 1330 m (EBRG 5290).

*Oreosaurus luctuosus.* ARAGUA: Rancho Grande, Parque Nacional Henri Pittier, Aragua, 1100 m (EBRG 1706, MHNLS 1464, AMNH 137270, 137277). CARABOBO: Cerro La Copa, Montalbán, límite con Temerla, 1500-1600 m (EBRG 5748). YARACUY: Hacienda La Guaquira, macizo de Nirgua, 1330 m (EBRG 5288); Pico El Tigre, Parque Nacional Yurubí, sierra de Aroa, Yaracuy, 1800 m (EBRG 5851); same data as EBRG 5851, but collected at 1930 m (EBRG 5855).

*Oreosaurus rhodogaster.* SUCRE: Cerro La Cerbatana (MBLUZ 1410–11). Vertiente Sur de Cerro Humo, Península de Paria, 800 m (MBLUZ 1450)

*Oreosaurus* sp. ANZOATEGUI: Municipio Freites, Macizo del Turimiquire, Cerro El Guamal, 2150 m (EBRG 5962).

**Alopoglossidae**

*Ptychoglossus kugleri*. DISTRITO CAPITAL: línea del cortafuego, quebrada Maripérez, Parque Nacional El Ávila, 1050 m (MHNLS 11459). VARGAS: canales de Naiguatá, vertiente Norte del Parque Nacional El Ávila, 800 m (MHNLS 11800–11801). YARACUY: Municipio San Felipe, sector Bernabó, 350 m (MBLUZ 1445–1446).

**Dactyloidae**

*Anolis onca.* NUEVA ESPARTA: Salamanca, Cerro Matasiete, 305 m (MHNLS 584).

*Anolis planiceps.* SUCRE: Cerro Campeare (MBLUZ 1416). Vertiente Sur de Cerro Humo, Península de Paria (MBLUZ).

*Anolis tigrinus*. SUCRE: trail between Roma and Cerro Humo, serranía de Paria, 800 m (MBLUZ 1290).

**Tropiduridae**

*Tropidurus hispidus*. ARAGUA: Parque Nacional Henri Pittier (EBRG 4045). SUCRE: Las Melenas, Península de Paria, 700 m (EBRG 5423).

*Plica caribeana*. SUCRE: Cerro Humo, Península de Paria (MBLUZ 1344). Embalse Turimiquire, campamento base INOS, 300 m (EBRG 2267). Hacienda Solis, Los Mangos, Península de Paria (EBRG 2832). Los Mangos, Península de Paria (EBRG 2835). Uquire, Península de Paria (EBRG 2862).

**Scincidae**

*Copeoglossum aurae.* SUCRE: La Cerbatana (MBLUZ 1412).

*Copeoglossum* sp*.* ARAGUA: Quebrada margen derecha del Río San Miguel, Parque Nacional Henri Pittier (MHNLS 17080). FALCÓN: Pueblo Nuevo, municipio Petit (MBLUZ).

*Panopa croizati.* ANZOATEGUI: Cerro El Guamal, sector occidental del macizo del Turimiquire (MHNLS 17670–17675).

**Dipsadidae**

*Atractus fuliginosus*. Venezuela: Barinas: Barinitas (MCNC 4244). Cojedes: municipio San Carlos: Potrero Largo, Manrique (MHNLS 13834). Vargas: Planta Eléctrica de Naiguatá, 750-850 m (MCNC 3333, 4464, 4470, 4507, 4647, 5663, 5672, 7810).

*Atractus trilineatus*. SUCRE: Hacienda La Rinconada, 5 km N de Cumanacoa, 230 m (MCNC 7683–7692).

*Erythrolamprus mertensi*. ARAGUA: Carretera Maracay-Ocumare, km 20, 1100 m (EBRG 1934). Carretera Maracay-Ocumare, km 23.5 (EBRG 589). FALCÓN: Curimagua, Municipio Petit (MCNC 7705). YARACUY: Hacienda La Guaquira, cerro Zapatero (EBRG 5298)

*Erythrolamprus zweifeli*. MIRANDA: San Antonio de Los Altos (MCNC 1449)

*Ninia atrata.* DISTRITO CAPITAL: Bella Vista, Caracas, 900 m (MBLUZ 1296)*.* SUCRE: Cerro Campeare (MBLUZ 1426).

*Sibon nebulata.* SUCRE: Cerro Humo, Península de Paria (MBLUZ 1345),

*Siphlophis cervinus*. SUCRE: Cachipal, Península de Paria, 800 m (EBRG 6636).

*Thamnodynastes ramonriveroi*. ANZOATEGUI: Cerro El Guamal, sector occidental del macizo del Turimiquire, 2150 m (MHNLS 17668).

**Colubridae**

*Imantodes cenchoa.* SUCRE: Vertiente Sur de Cerro Humo, Península de Paria, 800 m (MBLUZ 1308).

*Leptophis occidentalis.* YARACUY: Sierra de Aroa, sector Palo Quemao 200 m (EBRG 3832). Yumare, vía Marín, 300 m (EBRG 4368). Parque Nacional Yurubí, Mayorica, 300 m (EBRG5048).

*Mastigodryas boddaertii*. ANZOATEGUI: Cerro El Guamal, sector occidental del macizo del Turimiquire, 2150 m (MHNLS 17669).

*Phrynonax polylepis.* SUCRE: Los Mangos, Parque Nacional Península de Paria, Hacienda Cabrera, 150 m (EBRG 3708). Carretera a Cerro Humo, Península de Paria,600 m (EBRG 2840).

*Tantilla melanocephala.* SUCRE: Cerro Campeare (MBLUZ 1425). Camino entre Macuro y Los Chorros, Península de Paria (MBLUZ 1291).

**Elapidae**

*Micrurus circinalis*. SUCRE: Hacienda La Rinconada, 5 km N de Cumanacoa, 230 m (MCNC 2199, 2447, 2478, 2964, 3061, 3242, 3402, 6436, 3644–3645). San Fernando, Distrito Montes (MCNC 3220). Rio Arenas, Distrito Montes (MCNC 2073). MONAGAS: La Margarita, Municipio Caripe, 1200 m (EBRG 6627).

*Micrurus diutius*. SUCRE: San Fernando, Distrito Montes (MCNC 2071).

*Micrurus isozonus*. SUCRE: San Fernando, Distrito Montes (MCNC 1773–1774).

**Viperidae**

*Bothrops venezuelensis*. SUCRE: Cachipal, Península de Paria, 800 m (UTA uncatalogued). Vertiente Sur de Cerro Humo, Península de Paria (EBRG 4114, MHNLS 13349).

*Lachesis muta*. MONAGAS: La Margarita, Municipio Caripe, 1200 m (EBRG 6621). SUCRE: Cipara (EBRG 6637).

**Regional endemic species**

Here we summarize the endemic species for the various regions within the coastal mountain complex

In the Serranía del Interior (the southern region of the CCR), we find the glass frog *Hyalinobatrachium orocostale*, and the stream frog *Mannophryne obliteratta* and an undescribed species of *Mannophryne* [1, 2, 3]. In the Serranía del Litoral, in the North part of CCR, the gecko *Gonatodes taeniae* and three species of stream frogs of the genus *Mannophryne* (*M. herminae*, *M. neblina,* and *M. vulcano*) as well as an undescribed species of glass frog of the genus *Hyalinobatrachium* [2].

Seveteen endemic species were found in the PR, representing 23 % of the 75 native species known for this montane system (Table 1). From West to East, the three sub-regions that belong to the PR are Cerros Campeare, La Cerbatana and Serranía de Paria (Figure 1). Most of the endemism is only known from the mountains located on the westernmost sector of Serranía de Paria, with the frog species *Allobates caribe*, *Celsiella vozmedianoi*, *Pristimantis hoogmoedi*, *P. longicorpus*, *P. pariagnomus*, and the lizards *Anadia pariaensis*, *Euspondylus monsfumus,* and *Taeniophallus nebularis*. In the easternmost sector, one species may represent the only endemic, an undescribed species of the gecko genus *Pseudogonatodes*. Similarly, an undescribed species of thin-toed frog from the genus *Leptodactylus* has only been found on La Cerbatana, in the Western sector of the Paria Range. The dwarf marsupial frog species *Flectonotus fitzgeraldi*, the stream frog *Mannophryne venezuelensis*, the rain frog *Pristimantis nubisilva,* and the Variegated Gecko *Gonatodes ceciliae*, believed to be present to the Serranía de Paria, and TRI were found during this study on Campeare and La Cerbatana. Among islands on the continental platform near the eastern Venezuelan coast, most endemics are found on TOB (9 species, 17%), closely followed by those on TRI (7 species, 10 %) and on IMA (4 species, 9%) (Table 1). Among the amphibians and reptiles of the islands of TRI-TOB, the stream frog genus *Mannophryne*, the day gecko genus *Gonatodes* and the parrot snake *Leptophis* have endemic species on each island, while the rain frogs *Pristimantis*, the microteiid *Bachia,* and the dipsasdid snake genus *Erythrolamprus* have five endemic species in Tobago, while the microteiid genus *Oreosaurus*, and the snake genera *Ninia* and *Epictia* have three endemic species in Trinidad. The skink *Copeoglossum margaritae,* the diurnal gecko *Gonatodes machelae*, the colubrid snake *Drymarchon margaritae* and the gymnophthalmid lizard *Cnemidophorus senectus* are endemic to IMA. However, *Cnemidophorus senectus* is also found on the satellite island of Cubagua.

Among the 70 species found in the TUR, 11 % are endemic (4 amphibians and 4 reptiles) (Table 1). One species, the microteiid *Anadia blakei*, was believed to be endemic to TUR, but has also been observed in Cerro Humo, in the Serranía de Paria [4].

The SSL has 45 species of amphibians and reptiles. The toad *Rhinella sclerocephala*, one undescribed species of the stream frog genus *Mannophryne*, and a tropical salamander in the genus *Bolitoglossa* seem to be endemic [5, 6, 7]. The Sierra de Aroa, considered here as the westernmost segment of the Central Coastal Range (along with the Sierra de Bobare), is the only known locality for the frogs *Dendropsophus yaracuyanus* and *Mannophryne molinai*.

**Non-native species**

According to our literature review and unpublished data, there are 12 introduced species within the mountain systems studied (Table 1). Ten were exotic with an origin in Africa, Oceania, Asia, Lesser Antilles, Middle, and North America and two species are known from other regions of Venezuela (transferred species) (Supporting Information 4).

The CCR harbor the highest number of introduced taxa (seven species). The mountains of TRI and TUR possess three introduced species each. SNSM, IMA and TOB with two species, and finally PR has one exotic species. At the moment, no records of introduced species are known from SSL.

There are seven introduced species in the CCR, the region with the most introduced species in Venezuela. Among them, three species are geckos (*Hemidactylus mabouia*, *H. frenatus* and *Lepidodactylus lugubris*), the Barbados anole (*Anolis extremus*) and the lesser Antillean frog *Eleutherodactylus johnstonei*, which are associated with urban and peri-urban environments. Two species are transferred, the spectacled caiman (*Caiman crocodilus*) and the Orinoco turtle (*Podocnemis expansa*), which inhabit this region. A population of the former species exist in the Embalse Santa Elena, an artificial reservoir at 700 m asl, while the latest have been introduced is the Lago de Valencia, a natural lagoon located between the Serrania del Litoral and Serrania del interior in CCR.

In the IMA, only two species of geckos (*Hemidactylus mabouia* and *H. frenatus*) were found as introduced, both species in urban and peri-urban environment within the Island.

Three introduced species that inhabit TRI are originally from the Lesser Antilles (*Anolis aeneus* and *A. trinitatis*) and Africa (*Hemidactylus mabouia*). Although *A. trinitatis*, was described from Trinidad, it is native to St. Vincent. The population introduced in Trinidad are known to hybridise with *A. aeneus* [8]. Although *Hemidactylus mabouia* has an African origin; it was described in 1818 from Haiti (Greater Antilles), where it was already established, thus the Trinidad population very likely originated from the Hispaniola.

Tobago has three introduced species, the Grenada tree anole (*Anolis richardii*), a Grenada bank endemic, the Grenada bush anole (*Anolis aeneus*) and the house gecko (*Hemidactylus mabouia*) [8]; their presence is likely the result of accidental human transport.

Three introduced species are known in TUR, all of them associated with urban peri-urban environments. Similarly to occur in the CCR, the lesser Antillean frog *Eleutherodactylus johnstonei* [9] and the gecko *Hemidactylus mabouia* are strictly associated with urban environments, like garden, houses or abandoned edifications, while the black iguana (*Ctenosaura similis*), from Central America has been introduced in the surrounding hills of TUR.

As for the PR, the house gecko *Hemidactylus mabouia*, has been present at least from the second half of the past century [10].

**References**

1 La Marca E. Taxonomy of the frogs of the genus *Mannophryne* (Amphibia: Anura: Dendrobatidae). Pub Asoc Amigos de Doñana 1995;4:1-75.

2 Castroviejo-Fisher S, Señaris JC, Ayarzagüena J, Vilá C. Resurrection of *Hyalinobatrachium orocostale* and notes on the *Hyalinobatrachium orientale* species complex (Anura: Centrolenidae). Herpetologica 2008; 64:472-484.

3 Manzanilla J, La Marca E, García-París M. Phylogenetic patterns of diversification in a clade of Neotropical frogs (Anura: Aromobatidae: *Mannophryne*). Biol J Linn Soc 2009;97:185-199.

4 Myers CW, Rivas G, Jadin RC. New species of lizards from Auyantepui and La Escalera in the Venezuelan Guayana, with notes on "microteiid" hemipenes (Squamata: Gymnophthalmidae). Am Mus Novit 2009;3660:1-31.

5 Linares O. Una salamandra del género *Bolitoglossa* de la cueva de Hueque, Sierra de San Luis, Venezuela. Bol Soc Venez Espeleol 1974;5:143-147.

.

6 Mijares-Urrutia A, Arends A. Herpetofauna of Estado Falcón, Northwestern Venezuela: a checklist with geographical and ecological data. Smithsonian Herpetol Infor Serv 2000 ;123:1-30.

7 Mijares-Urrutia AE, Arends A. A new toad of the *Bufo margaritifer* complex (Amphibia, Bufonidae) from northwestern Venezuela. Herpetologica 2001;57:523-531

8 Henderson RW, Powell R. Amphibians and reptiles of the St. Vincent and

Grenada banks, West Indies. Edition Chimaira. Frankfurt am Main 2018; 448 pp.

9 Kaiser H, Barrio-Amorós CL, Trujillo JD, Lynch J. Expansion of *Eleutherodactylus johnstonei* in northern South America: rapid dispersal through human interactions. Herpetol Rev 2002;33:290-294.

10 Barrio-Amorós CL, Ortíz JC. Venezuelan geckos (Gekkonidae, Phyllodactylidae, Spharodactylidae) in the collection of the Universidad de Concepción in Chile, with description of the type series of *Gonatodes ligiae* and *Gonatodes petersi* (Spharodactylidae). Zootaxa 2016;4136:537−552.
