# Supplementary material for: Biogeographical patterns of amphibians and reptiles in the northernmost coastal montane complex of South America
Source: PLoS One. 2021 Mar 4;16(3):e0246829. doi: 10.1371/journal.pone.0246829 (PMC7932178; doi:10.1371/journal.pone.0246829)
Supplement: S2 Text — (DOCX) [file pone.0246829.s008.docx]

**S6 Text**

Acosta Galvis and Sá 2018, Alemán 1952, Avila-Pires and Hoogmoed 2000, Ayarzagüena and Señaris 1996, Bailey and Thomas 2006, Barrio 1999 a-b, Barrio-Amorós and Calcaño 2003, Barrio-Amorós and Kaiser 2008, Barrio-Amorós and Ortíz 2016, Barrio-Amorós and Rivas 2003, 2008, Barrio-Amorós et al. 2010 a-b, 2019, Bisbal 1993, Blanco-Torres et al. 2013, Carvajal-Cogollo et al. 2012 Carvalho 1997, Castroviejo-Fisher et al. 2008, Cole et al. 2013, Cordero 1987, de Sá et al. 2014. Dixon 1983, Dixon and Hendricks 1979, Dixon and Huey 1970, Dixon and Kofron 1983, Dixon and Rivero-Blanco 1985, Dixon et al. 1993, Doan and Castoe 2005, Donoso-Barros “1964” [1965], 1965, 1966 a-b, 1968, Duellman 1972, Esqueda et al. 2001, Flores Padrón et al. 2016, Gans 1962, Hardy 1984, Harris 1982, 1994, Hedges and Conn 2012, Infante-Rivero et al, 2005, Kaiser et al. 1994, 2002, 2003, 2015, Kok et al. 2007, Kornacker 1999, La Marca 1992, 1993, 1994, Lancini 1962, 1963, Lemoine et al. 1998. Lötters et al. 2004, Lotzkat 2007, Lotzkat et al. 2007, 2008, Lutz 1927, Lynch and La Marca 1993, Manzanilla and La Marca 2002, Manzanilla and Sánchez 2004, Manzanilla et al. 1995, 1996, 1997,2001, 2007 a-b, 2009, Marcuzzi 1950 a-b, Markezich and Barrio-Amorós 2004, Maturana 1962, Meek 1910, Michaud and Dixon 1987, Mijares-Urrutia and Arends 1995, 1997, 1999 a-b, 2000, 2001, Mijares-Urrutia and Rivero 2000, Mijares-Urrutia et al. 1992, 2000, Miralles and Carranza 2010, Miralles et al. 2005, 2009, Murphy et al. 2018, Myers et al. 1991, 2009. Natera-Mumaw 2008. Natera-Mumaw and Manzanilla 2000, Natera-Mumaw, et al. 2006, 2015, Navarrete et al. 2005, Nogueira et al. 2019, Passos and Fernandes 2008, Rivas 1998, 2002 a-b, Rivas and Barrio-Amorós 2005, Rivas and De Freitas 2015 a-b, Rivas and Fuentes 2002, Rivas and Manzanilla 1999, Rivas and Oliveros 1997, Rivas et al. 1999, 2000, 2001 a-b, 2005 a-c, 2006, 2012, 2018, Rivero 1964, 1982, 1984, Rivero-Blanco 1979, Rivero-Blanco and Schargel 2012, 2020, Rojas-Runjaic et al. 2008, 2014, 2015 “2012”, 2018, Romero-Martínez and Lynch 2012, Roux 1927, 1929, Roze 1952 a-b, 1958, 1959 a-b, 1961, 1963, 1964 a-b, 1966, 1996, Ruthven 1922, Sánchez‐Pacheco et al. 2017, Schargel et al. 2005, Schmidt 1932, Señaris and Ayarzagüena 2005, Señaris et al. 2018, Shreve 1947, Smith et al. 2011, Solano 1969, Stejneger 1901, Test et al. 1966, Ugueto and Rivas 2010, Ugueto et al. 2009, 2013, Valera-Leal et al. 2011, Vanzolini and. Calleffo 2002, Wilson 1980, Zaher and Caramaschi 2000.

**References**

Acosta Galvis AR, de Sá RO. *Leptodactylus validus* Garman, 1888 in Colombia: its distribution and identification. ZooKeys. 2018; 737:113-23.

Alemán C. Apuntes sobre reptiles y anfibios de la región Baruta- El Hatillo. Mem Soc Cien Nat La Salle*.* 1952; 31:11-30.

Avila-Pires TCS, Hoogmoed MS. On two new species of *Pseudogonatodes* Ruthven, 1915 (Reptilia: Squamata: Gekkonidae), with remarks on the distribution of some other sphaerodactyl lizards. Zool Meded. 2000; 73:209-23.

Ayarzagüena J, Señaris JC. Dos nuevas especies de *Cochranella* (Anura; Centrolenidae) para Venezuela. Publ Asoc Am Doñana. 1996; 8:1-16.

Bailey JR, Thomas RA. A revision of the South American snake genus *Thamnodynastes* Wagler, 1830 (Serpentes: Colubridae, Tachymenini). II. Three new species from northern South America, with further descriptions of *Thamnodynastes gambotensis* Pérez-Santos and Moreno and *Thamnodynastes ramonriveroi* Manzanilla and Sánchez. Mem Fund La Salle Cien Nat. 2006; 166:7-27.

Barrio CL. Geographic distribution. *Gastrotheca ovifera.* Herpetol Rev. 1999a; 30:106.

Barrio CL. Geographic distribution. *Gastrotheca walkeri.* Herpetol Rev. 1999b; 30:106.

Barrio-Amorós CL, Calcaño D. First record of *Micrurus lemniscatus* (Linnaeus, 1758) from western Venezuela with comments on coral snakes from the eastern Andean piedmont. Herpetozoa. 2003; 16: 73-8.

Barrio-Amorós CL, Kaiser H. Distribution of *Strabomantis biporcatus* (Anura: Terrarana)

in northern Venezuela, with comments on its phenotypic variation. Salamandra. 2008;

44:248-54.

Barrio-Amorós CL, Ortíz JC. Venezuelan geckos (Gekkonidae, Phyllodactylidae, Spharodactylidae) in the collection of the Universidad de Concepción in Chile, with description of the type series of *Gonatodes ligiae* and *Gonatodes petersi* (Spharodactylidae). Zootaxa. 2016; 4136:537-52.

Barrio-Amorós CL, Rivas G. Geographic distribution. *Basiliscus basiliscus*. Herpetol Rev. 2003*;* 34: 165.

Barrio-Amorós CL, Rivas G. Spiny-tailed iguanas (*Ctenosaura similis*) in Venezuela: a preliminary report. Iguana. 2008; 15:161.

Barrio-Amorós CL, Rivas G, Molina C, Santos JC, Kaiser H. Intraspecific variation in the endangered frog *Mannophryne riveroi* (Anura, Dendrobatidae, Aromobatinae), with comments on coloration and natural history. Herpetol Not. 2010a; 3:151-60.

Barrio-Amorós CL, Rojas-Runjaic FJM, Señaris JC. Catalogue of the amphibians of Venezuela: illustrated and annotated species list, distribution, and conservatio*n.* Amph Rept Cons. 2019; 13 [special section]: 1-198.

Barrio-Amorós CL, Santos JC, Molina C. An addition to the diversity of dendrobatid frogs in Venezuela: description of three new collared frogs (Anura: Dendrobatidae: *Mannophryne*). Phyllomedusa. 2010b; 9: 3-35.

Bisbal FJ. Inventario preliminar de la fauna de la cuenca del rio Morón, estado Carabobo, Venezuela. Acta Cient Venez. 1993; 44: 365-82.

Blanco-Torres A, Báez L, Patiño-Flores E, Renjifo-R JM. Herpetofauna from the middle valley of the Ranchería river, La Guajira, Colombia. Rev. Biodivers. Neotrop. 2013; 3: 113-22

Carvajal-Cogollo JE, Cárdenas-Arévalo G, Castaño-Mora O. Reptiles de la región Caribe de Colombia. In: Rangel JO, editor. Colombia Diversidad Biótica XII. La región Caribe de Colombia. Bogotá: Universidad Nacional de Colombia; 2012. p. 791-812.

Carvalho CM. Uma nova espécie de microteiideo do gênero *Gymnophthalmus* do estado de Roraima, Brasil (Sauria, Gymnophthalmidae). Pap Avul Zool. 1997; 40: 161-74.

Castroviejo-Fisher S, Señaris JC, Ayarzagüena J, Vilá C. Resurrection of *Hyalinobatrachium orocostale* and notes on the *Hyalinobatrachium orientale* species complex (Anura: Centrolenidae). Herpetologica 2008; 64: 472-84.

Cole CJ, Townsend CR, Reynolds RP, MacCulloch RD, Lathrop A. Amphibians and reptiles of Guyana, South America: illustrated keys, annotated species accounts, and a biogeographic synopsis. Proc. Biol. Soc. Wash. 2013; 125: 317-578

Cordero GA. Composición y diversidad de la fauna de vertebrados terrestres de Barlovento, Estado Miranda, Venezuela. Acta Cient Venez. 1987; 38:234-58.

de Sá RO, Grant T, Camargo A, Heyer WR, Ponssa ML, Stanley E. Systematics of the Neotropical genus *Leptodactylus* Fitzinger, 1826 (Anura: Leptodactylidae): phylogeny, the relevance of nonmolecular evidence, and species accounts. S Amer J Herpetol. 2014; 9 Suppl 1: S1-S128.

Dixon JR. Systematics of *Liophis reginae* and *L. williamsi* (Serpentes, Colubridae), with a description of a new species. Ann Carn Mus. 1983; 52: 113-38.

Dixon JR, Hendricks FS. The wormsnakes (Family Typhlopidae) of the Neotropics, exclusive of the Antilles. Zool Verhand. 1979; 173: 1-39.

Dixon JR, Huey RB. Systematics of the lizards of the gekkonid genus *Phyllodactylus* of mainland South America. Los Angeles Co Mus Contr Sci. 1970; 192: 1-78.

Dixon JR, Kofron CP. The Central and South American anomalepid snakes of the genus *Liotyphlops*. Amphibia-Reptilia. 1983; 4: 241-64.

Dixon JR, Rivero-Blanco C. A new dendrobatid frog (*Colostethus*) from Venezuela, with notes on its natural history and that of related species. J Herpetol*.* 1985; 19:177-84.

Dixon JR, Wiest JA, Cei JM. Revision of the Neotropical snake genus *Chironius* Fitzinger (Serpentes, Colubridae). Torino: Mus reg Sci nat; 1993.

Doan TM, Castoe TA. Phylogenetic taxonomy of the Cercosaurini (Squamata: Gymnophthalmidae), with new genera for species of *Neusticurus* and *Proctoporus*. Zool J Linn Soc. 2005; 143:405-16.

Donoso-Barros R. A new Dendrobatidae [sic] frog, *Prostherapis riveroi* from Venezuela. Carib. J Sci. “1964” 1965; 4:485-89.

Donoso-Barros R. Nuevos reptiles y anfibios de Venezuela. Not Mens Mus Nac Hist Nat. 1965; 102:2-3.

Donoso-Barros R. *Hyla robersimoni*, nuevo hylidae de Venezuela. Bol Mus Nac Hist Nat. 1966a; 29:37-43.

Donoso-Barros R. Dos nuevos *Gonatodes* de Venezuela. Publ Ocas Mus Nac Hist Nat. 1966b; 11:1-32.

Donoso-Barros R. The lizards of Venezuela (checklist and key). Carib J Sci. 1968; 8:105-22.

Duellman WE.South American frogs of the *Hyla rostrata* group (Amphibia, Anura, Hylidae). Zool Meded. 1972*;* 47:177-92+3 pl.

Esqueda LF, La Marca E, Natera M, Battiston P. Noteworthy reptilian state records and a lizard species new to the herpetofauna of Venezuela. Herpetol Rev. 2001; 32: 198-200.

Flores Padrón D, De Freitas M, Camargo E. Primer reporte al norte del río Orinoco para *Siphlophis cervinus* (Laurenti, 1768) (Serpentes: Dipsadidae: Xenodontinae) en la Península de Paria, estado Sucre, Venezuela. Saber (Universidad de Oriente). 2016; 28: 171-76.

Gans C. Notes on amphisbaenids (Amphisbaenia, Reptilia). 5. A redefinition and bibliography of *Amphisbaena alba* Linné. Amer Mus Nov. 1962; 2105:1-31.

Hardy JD. Systematic status of the South American frog “*Phyllobates mandelorum”*

(Amphibia, Dendrobatidae). Bull Maryland Herpetol Soc.1984; 20:109-11.

Harris DM. The *Sphaerodactylus* (Sauria: Gekkonidae) of South America. Occ Pap Mus Zool Univ Michigan. 1982; 704:1-31.

Harris DM. Review of the teiid lizard genus *Ptychoglossus*. Herpetol Monog. 1994; 8: 226-75.

Hedges SB, Conn CE. A new skink fauna from Caribbean islands (Squamata, Mabuyidae, Mabuyinae*).* Zootaxa. 2012; 3288: 1-244.

Infante-Rivero EE, Veloso P. Rojas-Runjaic FJM. Geographic Distribution: *Drymarchon caudomaculatus*. Herpetol Rev 2005; 36: 20.

Kaiser H, Barrio-Amorós CL, Rivas G, Grismer LL. Geographic Distribution. *Siphlophis compressus.* Herpetol Rev. 2003; 34:170.

Kaiser H, Barrio-Amorós CL, Rivas G, Steinlein C, Schmid M. Five new species of *Pristimantis* (Anura: Strabomantidae) from the coastal cloud forest of the Península de Paria, Venezuela. J Threat Taxa. 2015; 7:7047-88.

Kaiser H, Barrio-Amorós CL, Trujillo JD, Lynch J. Expansion of *Eleutherodactylus johnstonei* in northern South America: rapid dispersal through human interactions. Herpetol Rev. 2002; 33: 290-94.

Kaiser H, Hardy JD, Green DM. Taxonomic status of Caribbean and South American frogs currently ascribed to *Eleutherodactylus urichi* (Anura: Leptodactylidae). Copeia. 1994; 780-96.

Kok PJR, Rivas Fuenmayor GA, Pauwels OSG. The taxonomic status of the Venezuelan snakes *Atractus matthewi* and *A. nororientalis* (Serpentes, Colubridae). Zootaxa. 2007; 1493:66-8.

Kornacker PM. Checklist and Key to the Snakes of Venezuela/Lista Sistemática y Claves para las Serpientes de Venezuela. Rheinbach: PaKo-Verlag; 1999.

La Marca E. Catálogo taxonómico, biogeográfico y bibliográfico de las ranas de Venezuela. Mérida: Universidad de Los Andes; 1992.

La Marca, E. Phylogenetic relationships and taxonomy of *Colostethus mandelorum* (Anura: Dendrobatidae), with notes on coloration, natural history, and description of the tadpole. Bull Maryland Herpetol Soc. 1993; 29:4-19.

La Marca E. Taxonomy of the frogs of the genus *Mannophryne* (Amphibia: Anura: Dendrobatidae). Publ Asoc Am Doñana. 1994; 4:1-75.

Lancini AR. Contribución al conocimiento de los ofidios del Cordón Litoral. Los ofidios de Curupao, estado Miranda (Venezuela). Acta Biol Venez. 1962; 3: 161-72.

Lancini AR. Herpetofauna de la isla de Patos (Golfo de Paria, Venezuela). Bol Soc Ven Cien Nat. 1963; 103:247-54.

Lancini AR. Una nueva especie del genero *Anadia* (Sauria: Teiidae) de Venezuela. Publ Ocas Mus Cien Nat. 1963; 4:1-2.

Lemoine K, Rivas G, Manzanilla J. Geographic distribution. *Pseustes poecilonotus polylepis*. Herpetol Rev. 1998; 29:115.

Lötters S, La Marca E, Vences M. Redescriptions of two toad species of the genus *Atelopus*

from Coastal Venezuela. Copeia. 2004; 222-34.

Lotzkat S. [Taxonomy and zoogeography of the herpetofauna of the Nirgua massif, Venezuela] [dissertation]. Frankfurt am Main: Johann Wolfgang Goethe-Universität; 2007.

Lotzkat S, Hertz A, Valera-Leal J. Amphibia, Anura, Hylidae, *Hylomantis medinai*:

distribution extension by discovery of a third population. Check List. 2007; 3:200-03.

Lotzkat S, Natera-Mumaw M, Hertz A, Sunyer J, Mora D. New state records of *Dipsas variegata* (Duméril, Bibron and Duméril 1854) (Serpentes: Colubridae) from northern Venezuela, with comments on natural history. Herpetotropicos. 2008; 4: 25-29.

Lutz A. Notas sobre batrachios da Venezuela e da ihla de Trinidad. Mem Inst Oswaldo Cruz. 1927; 20: 35-65.

Lynch JD, La Marca E. Synonymy and variation in *Eleutherodactylus bicumulus* (Peters) from Northern Venezuela, with a description of a new species (Amphibia: Leptodactylidae). Carib J Sci. 1993; 29:133-46.

Manzanilla J, Fernández-Badillo A, La Marca E, Visbal R Fauna del Parque Nacional Henri Pittier, Venezuela: composición y distribución de los anfibios. Acta Cient Venez. 1995; 46: 294-302.

Manzanilla J, Fernández-Badillo A, Visbal R. Fauna del parque nacional Henri Pittier, Venezuela: composición y distribución de los reptiles. Acta Cient Venez. 1996; 47: 191-204.

Manzanilla J, Jowers MJ, La Marca E, García-París M. Taxonomic reassessment of *Mannophryne trinitatis* (Anura: Dendrobatidae) with a description of a new species from Venezuela. Herpetol J. 2007a; 17: 31-42.

Manzanilla J, La Marca E. Museum records and field samplings as source of data indicating population crashes for *Atelopus cruciger*, proposed critically endangered species from the Venezuelan coastal range. Mem Fund La Salle Cien Nat 2002; 157: 5-30.

Manzanilla J, La Marca E, Esqueda LF. Geographic distribution. *Dipsas latifrontalis*. Herpetol Rev. 2001; 32: 195.

Manzanilla J, La Marca E, García-París M. Phylogenetic patterns of diversification in a clade of Neotropical frogs (Anura: Aromobatidae: *Mannophryne*). Biol J Linn Soc. 2009; 97: 185-99.

Manzanilla J, La Marca E, Jowers M, Sánchez D, García-París M. Un nuevo *Mannophryne* (Amphibia: Anura: Dendrobatidae) del macizo del Turimiquire, noreste de Venezuela. Herpetotropicos. 2007b; 2: 105-13.

Manzanilla J, Rivero R, Natera M. Geographic distribution. *Kentropyx striata*. Herpetol Rev.1997; 28: 50.

Manzanilla J, Sánchez D. Una nueva especie de *Thamnodynastes* (Serpentes: Colubridae) del macizo del Turimiquire, noreste de Venezuela. Mem Fund La Salle Cien Nat. 2004; 161-162: 61-75.

Marcuzzi G. Breves apuntes sobre algunos lagartos de Venezuela septentrional. Mem. Soc Cien Nat La Salle. 1950a; 26:73-110.

Marcuzzi G. Ofidios existentes en las colecciones de los museos de Caracas (Venezuela). Nov Cient, contrib ocas, Mus Hist Nat La Salle, sér zool*.* 1950b; 3:1-20.

Markezich A, Barrio-Amorós CL. A new species of *Atractus* (Serpentes: Colubridae) from Northeastern Venezuela. Bull Maryland Herpetol Soc. 2004; 40:111-21.

Maturana HR. A study of the species of the genus *Basiliscus*. Bull Mus Comp Zool. 1962; 128:1-33.

Meek SE. Notes on batrachians and reptiles from the islands north of Venezuela. Field Mus Nat Hist zool. ser. 1910; 7: 415-418.

Michaud EJ, Dixon JR. Taxonomic revision of the *Liophis lineatus* complex (Reptilia: Colubridae) of Central and South America. Contrib Biol Geol, Milw Pub Mus*.* 1987; 71:1-26.

Mijares-Urrutia A, Arends A. Aportes al conocimiento de *Gonatodes falconensis* Shreve 1947 (Lacertilia: Gekkonidae) del noroeste de Venezuela. Amphibia-Reptilia 1995; 16:203-210.

Mijares-Urrutia A, Arends A. A new *Mabuya* (Squamata: Scincidae) from the semiarid coast of northwestern Venezuela. Rev. Brasil Biol. 1997; 57: 595-601.

Mijares-Urrutia A, Arends A. Additional new regional and local records of amphibians and reptiles from the state of Falcón, Venezuela. Herpetol Rev. 1999a; 30: 115.

Mijares-Urrutia A, Arends A. El género *Anolis* (Squamata: Polychrotidae) en el estado Falcón, noroeste de Venezuela: lista de especies, variación y distribución. Com Mus Cien Tec PUCRS, ser zool. 1999b; 12:45-70.

Mijares-Urrutia A, Arends A. Herpetofauna of Estado Falcón, northwestern Venezuela: a checklist with geographical and ecological data. Smith Herpetol Inf Serv 2000 ; 123:1-30.

Mijares-Urrutia A, Arends A. A new toad of the *Bufo margaritifer* complex (Amphibia, Bufonidae) from northwestern Venezuela. Herpetologica. 2001; 57:523-31.

Mijares-Urrutia A, Rivero R. A new treefrog from the Sierra de Aroa, northern Venezuela. J Herpetol. 2000; 34:80-4.

Mijares-Urrutia A, Señaris JC, Arends A. *Anolis tigrinus* Peters 1863 (Lacertilia: Polychridae): redescripción y distribución en Venezuela. Mem. Soc. Cien Nat La Salle 1992; 138:123-32.

Mijares-Urrutia A, Señaris JC, Arends A. Taxonomía de algunos microtéidos (Squamata) de Venezuela, I: variación y distribución geográfica de *Euspondylus acutirostris* y descripción de un nuevo *Euspondylus* del nordeste de Venezuela. Rev Biol Trop. 2000; 48: 671-80.

Miralles A, Carranza S. Systematics and biogeography of the Neotropical genus *Mabuya*, with special emphasis on the Amazonian skink *Mabuya nigropunctata* (Reptilia, Scincidae). Mol Phyl Evol. 2010; 54: 857-69.

Miralles A, Rivas G, Barrio-Amorós CL. Taxonomy of the genus *Mabuya* (Reptilia, Squamata, Scincidae) in Venezuela. Zoosystema. 2005; 27: 825-37.

Miralles A, Rivas G, Bonillo C, Schargel WE, Barros TR, García-Pérez JE, Barrio-Amorós CL. Molecular systematics of Caribbean skinks of the genus *Mabuya* (Reptilia, Scincidae), with descriptions of two new species from Venezuela. Zool J Linn Soc. 2009; 156: 598-616.

Murphy JC, Downie JR, Smith JM, Livingstone SM, Mohammed RS, Lehtinen RM, Eyre M, Sewlal Jo-AN, Noriega N, Casper GS, Anton T, Rutherford MG, Braswell AL, Jowers MJ. A field guide to the amphibians and reptiles of Trinidad & Tobago. Port of Spain: Trinidad & Tobago Field Naturalists´Club; 2018.

Myers CW, Paolillo A, Daly JW. Discovery of a defensively malodorous and nocturnal frog in the family Dendrobatidae: phylogenetic significance of a new genus and species from the Venezuelan Andes. Amer Mus Nov*.* 1991; 3002:1-33.

Myers CW, Rivas G, Jadin RC. New species of lizards from Auyantepui and La Escalera in the Venezuelan Guayana, with notes on "microteiid" hemipenes (Squamata: Gymnophthalmidae). Amer Mus Nov. 2009; 3660:1-31.

Natera-Mumaw M. Nuevos registros geográficos y notas bioecológicas de *Dendrophidion dendrophis* (Schlegel, 1837) y *Dendrophidion nuchale* (Peters, 1863) (Serpentes: Colubridae) en Venezuela, con comentarios sobre la taxonomía de *Dendrophidon nuchale*. Herpetotropicos. 2008; 4: 11-6.

Natera-Mumaw M, Acosta JC, Battiston P, Hidalgo O. Distribution extension and new state record for *Micrurus lemniscatus diutius* Burger, 1955 (Reptilia: Elapidae) in Venezuela. Herpetotropicos. 2006; 3: 59.

Natera-Mumaw M, Esqueda LF, Castelaín M. Atlas Serpientes de Venezuela: una visión actual de su diversidad. Santiago: privately printed; 2015.

Natera-Mumaw M, Manzanilla J. Nuevos registros geográficos y notas bioecológicas de *Philodryas olfersii* (Lichtenstein, 1823) (Serpentes: Colubridae) en Venezuela. Mem Fund La Salle Cien Nat*.* 2000; 153: 51-9.

Navarrete LF, Rodríguez-Acosta A, Contreras Y, Briceño JM. *Stenorrhina degenhardtii ocellata* Jan, 1976 in Venezuela. Herpetozoa. 2005; 18:89-91.

Nogueira CC, Argôlo AJS, Arzamendia V, Azevedo JA, Bardo FE, Bérnils RS, Bolochio BE, Borges-Martins M, Brasil-Godinho M, Braz H, Buononato MA, Cisneros-Heredia DF, Colli GR, Costa HC, Franco FL, Giraudo A, González RC, Guedes T, Hoogmoed MS, Marques OAV, Montingelli GG, Passos P, Prudente ALC, Rivas GA, Sánchez PM, Serrano FC, Silva Jr. NJ, Strussmann C, Vieira-Alencar JPS, Zaher H, Sawaya RJ, Martins M. Atlas of Brazilian snakes: verified point-locality maps to mitigate the Wallacean Shortfall in a megadiverse snake fauna*.* S Amer J Herpetol. 2019; 14 Suppl 1:S1-S274.

Oftedal OT. A revision of the genus *Anadia* (Sauria, Teiidae). Arq Zool S Paulo. 1974; 25:203-265.

Passos P, Fernandes R. Revision of the *Epicrates cenchria* complex (Serpentes: Boidae). Herpetol. Monog. 2008; 22: 1-30.

Rivas G. Geographic distribution. *Oxyrhopus doliatus*. Herpetol. Rev.2002a; 33: 150.

Rivas G. Geographic distribution. *Hemidactylus palaichthus*. Herpetol Rev. 2002b; 33: 224-5.

Rivas G. Geographic distribution. *Atelopus cruciger*. Herpetol. Rev. 1998; 29: 172.

Rivas G, Barrio-Amorós CL. New amphibian and reptile records from Cojedes state, Venezuela. Herpetol. Rev*.*2005; 36:205-9.

Rivas G, De Freitas M. Discovery of the critically endangered Golden Tree Frog, *Phytotriades* *auratus* (Boulenger, 1917) in eastern Venezuela, with comments on its distribution, conservation, and biogeography. Herpetol Rev. 2015a; 46: 153-7.

Rivas G, De Freitas M. Geographic distribution. *Anolis tigrinus*. Herpetol Rev. 2015b; 46: 216.

Rivas G, De Freitas M, Kaiser H, Barrio-Amorós CL, Barros TR. Amphibians of the Península de Paria: a pocket field guide. Frankfurt am Main: Edition Chimaira; 2018.

Rivas G, Fuentes O. Geographic distribution. *Liophis epinephelus opisthotaenius*. Herpetol Rev. 2002; 33:68.

Rivas G, La Marca E, Oliveros O. Una nueva especie de *Anadia* (Reptilia: Sauria: Gymnophthalmidae) del noreste de Venezuela. Acta Biol Venez. 1999; 19:27-32

Rivas G, Manzanilla J. Distribución geográfica de *Dactyloa squamulata* (Peters, 1863) (Reptilia: Sauria: Polychrotidae) en Venezuela. Mem Fund La Salle Cien Nat. 1999; 152:19-24.

Rivas G, Manzanilla J, Rivero R. Notas taxonómicas y distribución geográfica del género *Rhadinaea*, Cope, 1863 (Serpentes: Colubridae) en Venezuela. Mem Fund La Salle Cien Nat. 2000; 153:135-141.

Rivas G, Molina CR, Fuentes O. Geographic Distribution. *Typhlops reticulatus*. Herpetol. Rev. 2001; 32:126-127.

Rivas G, Molina CR, Ugueto GN, Barros TR, Barrio-Amorós CL, Kok PJR. Reptiles of Venezuela: an updated and commented checklist. Zootaxa. 2012; 3211:1-64.

Rivas G, Oliveros O. Herpetofauna del estado Sucre, Venezuela: lista preliminar de reptiles. Mem Soc Cien Nat La Salle. 1997; 147:67-80.

Rivas G, Rojas-Runjaic FJM, Barrio-Amorós CL. Geographic Distribution. *Pseudogonatodes manesi*. Herpetol. Rev. 2006; 37:107.

Rivas G, Señaris JC, La Marca E. Erstnachweis von *Hemidactylus mabouia* (Sauria: Gekkonidae) für die Insel Margarita (Estado Nueva Esparta), Venezuela, nebst Angaben zur geographischen Verbreitung. Herpetofauna. 2001; 23:5-8.

Rivas G, Schargel WE, Meik JM. A new species of *Riama* (Squamata: Gymnophthalmidae), endemic to the Península de Paria, Venezuela. Herpetologica. 2005a; 61:461-468.

Rivas G, Ugueto GN, Bauer AM, Barros TR, Manzanilla J. Expansion and natural history of a successful colonizing gecko in Venezuela (Reptilia: Gekkonidae: *Hemidactylus mabouia*) and the discovery of *H. frenatus* in Venezuela. Herpetol. Rev. 2005b; 36: 121-125.

Rivas G, Ugueto GN, Rivero R, Miralles A. The herpetofauna of Isla de Margarita, Venezuela: new records and comments. Carib J Sci. 2005c; 41: 346-351.

Rivero JA. The distribution of Venezuelan frogs. IV. The Coastal Range. Carib J Sci. 1964; 4: 307-319.

Rivero JA. Sobre el *Colostethus mandelorum* (Schmidt) y el *Colostethus inflexus* Rivero (Amphibia, Dendrobatidae). Mem Soc Cien Nat La Salle. 1982; 118: 9-16.

Rivero JA. Una nueva especie de *Colostethus* (Amphibia, Dendrobatidae) de la Cordillera de la Costa, con anotaciones sobre otros *Colostethus* de Venezuela. Brenesia.1984; 22: 51-6.

Rivero-Blanco C. The Neotropical lizard genus *Gonatodes* Fitzinger (Sauria: Sphaerodactylinae). [dissertation]. College Station: Texas A&M University; 1979.

Rivero-Blanco C, Schargel WE. A strikingly polychromatic new species of *Gonatodes*

(Squamata: Sphaerodactylidae) from northern Venezuela. Zootaxa. 2012; 3518:66-78.

Rivero-Blanco C, Schargel WE. A new diurnal gecko in the genus *Gonatodes* (Squamata: Sphaerodactylidae) from Margarita Island, Venezuela. Zootaxa. 2020; 4729:429-39.

Rojas-Runjaic FJM, Barrio Amorós CL, Molina C, Señaris JC, Fedon IC. Amphibia, Anura, Hylidae, *Scarthyla vigilans*: range extensions and new state records from Delta Amacuro and Miranda states, Venezuela. Check List. 2008; 4: 301-3.

Rojas-Runjaic FJM, Camargo E, Bolaños W, Mora D, Aular L, García F. A new locality and range extension for the Rancho Grande Leaf Frog *Agalychnis medinae* (Funkhouser, 1962) (Anura: Hylidae) in northern Venezuela. Check List. 2014; 10:392-4.

Rojas-Runjaic FJM, Lasso-Alcala OM, Camargo E. Actualización del conocimiento sobre la distribución geográfica del galápago pecho quebrado *Kinosternon scorpioides scorpioides* (Testudines, Kinosternidae) en Venezuela. Mem Fund La Salle Cien Nat. 2015 “2012”; 177-178: 125-133.

Rojas-Runjaic FJM, Matta-Pereira ME, La Marca E. Unveiling species diversity in collared frogs through morphological and bioacoustic evidence: a new *Mannophryne* (Amphibia, Aromobatidae) from Sierra de Aroa, northwestern Venezuela, and an amended definition and call description of *M. herminae* (Boettger, 1893). Zootaxa. 2018; 4461:451-76.

Romero-Martínez HJ, Lynch JD. Anfibios de la región Caribe. In: Rangel JO, editor. Colombia Diversidad Biótica XII. La región Caribe de Colombia. Bogotá: Universidad Nacional de Colombia; 2012. p. 677-701.

Roux J. Contribution á l’erpétologie du Vénézuéla. Verhan Natur Gesell Basel. 1927; 38:252-261.

Roux J. Sur deux reptiles nouveaux du Vénézuéla. Verhan Natur Gesell Basel. 1929; 49:29-34.

Roze JA. Contribución al conocimiento de los ofidios de las familias Typhlopidae y Leptotyphlopidae en Venezuela. Mem Soc Cien Nat La Salle. 1952a; 32:143-58.

Roze JA. Colección de reptiles del profesor Scorza, de Venezuela. Acta Biol Venez*.* 1952b; 1:93-114.

Roze JA. A new species of the genus *Urotheca* (Serpentes: Colubridae) from Venezuela. Breviora. 1958; 88:1-5.

Roze JA. Taxonomic notes on a collection of Venezuelan reptiles in the American Museum of Natural History. Amer Mus Nov. 1959a; 1934:1-14.

Roze JA. Una nueva especie del genero *Drymarchon* (Serpentes: Colubridae) de la isla de Margarita, Venezuela. Nov Cient, contrib ocas, Mus Hist Nat La Salle, sér zool. 1959b; 25:1-4.

Roze JA. El género *Atractus* (Serpentes: Colubridae) en Venezuela. Acta Biol Venez. 1961; 3:103-19.

Roze JA. Una nueva especie del género *Gonatodes* (Sauria: Gekkonidae) de Venezuela. Publ Ocas Mus Cien Nat 1963; 5:1-4.

Roze JA. The snakes of the *Leimadophis*-*Urotheca*-*Liophis* complex from Parque Nacional Henri Pittier (Rancho Grande), Venezuela, with a description of a new genus and species (Reptilia, Colubridae). Senck Biol. 1964a; 45:533-42.

Roze JA. La herpetología de la Isla de Margarita, Venezuela. Mem Soc Cien Nat La Salle. 1964b; 69: 209-41.

Roze JA. La Taxonomía y Zoogeografía de los ofidios de Venezuela. Caracas: Universidad Central de Venezuela; 1966.

Roze JA. Coral Snakes of the Americas-Biology, Identification, and Venoms. Malabar: Krieger Publishing; 1996.

Ruthven AG. The amphibians and reptiles of the Sierra Nevada de Santa Marta, Colombia.

Univ Michigan Mus Zool misc. publ. 1922; 8: 1-70+xii plates.

Sánchez‐Pacheco SJ, Torres‐Carvajal O, Aguirre‐Peñafiel V, Nunes PMS, Verrastro L, Rivas GA, Rodrigues MT, Grant T, Murphy RW. Phylogeny of *Riama* (Squamata: Gymnophthalmidae), impact of phenotypic evidence on molecular datasets, and the origin of the Sierra Nevada de Santa Marta endemic fauna. Cladistics. 2017; 34: 260-91.

Schargel WE, Rivas G, Myers CW. An enigmatic new snake from cloud forest of the Península de Paria, Venezuela (Colubridae: genus *Taeniophallus*?). Amer Mus Nov. 2005; 3484:1-22.

Schmidt KP. Reptiles and amphibians of the Mandel Venezuelan expedition. Field Mus Nat Hist. zool. ser. 1932; 37:169-63.

Señaris JC, Ayarzagüena J. Revisión Taxonómica de la familia Centrolenidae (Amphibia; Anura) de Venezuela. Sevilla: IberoMaB UNESCO; 2005.

Señaris JC, Aristeguieta Padrón MM, Rojas Gil H, Rojas-Runjaic FJM. Guía ilustrada de los anfibios y reptiles del valle de Caracas, Venezuela. Altos de Pipe: IVIC; 2018.

Shreve B. On Venezuelan reptiles and amphibians collected by Dr. H. G. Kluger. Bull Mus Comp Zool. 1947; 99: 519-37.

Smith JM, Downie JR, Dye RF, Ogilvy V, Thornham DG, Rutherford MG, Charles SP, Murphy JC. Amphibia, Anura, Hylidae, *Scarthyla vigilans* (Solano, 1971): range extension and new country record for Trinidad, West Indies, with notes on tadpoles, habitat, behavior and biogeographical significance. Check List. 2011; 7:574-7.

Solano HG. Beiträge zur kenntnis der amphibienfauna Venezuelas. Veröff Zool staat München.1969; 13:1-26.

Stejneger L. An annotated list of batrachians and reptiles collected in the vicinity of La Guaira, Venezuela, with descriptions of two species of snakes. Proc US Nat Mus. 1901; 24:179-92.

Test F, Sexton OJ, Heatwole H. Reptiles of Rancho Grande and vicinity, Estado Aragua, Venezuela. Misc Publ Mus Zool Univ Michigan 1966; 128:1-63.

Ugueto G, Rivas G. Amphibians and reptiles of Margarita, Coche and Cubagua. Frankfurt am Main: Edition Chimaira; 2010.

Ugueto G, Rivas G, Barros T, Smith EN. A Revision of the Venezuelan anoles II: redescription of *Anolis squamulatus* Peters 1863 and *Anolis tigrinus* Peters 1863 (Reptilia: Polychrotidae). Carib J Sci. 2009; 45:30-51.

Ugueto GN, Velozo P, Sánchez LE, Bermúdez-Villapol LA, Lasso-Alcalá O, Barros TR, Rivas G. Noteworthy new records of Squamate reptiles (Reptilia: Squamata) from various Venezuelan Caribbean islands, including a new addition to the herpetofauna of Venezuela. CheckList 2013; 9:1075-80.

Valera-Leal J, Acevedo A, Pérez-Sánchez A, Vega J, Manzanilla J. Registro histórico de *Gastrotheca* *ovifera* (Anura: Hemiphractidae): evidencias de disminución en selvas nubladas de la Cordillera de la Costa venezolana. Rev Biol Trop. 2011*;* 59:329-45.

Vanzolini PE, Calleffo MEV. A taxonomic bibliography of the South American snakes of the *Crotalus durissus* complex (Serpentes, Viperidae). An Acad Brasil Cien. 2002; 74:37-83.

Wilson LD. Systematics of the *melanocephala* group of the colubrid snake genus *Tantilla*. San Diego Soc Nat Hist Mem*.* 1980; 11: 1-57.

Zaher H, Caramaschi U. Synonymization of *Oxyrhopus venezuelanus* Shreve, 1947, with *Oxyrhopus doliatus* Duméril, Bibron & Duméril, 1854, and revalidation of *Oxyrhopus erdisii* (Barbour, 1913) (Serpentes, Colubridae). Dumerilia. 2000; 4:113-22.
