# Supplementary material for: Biogeographical patterns of amphibians and reptiles in the northernmost coastal montane complex of South America
Source: PLoS One. 2021 Mar 4;16(3):e0246829. doi: 10.1371/journal.pone.0246829 (PMC7932178; doi:10.1371/journal.pone.0246829)
Supplement: S3 Text — (DOCX) [file pone.0246829.s009.docx]

**S9 Text**

Undescribed amphibians and reptiles from the mountain chain of northern Venezuela, Trinidad and Tobago and Sierra Nevada de Santa Marta

*Mannophryne* sp 1. Guatopo National Park, Miranda and Guarico states (Central Coastal Range). Specimens of this population were used within the type series of *Colostethus guatopoensis* [1], a synonym of *G. obliteratta*. This species is sympatric with *M. obliteratta* on Guatopo national Park [2].

*Mannophryne* sp. 2. A, undescribed species from Bejuma, Carabobo state, Central Coastal Range [2].

*Colostethus* sp. Similar to *C. ruthveni* from Sierra Nevada de Santa Marta, Colombia [3].

*Hyalinobatrchium* sp. Undescribed species from the Serrania de Litoral, Central Coastal Range [4].

*Adenomera* sp. Previously known from the island of Trinidad as *Adenomera hylaedactyla* (Murphy et al. 2018). [5].

*Leptodactylus* sp. An undescribed species related to *L. fuscus*/*L. mystaceus* from La Cerbatana and mentioned by Rivas et al. [6] and sequenced by Rafael De Sá.

*Gastrotheca* sp. A call characteristic of the genus *Gastrotheca* has been recorded at least in four occasions, from at least four localities of the Paria Peninsula above 800 m, and most probably it belongs to a new endemic species of that area. It has not been seen or captured [6].

*Pristimantis* sp. 1. It corresponds with one or two undescribed species and previously mentioned in the literature as *Eleutherodactylus bicumulus*, *E. gollmeri* [7], *E. urichi* [8]. *Eleutherodactylus* sp. 1 and *Eleutherodactylus* sp. 2 [9]. from Turimiquire Massif. *Pristimatis urichi* is endemic to Trinidad and Tobago [5, 9].

*Dendropsophus* aff *minutus* (Sierra San Luis and Central Coastal Range). This is a species complex with broad South American distribution. Recent molecular work suggests at least four species present in Venezuela [10].

*Bolitoglossa* sp. Referred by Linares [11] from Cataratas de Hueque, Falcón state. This species is also found on Curimagua (vouchered at EBRG), both localities ubiquitous in Sierra de San Luis (currently in study by GAR and Walter Schargel).

*Anolis* sp. Trinidad and Tobago. Murphy et al. [5]. mentions it as *A. tigrinus.* Sequenced and considered an undescribed species by M. J. Jowers.

*Gonatodes* sp. (Paria Range, Turimiquire Massif), an undescribed species, believed to be conspecific with *Gonatodes machelae* from Isla de Margarita [12].

*Pseudogonatodes* sp. Paria Range [13]. This population shows genetic and morphological differences to *P. manessi*, and it is here therefore considered a new species (Rivas, Schargel and Jowers, in preparation).

*Gymnophthalmus* sp. (Central Coastal Range, Isla de Margarita, Turimiquire Massif, Paria Range, Trinidad). Undescribed species. Long confused with *G. speciosus* (type locality Honduras). This was already noted by Carvalho [14]. for the population in Central Coastal Range. Similarly, other northern South American populations with read tails do not correspond with *G. speciosus* *sensu stricto*, included in Isla de Margarita, Turimiquire Massif, Paria Range, Trinidad and Sierra Nevada de Santa Marta.

*Oreosaurus* sp. Undescribed species endemic to Turimiquire Massif. Reported as *Oreosauru*s “Venezuela” by Sánchez-Pacheco et al. [15]. based on morphology and genetic data. Currently under description by GAR.

*Tupinanbis teguixin*. We prefer to keep the use of *Tupinambis teguixin* to the population that inhabit in northern Colombia until a revision that involve enough samples from the region of the northern of South America will done. Unfortunately, Murphy et al. [16] in a first attempt to clarify the species limit of the species formerly under the name *teguixin*, did not examine specimens from much of northern South America, leaving out those of the mountains of the Sierra Nevada de Santa Marta.

*Copeoglossum* sp. This species belongs to a species from the Venezuelan northern Orinoco (Central Coastal Range and Sierra de San Luis) formerly considered as *C. nigropucntatus* or *Mabuya nigropunctata* (unnpublished data). The populations from northeastern Venezuela, at least those from the Paria Range are in fact *Copeoglosum aurae* and are likely to inhabit the lower ad medium elevations from the Turimiquire Massif.

*“Mabuya”* sp. Sierra Nevada de Santa Marta [17]. According to Andrés C. Montes-Correa (com. pers.) the skink from this locality could belong to the genus *Maracaiba*, a genus restricted to the Colombian and Venezuelan Andes, including the Serranía de Perijá, as well as Lago de Maracaibo basin.

**References**

1 Dixon JR, Rivero-Blanco C. A new dendrobatid frog (*Colostethus*) from Venezuela, with notes on its natural history and that of related species. J Herpet. 1985; 19:177-184.

2 Barrio-Amorós CL, Rojas-Runjaic FJM, Señaris JC. Catalogue of the amphibians of Venezuela: illustrated and annotated species list, distribution, and conservatio*n.* Amph Rept Cons. 2019; 13 [special section]: 1-198.

3 Grant T, Rada M, Anganoy-Criollo M, Batista A, Henrique Dias P, Moriguchi Jeckel A, Jacob Machado D, Rueda-Almonacid JV. Phylogenetic systematics of dart-poison frogs and their relatives revisited (Anura: Dendrobatoidea). S Amer J Herpetol. 2017; 12(s1):S1-S90.

4 Castroviejo-Fisher S, Señaris JC, Ayarzagüena J, Vilá C. Resurrection of *Hyalinobatrachium orocostale* and notes on the *Hyalinobatrachium orientale* species complex (Anura: Centrolenidae). Herpetologica 2008; 64: 472-84.

5 Murphy JC, Downie JR, Smith JM, Livingstone SM, Mohammed RS, Lehtinen RM, Eyre M, Sewlal Jo-AN, Noriega N, Casper GS, Anton T, Rutherford MG, Braswell AL, Jowers MJ. A field guide to the amphibians and reptiles of Trinidad & Tobago. Port of Spain: Trinidad & Tobago Field Naturalists´Club; 2018.

6 Rivas GA, De Freitas M, Kaiser H, Barrio-Amorós CL,Barros TR. . *Amphibians of the Península de Paria*: *a pocket field guide*. Frankfurt A. M.: Fundación Thomas Merle/Oro Verde, Die Tropenwaldstiftung/Chimaira Verlag/Universidad del Zulia/The Biodiversity Consultancy. 20 pp. [folding]; 2018.

7 Schmidt KP. Reptiles and amphibians of the Mandel Venezuelan expedition. Field Mus Nat Hist (zool. ser.). 1932;37: 169-163.

8 Rivero JA. Salientia of Venezuela. Bull Mus Comp Zool. 1961; 126:1-207+one plate.

9 Kaiser H, Hardy JD, Green DM. Taxonomic status of Caribbean and South American frogs currently ascribed to *Eleutherodactylus urichi* (Anura: Leptodactylidae). Copeia 1994; 1994: 780-796.

10 Gehara M, Crawford AJ, Orrico VGD, Rodríguez A, Lötters S. et al. High levels of diversity uncovered in a widespread nominal taxon: continental phylogeography of the neotropical tree frog *Dendropsophus minutus*. Plos One. 2014; 9: e103958.

11 Linares O. Una salamandra del género *Bolitoglossa* de la cueva de Hueque, Sierra de San Luis, Venezuela. Bol Soc Venez Espel. 1974; 5:143-147.

12 Rivero-Blanco C, Schargel WE. A new diurnal gecko in the genus *Gonatodes* (Squamata: Sphaerodactylidae) from Margarita Island, Venezuela. Zootaxa. 2020; 4729: 429-439.

13 Rivas G, Rojas-Runjaic FJM, Barrio-Amorós CL. Geographic Distribution. *Pseudogonatodes manesi*. Herpetol Rev. 2006; 37:107.

14 Carvalho CM. Uma nova espécie de microteiideo do gênero *Gymnophthalmus* do estado de Roraima, Brasil (Sauria, Gymnophthalmidae). Pap Avul Zool. 1997; 40: 161-74.

15 Sánchez‐Pacheco SJ, Torres‐Carvajal O, Aguirre‐Peñafiel V, Nunes PMS, Verrastro L, Rivas GA, Rodrigues MT, Grant T, Murphy RW. Phylogeny of *Riama* (Squamata: Gymnophthalmidae), impact of phenotypic evidence on molecular datasets, and the origin of the Sierra Nevada de Santa Marta endemic fauna. Cladistics. 2017; 34:260-291.

16 Murphy JC, Jowers MJ, Lehtinen RM, Charles SP, Colli GR, Peres AK, Jr. Hendry CR, Pyron R.A. Cryptic, sympatric diversity in tegu lizards of the *Tupinambis teguixin* group (Squamata, Sauria, Teiidae) and the description of three new species. Plos One. 2016; 11(8): e0158542.

17 Carvajal-Cogollo JE, Cárdenas-Arévalo G, Castaño-Mora O. Reptiles de la región Caribe de Colombia. In: Rangel JO, editor. Colombia Diversidad Biótica XII. La región Caribe de Colombia. Bogotá: Universidad Nacional de Colombia; 2012. p. 791-812.
